# Perovskite Nanocrystal-Based Scintillators: Synthesis Routes, Matrix Engineering, and Performance Optimization for Radiation Detection

**DOI:** 10.3390/nano16201279

**Published:** 2026-10-09

**Authors:** Pavel M. Sokolov, Aleksandr A. Knysh, Irina S. Aleksandrova, Alexander V. Karaulov, Pavel S. Samokhvalov

**Affiliations:** 1 Life Improvement by Future Technologies (LIFT) Center, Skolkovo, 143025 Moscow, Russia; aaknysh@mephi.ru (A.A.K.); irina.kryukova.mephi@gmail.com (I.S.A.); drkaraulov@mail.ru (A.V.K.); p.samokhvalov@gmail.com (P.S.S.); 2 Institute for Physics and Engineering in Biomedicine, National Research Nuclear University MEPhI (Moscow Engineering Physics Institute), 115409 Moscow, Russia; 3 Department of Clinical Immunology and Allergology, Institute of Molecular Medicine, Sechenov First Moscow State Medical University ( Sechenov University), 119146 Moscow, Russia

**Keywords:** perovskite nanocrystals, scintillator, radiation detection, medical imaging, in situ synthesis

## Abstract

Semiconductor nanocrystals with a perovskite crystal structure, both fully inorganic and hybrid, have found broad application as scintillation materials over the past decade, owing to their high absorption capability, fast response time, tunable optical properties, and radiation hardness achievable through compositional and matrix engineering, as demonstrated in a large number of recent works. At the same time, scintillating materials based on perovskite nanocrystals (PNCs) are not devoid of drawbacks. These fall into two groups: intrinsic limitations—such as self-reabsorption of the emitted light caused by a small Stokes shift, and a soft ionic lattice with labile surface ligands that renders PNCs sensitive to humidity, heat, and ionizing radiation—and limitations introduced during composite fabrication, such as aggregation at the PNC loadings needed for sufficient X-ray absorption, poor compatibility between the ligand shell and the host matrix, light scattering, and matrix-induced degradation. These limitations constrain scintillator performance—particularly the light yield achievable without additional material engineering—and necessitate the development of new synthesis strategies and material architectures. The characteristics of PNC-based scintillators are determined both by the PNC synthesis route—in situ growth directly within the host material, or the use of pre-synthesized PNCs subsequently incorporated into a supporting matrix—and by compositional and functional modification strategies such as PNC doping, surface ligand engineering, or coupling of PNCs with an energy donor, acceptor, or plasmonic component. In this review, we examine how PNC chemical composition, surface ligands, synthesis route, and other fabrication strategies jointly determine the performance of the final functional scintillator, in terms of light yield, radiation detection limit, radio- and photoluminescence decay time, stability under ionizing radiation and environmental factors, optical transparency, and spatial resolution. We discuss the most problematic issues associated with these composite nanomaterials and highlight the most promising strategies for developing highly efficient, long-lasting scintillators for applications in medical imaging, radiotherapy, forensics, and quality control.

## 1. Introduction

Radiation detection plays a crucial role in non-invasive diagnostic methods in medicine [1], non-destructive testing and material inspection systems [2], security screening [3], monitoring at nuclear power plants [4], and various scientific research applications [5,6]. Today, direct and indirect conversion of X-ray radiation into an electrical signal are almost the sole approaches to X-ray detection. Direct conversion is accomplished using materials with high atomic density that efficiently absorb X-ray photons. In these materials, X-ray photons interact directly with atoms, causing ionization and generating charge carriers; these charges are then collected by electrodes to form an electrical signal proportional to the energy of the absorbed photons. Cadmium telluride (CdTe) and cadmium zinc telluride (CdZnTe) are examples of materials widely used in direct conversion detectors [7]. Indirect conversion, by contrast, involves two stages: (i) X-ray photons are first absorbed by a scintillation material, which converts their energy into visible photons (scintillation); (ii) these visible photons are then registered by a detector, which converts the light into an electrical signal. This method is employed in the majority of commercial X-ray systems due to its relative simplicity and the broad variety of available scintillation materials [8].

At the same time, no single universal scintillator is ideally suited for all applications, since each imaging or detection modality imposes a distinct combination of requirements on the scintillator material. Positron emission tomography (PET) requires scintillators with fast decay times, high light yield, and high stopping power at the 511 keV annihilation energy, in order to achieve adequate timing and coincidence resolution [9]. Computed tomography (CT), by contrast, places the greatest emphasis on extremely low afterglow and high temporal stability, since residual luminescence between successive X-ray pulses directly degrades image quality through ghosting artifacts, while light yield and decay time are less important [10]. Radioisotope diagnostics and gamma-camera imaging require scintillators with high energy resolution, to discriminate between closely spaced photopeaks [11]. Radiotherapy dosimetry imposes a different set of priorities altogether: real-time, linear dose response, and radiation hardness under cumulative doses that can reach the kGy–MGy range are typically prioritized over light yield itself [12]. Compact screening systems and non-destructive testing systems operating at low X-ray energies can rely on scintillators with weaker absorption [3], whereas screening of large cargo using gamma radiation, and detection of cosmic radiation, require scintillators with the highest possible absorption efficiency and light yield [13].

At present, a vast number of scintillators of various types and purposes exist, among which two principal classes can be distinguished. The first class comprises inorganic scintillators containing heavy atoms, which exhibit high efficiency in converting high-energy photons into visible light but have relatively slow scintillation decay. The second class comprises fast organic scintillators, which possess short fluorescence lifetimes but are incapable of efficiently absorbing X-ray or gamma radiation (Table 1). Due to their low density and low *Z*_eff_, organic scintillators are less suitable than inorganic scintillators for the detection of X-ray and gamma radiation, as well as thermal neutrons, but are better suited for detecting fast neutrons and α- and β-particles [14].

Inorganic scintillators have found wide application in medical radiography, radiation exposure monitoring, and radiation safety. Typical inorganic scintillators are single crystals of halide or oxide compounds with wide bandgaps [23]. They are additionally doped with activator atoms, whose energy level structure favors efficient conversion of ionizing radiation energy into visible light. For example, NaI:Tl and CsI:Tl are widely used owing to their high scintillation performance and radiation stability. Typically, at least one element constituting the scintillator compound has a high atomic number, no less than 50. One of the classical methods for fabricating inorganic scintillators is crystallization from a melt mixture at high temperature, a process that complicates production and hinders tuning of the scintillator’s radioluminescence to the desired region of the visible spectrum [24]. In addition, many widely used scintillators, such as NaI:Tl, are hygroscopic, necessitating waterproof packaging to prevent degradation. These factors contribute to the high cost of inorganic single-crystal scintillators, limiting their application in fields requiring large volumes of material. For a more detailed account of inorganic scintillators, their scintillation mechanisms, properties, and applications, the reader is referred to recent reviews by Dujardin et al. [25] and Yanagida et al. [26].

Owing to their fast response time, cost-effectiveness, and ease of fabricating large-area sensors, organic scintillators are widely used in high-energy physics, neutrino astronomy, medical imaging, and national security. Organic scintillators can be conventionally classified into crystalline (anthracene, stilbene, p-terphenyl, etc.), liquid (solutions of 2,5-diphenyloxazole, p-terphenyl, etc.), and plastic scintillators (oxazole or oxadiazole derivatives, polyphenyl hydrocarbons, etc., in a polymer matrix). The scintillation light yield of plastic scintillators can be enhanced by improving the efficiency of energy transfer from the host matrix to the fluorescent molecules. For a more detailed account of the compositions, properties, and application niches of organic scintillators, the reader is referred to two recent reviews by Pozzi et al. [27] and Mathews et al. [28].

Thus, inorganic and organic scintillators possess complementary properties: inorganic scintillators provide a high absorption cross-section for X-ray and gamma radiation owing to their high *Z*_eff_ and high density, but are characterized by relatively slow scintillation decay (tens to hundreds of nanoseconds or longer); organic scintillators, in contrast, exhibit an ultrafast response (a few nanoseconds) and have low production cost, but are substantially inferior in ionizing radiation absorption efficiency due to their low *Z*_eff_ (~5.7) and low density (~1 g/cm^3^). Developing a material that combines the high absorption cross-section characteristic of inorganic scintillators with the fast scintillation kinetics inherent to organic scintillators is one of the key challenges in scintillation detector development. Fluorescent perovskite nanocrystals (PNCs) are promising candidates for addressing this challenge: they possess a *Z*_eff_ on the order of 52–62 (e.g., 62 for CsPbBr_3_ and 52 for Cs_3_Cu_2_I_5_), comparable to that of inorganic scintillators such as CsI (*Z*_eff_ = 54), while simultaneously exhibiting ultrafast scintillation with decay times ranging from sub-nanosecond to a few nanoseconds—a range comparable to organic scintillators and inaccessible to most inorganic single crystals. Additional advantages of PNCs include the possibility of solution-phase synthesis at room temperature, which drastically reduces production costs compared to the high-temperature crystallization required for inorganic scintillators, as well as the ability to form scintillators as thin films, flexible composites, and structures of arbitrary shape through solution-processing and molding methods.

Typical PNCs have the chemical formula ABX_3_, where the B cation is surrounded by six X anions, and the A cation resides in the cavity formed by eight corner-sharing BX_6_ octahedra. In addition to classical 3D PNCs, double perovskite nanocrystals (DPNCs) with the formula A_2_B′B″X_6_ are also distinguished. These materials are a possible alternative to lead halide perovskites, in which toxic lead is substituted by alternating non-toxic metals (e.g., Ag^+^ and Bi^3+^). Another class of perovskite materials is vacancy-ordered perovskites (VOPs) with the formula A_2_BX_6_, which have 3D structures with isolated metal-halide octahedra formed by alternating B-site vacancies. Furthermore, there exist layered (2D) perovskites with the chemical formula L_2_A_n−1_B_n_X_3n+1_, in which inorganic layers are separated by long organic molecules (ligands), as well as low-dimensional (1D/0D) perovskites, representing chains (1D) or fully isolated molecular clusters (0D) of octahedra; these can also be obtained in nanocrystal form, although they are considerably less common than classical 3D PNCs, DPNCs, and VOPs. Inorganic and organic–inorganic (hybrid) PNCs are further distinguished depending on whether the A cation is inorganic (Cs, Rb, etc.) or organic (methylammonium, formamidinium, etc.). In inorganic and hybrid PNCs, the B cation is typically Pb^2+^, Sn^2+^, or Ge^2+^ (for classical PNCs), Sn^4+^ or Ti^4+^ (for VOPs), or Ag^+^/In^3+^ or Na^+^/Bi^3+^ pairs, while the halide ion (X) is Cl^−^, Br^−^, I^−^, or a combination thereof. 3D PNCs are the most widely used in scintillator compositions and will therefore receive primary attention here, although examples of scintillators based on other types of perovskite nanocrystals will also be discussed in this review.

Owing to their predominantly ionic structure, PNCs can be synthesized by simple mixing of precursors at room temperature, and their optical properties can be tuned by varying the halide composition [29]. For nanoscale PNCs, the quantum confinement effect also manifests, as in classical semiconductor nanocrystals such as CdSe [30]. The size, shape, and composition of PNCs can be modified post-synthesis, for example through ion exchange [29,31]; however, surface defects arising from the detachment of ligands and surface atoms can strongly affect the optical properties of PNCs after synthesis [32]. To mitigate these effects, numerous methods for post-synthetic surface treatment of PNCs and their incorporation into various matrices have been developed.

In contrast to lightweight organic and plastic scintillators, PNCs possess *Z*_eff_ > 50–60, and the mass density of the pure crystal is approximately 3.5–4.8 g/cm^3^ [33]. Consequently, PNC-based scintillators possess a high cross-section for ionizing radiation absorption, and even thin scintillator layers (~micrometers) made of PNCs are capable of efficiently absorbing X-ray radiation, for which conventional plastic scintillators would require centimeter-thick blocks. Unlike single-crystal-based scintillators, PNC-based scintillators can be fabricated in various shapes, and unlike organic scintillators, which have low ionizing radiation absorption efficiency, PNC-based scintillators can also be fabricated in the form of thin films.

The limitations of PNC scintillators fall into two groups: those that are intrinsic, arising from the physics and chemistry of the nanocrystals themselves, and those introduced during composite fabrication. Intrinsic limitations include self-absorption caused by the small Stokes shift of band-edge emission [34], a soft ionic lattice with dynamically bound ligands [35,36], which renders PNCs sensitive to heat [37], moisture [38], and radiation-induced defects [39], and which allows conversion to non-luminescent phases (e.g., Cs_4_PbBr_6_, CsPb_2_Br_5_) [40]; and lead-based compositions, which raise toxicity and environmental pollution concerns [41].

Composite-induced limitations, by contrast, arise when PNCs are combined with a host matrix: the high PNC loading required for efficient X-ray absorption promotes aggregation and concentration quenching of fluorescence, reducing both PLQY and transparency [42]; a mismatch between the hydrophobic ligand shell and the host can cause phase separation, inducing undesirable light scattering and loss of transparency and spatial resolution [43]; the fabrication environment itself (solvents, initiators, temperature, trapped oxygen and water) can lower PLQY upon embedding [44]; and dilution by a low-Z matrix reduces the composite’s overall absorption efficiency [42].

Fundamental limits—which can be addressed only by changing the PNC’s composition, dimensionality, or surface chemistry—must therefore be distinguished from engineering problems, which can in principle be solved through better choice of host, ligands, and processing conditions. The corresponding strategies are discussed in the main sections of this review: in situ growth mainly addresses composite-induced limitations (aggregation during loading, ligand–host incompatibility) while delegating protection against intrinsic instability to the host material. Separating PNC synthesis from composite fabrication allows finer control over the nanocrystal’s intrinsic properties (size, composition, surface chemistry), but re-exposes the PNCs to composite-fabrication-induced losses during loading. Matrix-free approaches to PNC scintillator fabrication avoid host-induced losses and dilution altogether, but leave the issues stemming from the intrinsic instability of perovskites unaddressed.

PNC-based scintillators have been actively developed and improved over the past decade, as reflected not only in the large number of research articles but also in the numerous reviews published on this topic. In this review, we explicitly compare two fundamentally different approaches to fabricating composite scintillators: in situ synthesis of PNCs during scintillator formation, and incorporation of pre-synthesized PNCs into a composite host matrix. This comparison clarifies how the synthesis route itself affects the resulting scintillation properties, and which modifications of the PNC structure and host matrix are achievable within each approach.

Readers interested in the fundamental principles underlying perovskite-based scintillators, as well as in scintillators based on bulk single crystals and microcrystals, are referred to the recent review by Simonenko et al. [45]. The review by Wang et al. [46] places particular emphasis on the trade-off between light yield and response time, as well as on radioluminescence engineering strategies involving stacked structures, waveguiding, chiral circularly polarized luminescence, increased transparency, and related approaches. Readers interested in specific application examples of PNC-based scintillators—including high-resolution X-ray imaging, ultrafast X-ray detection and imaging, and multi-energy X-ray imaging—are referred to the review by Huang and Yang [47], which discusses these and other applications in detail.

Unlike these reviews, the present work is organized around the two PNC–matrix fabrication routes, rather than around performance parameters or application domains, directly linking synthesis strategy to the resulting trade-offs in light yield, spatial resolution, decay time, and stability discussed throughout this review.

In the main body of the review, we discuss mechanisms for mitigating these adverse effects, both through modification of the PNCs themselves and through various strategies for incorporating PNCs into a host matrix. At this point, however, it is worth stating the central idea: no single universal strategy can resolve all of these limitations simultaneously—in most cases, improving one parameter comes at the expense of another. These trade-offs arise at two levels: the choice of host matrix, and the modification of the PNCs themselves.

At the matrix level, polymer hosts enable the fabrication of flexible, large-area scintillators suitable for non-planar detector geometries, but may suffer from reduced PLQY, PNC aggregation, and insufficient moisture protection. Glass and silica hosts, by contrast, provide strong resistance to humidity and elevated temperature and allow matching of the refractive indices of the PNCs and host, making them preferable for harsh operating conditions, though they do not always yield high photoluminescence quantum yield. Metal–organic frameworks (MOFs) and other porous hosts allow high PNC loading, which is beneficial for applications requiring strong X-ray absorption, but offer comparatively poor protection against moisture. Structured hosts such as anodic aluminum oxide (AAO) templates provide some of the highest reported spatial resolutions—critical for microscopy and defect-inspection applications—but preclude the fabrication of large-area scintillators and remain limited in light yield.

The second level of trade-offs concerns not the host matrix, but modification of the PNCs themselves, together with fundamentally different scintillator architectures. Surface modification of PNCs with specific ligands improves PLQY and host compatibility by suppressing luminescence quenching and PNC aggregation at high loading, thereby broadening the range of compatible hosts; however, achieving the optimal density of modified ligands remains challenging for both in situ-grown and pre-synthesized PNCs.

Doping of PNCs likewise shows divergent trade-offs depending on the dopant type. Doping with transition metals, most notably Mn^2+^, exploits a parity-forbidden d–d transition to produce a large Stokes shift and suppressed self-absorption, enabling thicker or more densely loaded scintillators with high light yield—but at the cost of a substantially longer luminescence decay time (reaching the millisecond range), which limits applicability in scenarios requiring a fast response, such as dynamic imaging. Doping with lanthanide ions produces a more heterogeneous set of effects: it can improve control over crystallization and environmental stability of the PNCs, and in some cases (e.g., Lu^3+^ doping) even accelerate the luminescence decay by reducing lattice defect density, whereas other ions (e.g., Eu^3+^) introduce only a moderate lengthening of decay time while simultaneously improving spectral characteristics.

A more generally applicable strategy is the introduction of additional energy-transfer pathways into the scintillator—for example, by adding dyes or conventional organic scintillators—since this suppresses self-absorption regardless of PNC composition. However, it requires additional fabrication steps and can lead to an undesirable increase in decay time and a reduction in the composite’s average effective atomic number (*Z*_eff_). Plasmonic (Purcell) enhancement, in which PNC luminescence is accelerated in the presence of metal nanoparticles, can simultaneously shorten decay time and increase emission intensity, but is accompanied by substantial non-radiative energy losses to the metal and by light scattering from the nanoparticles themselves, which degrades both spatial resolution and scintillator transparency. Finally, matrix-free scintillators maximize X-ray absorption and achieve dense PNC packing for improved spatial resolution, making them attractive for thin, high-resolution detector layers—but the resulting materials are fragile, limited in thickness, and highly susceptible to environmental degradation.

Before examining in detail how PNC synthesis methods and incorporation into scintillators affect their properties, we offer a short introduction to the parameters of scintillation materials for radiation detection.

## 2. Parameters of Scintillation Materials for Radiation Detection

As charged particles, including electrons, positrons, protons, etc., propagate through matter, their kinetic energy is dissipated mainly through excitation and ionization of the surrounding atoms and molecules. This process is characterized by a parameter known as *stopping power*—the average amount of energy that ionizing radiation loses per unit path length when traveling through a specific material. Stopping power depends on both the properties of the incident particle and the average atomic number, density, and electron configuration of the interacting medium, and is described by Bethe–Bloch formalism [48].

For uncharged radiation, such as X-rays and γ-rays, the interaction with matter is described differently, since they do not undergo continuous energy loss along their trajectories. Instead, the ability of a material to attenuate γ-ray radiation is commonly characterized by the reduction in photon-beam intensity with increasing penetration depth, which follows an exponential attenuation law. The *mass attenuation coefficient* (in cm^2^/g) quantifies the probability of photon interaction per unit mass of a material and is fundamentally based on the Beer–Lambert law (Equation (1) in Table 2) [49].

The interaction of X-ray and γ-ray with matter occurs predominantly through three fundamental mechanisms: the photoelectric effect (photoabsorption), Compton scattering and pair production [64]. In the low-energy region up to a few hundred keV where the X-ray or γ-ray energy is well below the electron rest energy m_e_c^2^ ≈ 511 keV, photon interactions are predominantly governed by photoelectric absorption (photoelectric effect), in particular for high-Z materials. With increasing X-ray or γ-ray energy from a few MeV up to several tens of MeV, the contributions of other interaction mechanisms become progressively more important. Depending on the γ-ray energy and the properties of the absorbing medium, the total photon interaction cross section may include contributions from photoelectric absorption (σ_pe_), coherent (Rayleigh) scattering (σ_coh_), incoherent (Compton) scattering (σ_incoh_), pair production (σ_pair_), triplet production (σ_trip_) and photonuclear reactions (σ_ph.n._) (Equations (2)–(7)) [50,52,53,65]. Among the photon interaction mechanisms, photoelectric absorption is particularly important for scintillation detection, as it favors the deposition of the full photon energy within the detector volume. The *photoelectric absorption cross section* characterizes the probability of interaction between an incident photon and an atom of the scintillator material [66]. In the photoelectric absorption process, the incident photon transfers its entire energy to a bound atomic electron and is completely absorbed, while the electron is ejected from its atomic shell as a photoelectron. The photoelectric absorption cross section [51] exhibits a strong dependence on both the incident photon energy and the atomic number of the absorbing element and can be expressed at low X-ray energies as Equation (4).

The use of high-Z materials is particularly beneficial for low- and intermediate-energy spectroscopy, since their enhanced photoelectric absorption probability provides more efficient photon energy deposition. In the case of multicomponent scintillator material, its elemental composition can be represented by an *effective atomic number* (*Z*_eff_), which can be estimated using Equations (8)–(11). At higher photon energies, a sufficiently high material density becomes increasingly important for reducing the *radiation length* (*X*_0_), which represents the characteristic distance over which the energy of a high-energy electron decreases to 1/e of its initial value (Equation (12)) [58].

The performance of a scintillator cannot be adequately described by a single characteristic. An efficient scintillation material should combine a high probability of interaction with ionizing radiation, effective conversion of the absorbed energy into scintillation photons, a fast temporal response and sufficient stability under operating conditions. However, no single scintillator simultaneously exhibits optimal values for all relevant characteristics. Therefore, the selection of a suitable material is inherently application-dependent. In practice, selection of the scintillator requires a compromise among multiple physical, optical, and operational properties, including material density (ρ), *light yield*, *scintillation decay time*, spectral compatibility with the photodetector, and resistance to radiation-induced degradation. Economic and technological factors, particularly material cost, manufacturability, and scalability, must also be considered, especially for detector systems requiring large volumes of scintillating material.

The key characteristics of scintillators that determine their performance in the detection of ionizing radiation include:

(i) *Light yield (LY)*—the number of scintillation photons generated per unit of deposited energy (photons/MeV). When evaluating the light yield of a scintillator, a distinction should be made between the absolute and technical light yield. The absolute light yield $\left(\eta \right)$ (Equation (13)) reflects the intrinsic efficiency of conversion of the energy (*E*) deposited by ionizing radiation into total energy of scintillation photons (*E*_p_), whereas the technical light yield (*T*) (Equation (14)) corresponds to the fraction of scintillation light (*L*_p_) that is effectively collected by the detector. Thus, only the scintillation light incident upon and collected by the detector’s active area contributes to the measured signal. Consequently, the technical light yield depends not only on the intrinsic scintillation efficiency of the material but also on the light-collection efficiency of the detector system.

In practice, the experimentally determined scintillation light yield depends on the measurement methodology, including the geometry of both irradiation and detection, the spectral response and photon detection efficiency of the photodetector, and the quality of optical coupling between the scintillator and detector. These experimental factors complicate direct comparison of light yield values reported in different studies. Moreover, the literature does not always explicitly distinguish between absolute and technical light yield, making it difficult to establish which value is actually reported. Therefore, unless the absolute light yield is explicitly determined, the experimentally reported values may be regarded as technical light yield. For simplicity and to avoid unnecessary terminological ambiguity, the term “light yield” will hereafter be used throughout this review to refer to such experimentally reported values. Currently, various experimental approaches are available for determining the light yield of scintillation materials. Basic formulae related to the determination of a scintillator light yield are summarized in Table 2, Equations (15)–(17).

Among the most feasible approaches for estimating a scintillator’s light yield are integrating-sphere-based measurement methods. These are particularly well suited to the task because they collect scintillation photons emitted across a wide range of directions, thereby reducing direction-dependent losses in light collection and minimizing the influence of collection geometry on the measured signal [67,68]. Alternatively, the light yield of a scintillator can be estimated by comparing its response to that of a reference material with a known light yield [63,69,70].

Table 3 summarizes the corresponding methods (Equations (18)–(24)), which are based primarily on experimentally measurable quantities and account for factors such as sample geometry and thickness, radiation absorption, optical collection geometry, and photodetector response. Among these, the methods described by Equations (21), (23) and (24) are particularly well suited for quantitative light yield determination. Equation (21) accounts for the fraction of deposited X-ray energy, the wavelength-dependent detector efficiency, and the irradiated area, providing a robust basis for comparing different scintillators. Equation (23) determines the light yield from the position of the Compton edge relative to a reference scintillator, correcting for spectral mismatch between the scintillator’s emission and the photodetector’s response. Equation (24) directly relates the electrical response of a SiPM to the light yield, allowing the measured charge to be converted into photons emitted per unit of deposited energy.

Alongside the conventional semi-empirical relationships, considerable effort has recently been directed toward the development of alternative theoretical and computational approaches for estimating scintillation light yield. Such methods aim to account more explicitly for the fundamental processes governing energy deposition, charge-carrier generation and transport, and radiative recombination in the scintillator, thereby providing a more comprehensive prediction of its efficiency [77].

(ii) *Detection limit and response linearity*—for low-dose X-ray detection, the minimum detectable dose rate represents an important performance parameter and corresponds to the lowest radiation level at which the scintillation signal can be reliably distinguished from background noise. This characteristic is particularly relevant to medical imaging, where a lower detection limit may enable diagnostically useful images to be acquired at reduced patient radiation doses [58]. In addition, an effective scintillator should exhibit a linear dose response, with the radioluminescence intensity remaining proportional to the absorbed dose or dose rate over a broad dynamic range without significant saturation. Such linearity enables accurate quantification of the incident radiation and ensures reliable detector operation across a wide range of exposure conditions [78].
(iii) *Scintillation decay time*—the scintillation decay time is commonly defined as the characteristic time over which the scintillation intensity decreases to 1/*e* (~37%) of its initial value. It characterizes the temporal response of a scintillator following energy deposition by ionizing radiation. In practice, the scintillation intensity *I*(*t*) rarely follows a single exponential decay and is instead fitted with a sum of exponential terms, with the number of terms—one, two, or three—chosen to reflect the number of physically distinct relaxation pathways contributing to the observed emission time trace [76]. A single-exponential decay is expected when the scintillation light originates from one dominant, well-defined radiative recombination channel, typically direct (free) exciton recombination in a largely defect-free crystal or nanocrystal, and is most often observed at low excitation density, where competing non-radiative and trap-related pathways are suppressed [79]. Bi-exponential (two-component) decay is the most commonly reported behavior for perovskite nanocrystal scintillators. It reflects the coexistence of two distinct emitting populations: a fast component (sub-nanosecond to a few nanoseconds), typically assigned to prompt, direct free-exciton recombination, and a slower component (nanoseconds to microseconds). The slower component is usually attributed either to delayed recombination—following the trapping and subsequent thermally activated release of charge carriers at shallow defect states—or, in materials that exhibit self-trapped-exciton emission, to the intrinsically slower radiative pathway associated with the lattice-distorted excited state [58,80].

A third exponential term becomes necessary when the decay includes an additional, kinetically distinct process that a two-component fit cannot capture. Such cases include deep trap states with release rates much slower than those of the shallow-trap component, multi-step energy transfer in composite or doped scintillators, or size and compositional inhomogeneity that gives rise to a genuinely separate third population of emitters with its own characteristic lifetime. A direct example relevant to lead halide PNCs is the time-resolved radioluminescence decay of Cd-substituted CsPbCl_3_ nanocrystals, which is well described only by a tri-exponential function—reflecting multiple hole-trapping and detrapping pathways associated with undercoordinated surface chloride defects [81].

Because adding exponential terms always improves the mathematical goodness of fit, the number of components used (one, two, or three) should be justified statistically rather than fixed in advance—for example, by comparing the reduced *χ^2^* values or residuals across successive fits. The resulting time constants are typically combined into a single average decay time, weighted by the relative amplitude and duration of each component, allowing comparison between studies that report different numbers of fitted components.

These decay parameters determine the scintillator’s response speed and directly influence the maximum counting rate the detection system can support. Longer decay times, in particular, increase the likelihood of temporal overlap between successive scintillation pulses (pulse pile-up), which limits detector performance at high counting rates. This makes decay time a critical parameter for applications requiring precise event timing, such as time-of-flight (TOF) techniques [82], which are widely used in biomedical imaging.

(iv) *Afterglow* is the residual scintillation emission that occurs after irradiation was terminated, and is generally associated with the delayed release of carriers from trap states. It is usually expressed as the residual light intensity (or electrical current) relative to its steady-state value, measured at time t after the radiation source is turned off: Afterglow (%) = *I_t_*/*I_0_* × 100%. In conventional real-time imaging, prolonged afterglow is undesirable, since signals generated during previous exposures overlap with subsequent frames, resulting in ghosting and reduced temporal fidelity [83]. This property is especially important for computed tomography, dynamic X-ray imaging, and high-rate radiation detection.
(v) *Optical transparency and self-absorption* are the factors that govern light transport and extraction from the scintillator. The transparency of a scintillating material is commonly quantified in terms of the optical attenuation length, corresponding to the distance traveled by scintillation photons before their intensity is reduced to 1/*e* (~37%) of its initial value. High transparency and low self-absorption facilitate efficient photon extraction and suppress lateral photon diffusion and optical crosstalk, which are critical for preserving spatial resolution in scintillator-based imaging systems [72,84,85].
(vi) *Spatial resolution*. The spatial resolution of a scintillator-based imaging setup is commonly characterized by the modulation transfer function (MTF). MTF describes how much of the contrast present in the original object is preserved in the image as a function of spatial frequency—in other words, how faithfully the imaging system reproduces increasingly fine spatial details without blurring them [86,87]. An MTF value of 0.2 is commonly adopted as a practical criterion for assessing the limiting spatial resolution of an imaging system, meaning that only 20% of the object’s original contrast is still distinguishable in the image at that spatial frequency [72]. The spatial frequency at which the MTF drops to this level, typically expressed in line pairs per millimeter (lp/mm), provides a quantitative measure of the imaging system’s spatial resolving power—that is, the finest level of detail it can still resolve.

This spatial resolution is governed not only by the intrinsic optical properties of the scintillator but also by its shape, thickness, optical transparency, and other factors. As a consequence, increasing scintillator thickness generally enhances X-ray absorption efficiency but may simultaneously increase lateral light scattering, thereby degrading spatial resolution. This trade-off between radiation absorption efficiency and spatial resolution when selecting scintillator thickness represents a key consideration in the design and optimization of scintillator screens [88].

(vii) *Emission spectrum and spectral matching* refer to the compatibility between the scintillator’s emission and the spectral sensitivity of the photodetector. An optimal scintillator should emit within the range of maximum spectral sensitivity of the selected photodetector, such as a photomultiplier tube, silicon photomultiplier, photodiode, or CMOS sensor [89]. Spectral mismatch reduces the number of detected scintillation photons even when the material’s intrinsic scintillation yield is high. This factor must therefore be taken into account when comparing different scintillators experimentally [90].
(viii) *Radiation hardness and long-term thermal, chemical, and mechanical stability* describe the ability of a scintillator to retain its optical and scintillation properties under prolonged or high-dose exposure to ionizing radiation and environmental factors. Radiation-induced defects can act as trapping centers, promote nonradiative recombination, alter optical absorption, and ultimately lead to a progressive reduction in light yield [91]. In addition to radiation resistance, long-term environmental stability is essential for reliable detector operation [92]. Hygroscopicity, oxidation, thermal degradation, phase instability, and susceptibility to moisture can significantly compromise the performance and operational lifetime of otherwise promising scintillator materials. These stability requirements, together with technological considerations related to material fabrication, represent important factors in the practical implementation of scintillators [93].
(ix) *Manufacturability and scalability*—feasibility of producing large-area or bulk scintillators with reproducible properties. Currently, most scintillation-based detection systems rely on inorganic single crystals, which offer high detection efficiency and relatively slow scintillation response. However, their fabrication typically involves costly and energy-intensive high-temperature crystal growth from the melt, whereas the production of large-volume crystals remains technologically demanding and poses significant challenges for large-scale manufacturing [94].

Thus, no single parameter can provide a comprehensive assessment of scintillator performance: an efficient scintillator should combine high stopping power and light yield with a fast, proportional response, suitable emission characteristics, low afterglow, high spatial resolution, and sufficient chemical, mechanical, and radiation stability. These parameters, however, are not independent of one another, and improving one routinely comes at the expense of another.

For instance, improving X-ray absorption calls for a thicker scintillator layer or higher PNC loading; however, denser loading promotes nanocrystals aggregation, which quenches luminescence and lowers light yield, while increased thickness itself causes more scattering of the emitted light and its lateral spread, degrading spatial resolution. Avoiding self-absorption, in turn, typically requires emitters with a large Stokes shift—self-trapped excitons or heavy dopants, rather than direct free-exciton emission—but the underlying emission mechanism, involving lattice relaxation or energy transfer to a trap or dopant state, is intrinsically slower than direct recombination; the price of suppressing self-absorption is therefore usually a longer decay time. Improving environmental stability calls for a dense, rigid inorganic host, such as glass or silica, that seals the PNCs from moisture and heat; however, such hosts require high-temperature processing and yield rigid, less readily shaped scintillators, whereas polymer hosts are easy to process at low temperature and allow flexible, large-area geometries, but protect the PNCs less effectively against moisture. Finally, self-absorption can also be suppressed without modifying the PNC itself, by introducing a second, spectrally shifted emitter—a dye, a second scintillator, or a plasmonic nanostructure—but this adds an extra material and interface whose own stability, parasitic absorption or scattering, and fabrication cost must then be managed.

These trade-offs recur throughout Section 3, Section 4, Section 5 and Section 6 of this review, and motivate why the strategies discussed there are best understood as different ways of prioritizing one parameter over another, rather than as competing attempts to optimize all parameters simultaneously.

## 3. *In Situ* Fabrication of PNC-Based Scintillators

Methods for fabricating PNC-based scintillating composites can be categorized by the physical form of the nanocrystals at the start of material formation. The simplest approach is to embed pre-synthesized, or even commercially available, PNCs into a host matrix using either solution processing or polymerization. Alternatively, liquid or solid host matrix precursors can serve as the growth medium for the nanocrystals themselves, in which case PNC growth occurs in situ. Finally, matrix-free, or free-standing PNCs can function as scintillators themselves—the third approach discussed in this review.

Although it may seem counterintuitive, we choose to begin our review of recent progress in PNC-based scintillator technology with in situ-synthesized composites, in order to highlight how the overall choice of fabrication strategy can help enhance material performance.

In situ fabrication of a PNC-based composite scintillator means that the synthesis of the PNCs proceeds concurrently with the formation of the scintillator host matrix: perovskite precursors are typically mixed with surface ligands (although ligand-free routes are also possible) and with matrix components. PNC formation within the matrix is then triggered either by an external factor, such as heating or irradiation, or occurs spontaneously after a short time delay. Without additional passivation, this route often yields PNCs with a lower fluorescence quantum yield than pre-synthesized PNCs, since the nanocrystal formation process is more difficult to control precisely. In exchange, the separate PNC-loading step—which would otherwise cause PNC aggregation—can be avoided, providing reliable protection for the typically delicate nanocrystals from environmental factors and leading to their homogeneous distribution throughout the scintillator volume. The in situ synthesis of pure and doped PNCs, as well as their combination with functional elements to tailor scintillator properties, is the major topic of this section.

### 3.1. Pristine PNCs in a Matrix

In this subsection, we examine four general topics relevant to growing pristine PNCs directly within a host matrix: selection of the host polymer and PNC composition, passivation of the PNC surface, the use of glass and other inorganic hosts for improved stability, and templating of PNC growth to directly control the scintillator’s spatial resolution. Because PNCs combine a high *Z*_eff_ with the ability to form thin, flexible films, we have selected several representative works illustrating how far matrix and processing choices can promote flexibility and resolution of a composite scintillator without compromising its light yield or stability.

#### 3.1.1. Tuning PNC Composition and Selection of Polymer Matrix

The first group of studies below examines how the choice of polymer matrix and PNC composition affects the flexibility, transparency, and light yield of in situ-grown scintillators.

**Figure 1:**
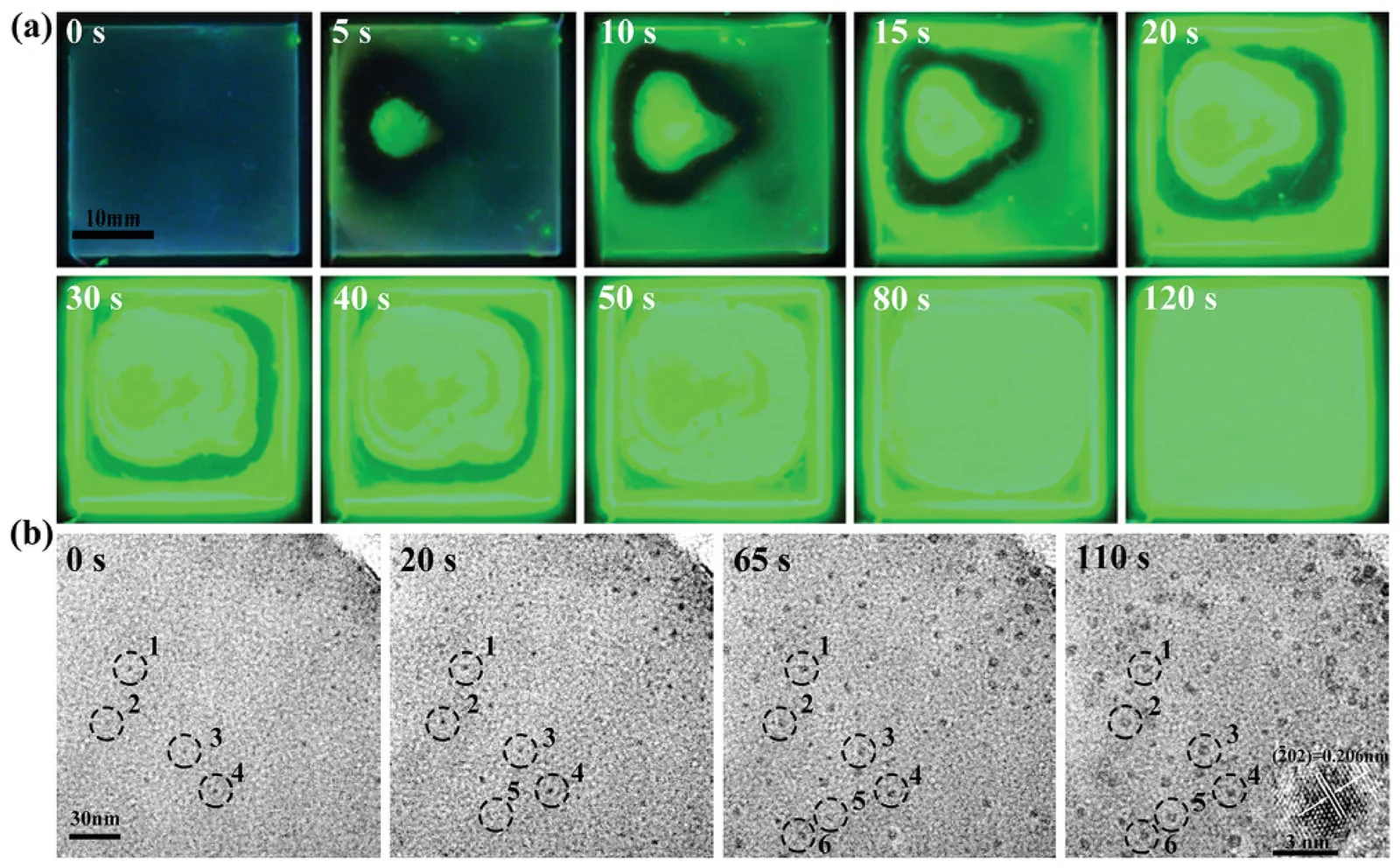
The growth process of the CsPbBr_3_-based polymer-ceramics. (**a**) Photos of the CsPbBr3 embedded polymer film placed on the hotplate at 80 °C recorded from 0 to 120 s under UV illumination. (**b**) TEM images show the polymer film before nucleation and the subsequent nucleation and growth of the CsPbBr_3_ PNCs under the electron irradiation, E-beam intensity is 2.7 × 10^2^ e^−^ Å^−2^ s^−1^. The inset of the TEM image captured at 110 s is the HR-TEM image of the corresponding precipitated CsPbBr_3_ PNCs. Nanoclusters observed at 0 s as the pre-nuclei marked with circles. Reprinted with permission from Ref. [95]. Copyright © 2021 Wiley-VCH GmbH.

Chen et al. fabricated a flexible scintillator via in situ synthesis of CsPbBr_3_ PNCs in a pre-solidified flexible polymethyl methacrylate (PMMA)-based matrix [95]. A highly supersaturated mixture of CsBr and PbBr_2_ in DMF (N,N-dimethylformamide) was added to a PMMA solution in DMF and spin-coated onto glass. The substrate was then placed on a hotplate heated to 80 °C for approximately 2 min to allow annealing and PNC growth. Formation of PNCs within the matrix was confirmed by Fourier-transform infrared (FTIR) spectroscopy on thin-film samples before and after annealing, as well as by transmission electron microscopy (TEM) and photoluminescence studies of the scintillator (Figure 1). The minimum detectable dose was 120 nGy/s, significantly lower than—and thus outperforming—the typical dose rates used in medical diagnostics (5.5 µGy/s) [96].

Notably, the authors achieved recovery of the scintillator’s radioluminescent properties after prolonged (3-h) exposure to intense radiation (8 mGy/s) by heating the already-formed scintillator at 80 °C for 4 h, demonstrating up to 50 such recovery cycles. Using transmission electron microscopy, they showed that this re-annealing step induces recrystallization of PNCs damaged by X-ray radiation. The light yield of the resulting scintillator was 15,800 photons/MeV. The authors further demonstrated that bending the scintillator to conform to the sample shape improved spatial resolution compared to a flat scintillator (up to 12.5 lp/mm vs. 1.98 lp/mm), owing to reduced light scattering.

For comparison, a control scintillator—prepared by embedding pre-synthesized PNCs into an identical matrix—exhibited much lower transparency, greater susceptibility to degradation under humid conditions, and lower spatial resolution in the resulting images.

The in situ PNC synthesis approach applies not only to inorganic PNCs but also to hybrid organic–inorganic and alloyed PNCs, as demonstrated by Saiji et al. They investigated the growth of PNCs with compositions MAPbBr_3_ (MA = methylammonium), CsPbBr_3_, CsPbBr_0.75_I_2.25_, and CsPbClBr_2_ by mixing the corresponding precursors with poly(ethylene terephthalate) (PET) fibers [97]. PET fibers were soaked in a perovskite precursor solution—MABr and PbBr_2_ in N,N-dimethylformamide (DMF) for MAPbBr_3_; CsBr and PbBr_2_ in DMF for CsPbBr_3_; CsBr, PbBr_2_, and PbI_2_ in DMF for CsPbBr_0.75_I_2.25_; and PbBr_2_, CsCl, and PbCl_2_ in dimethyl sulfoxide (DMSO) for CsPbClBr_2_—containing octylamine (for MAPbBr_3_ PNCs) or oleic acid and octylamine ligands (for the remaining compositions). A subsequent high-temperature swelling step in an autoclave (160–200 °C, 50–75 min) enables infiltration of the precursors into the polymer. Crystallization of the PNCs is then triggered by antisolvent treatment with toluene at 85 °C for 15–30 min, which displaces residual solvents from the matrix.

The fluorescence lifetimes of scintillators containing MAPbBr_3_, CsPbBr_3_, and CsPbClBr_2_ PNCs were 15.25, 30.25, and 9.21 ns, respectively, and all samples showed good stability under water immersion and heating. The MAPbBr_3_-PET sample fully recovered its luminescent properties after heating to 150 °C and subsequent cooling, and recovered up to 48% of its photoluminescence after heating to 200 °C. This sample also retained 94% of its luminescence after 3 days of incubation in water at 85 °C. The MAPbBr_3_-PET fiber composite exhibits a narrow photoluminescence peak at 539 nm, whereas X-ray excitation induces a red-shifted, broadened radioluminescence—a result of strong interactions between high-energy carriers and the perovskite lattice, which alter exciton recombination dynamics.

The light yield of the MAPbBr_3_-PET scintillator was approximately 23,000 photons/MeV, and its radioluminescent properties were similarly recovered after three cycles of heating to 167 °C and cooling to 27 °C. The scintillation decay curve of the MAPbBr_3_-PET fiber composite has an average lifetime of 186.5 ns, derived from three exponential components: fast (6.2 ns, 44%), intermediate (47 ns, 27%), and long (590 ns, 29%). Compared to photoluminescence, the longer radioluminescence decay reflects the greater complexity of high-energy X-ray excitation processes, which lead to slower recombination rates and additional relaxation pathways.

There are also examples of fabricating perovskite composites using matrices composed of a mixture of different polymers. Li et al. employed the symmetric triblock copolymer Pluronic P123—composed of poly(ethylene glycol) (PEG) and poly(propylene glycol) (PPG) in an alternating linear arrangement, PEG–PPG–PEG—to fabricate photoelectric X-ray sensors based on Cs_2_AgBiBr_6_ (CABB) PNCs [98]. The PEG segments selectively coordinate with Ag^+^ ions, directing crystal evolution toward the stable 3D phase, while the PPG segments create steric effects that reduce excessive Ag^+^ coordination and promote uniform crystal growth. This cooperative mechanism enables the production of crack-free perovskite films with high crystallinity and mechanical flexibility.

A thin film approximately 100 μm thick served as the basis for a photoelectric X-ray detector, with the film sandwiched between ITO and ITO-PEN (indium tin oxide–polyethylene naphthalate) layers. The composite film was prepared by pre-mixing PNC precursors in DMSO at 60 °C for 2 h, then adding a P123 solution in DMSO (up to 10 wt%) and stirring at 60 °C for a further 2 h. To induce PNCs growth, the resulting mixture was added dropwise to isopropanol, after which the precipitate was separated by centrifugation and deposited onto an ITO-PEN substrate.

A 100 μm film absorbs ~73.6% of 30 keV X-rays, confirming strong attenuation of the incident radiation. P123 modification improves carrier transport, enabling fast switching (rise/decay times of 267/732 ms at 500 μGy/s) that remains stable over 15 days. The device based on the P123/CABB PNC composite achieves a sensitivity of 244.71 μC Gy^−1^ cm^−2^ and a detection limit of 121 nGy/s under low bias—outperforming a comparison device in which pristine CABB PNCs were deposited directly onto the ITO-PEN substrate, owing to enhanced interfacial charge transport. The composite device also retains 80% of its sensitivity after 500 bending cycles and sustains a stable response over 60 days, whereas the unmodified device degrades rapidly. Although this work focused on photoelectric X-ray detection, it seems likely that a similar approach could be applied to fabricate crack-free PNC-based scintillator films.

Lv et al. examined how three different polymer matrices—PMMA, polyvinyl alcohol (PVA), and polyvinylidene fluoride (PVDF)—influence the in situ growth of FAPbI_3_ (FA = formamidinium), CsPbI_3_, CsPbBr_3_, and Cs_3_Cu_2_I_5_ PNCs, and how this in turn affects the properties of the resulting scintillators [99]. In their synthesis procedure, the polymer was first dissolved in a suitable solvent; a pre-prepared precursor mixture was then added, and the resulting solution was spin-coated onto a substrate. The films were subsequently heated to promote PNC growth. The authors also tested the effect of adding vitamin B1 (VmB1) to the precursor mixture, which promoted growth of larger nanocrystals with fewer surface defects and increased the average light yield by 80% relative to films prepared without VmB1. VmB1 addition also produced a slight shift in the emission peak, since halogen atoms in the perovskite lattice were partially replaced by Cl− ions originating from the VmB1 molecule. Density functional theory calculations of charge distribution and surface adsorption energy showed that the strength of the polymer–perovskite interaction depends on the electron-donating capacity of the polymer’s functional groups and on bond strength.

**Figure 2:**
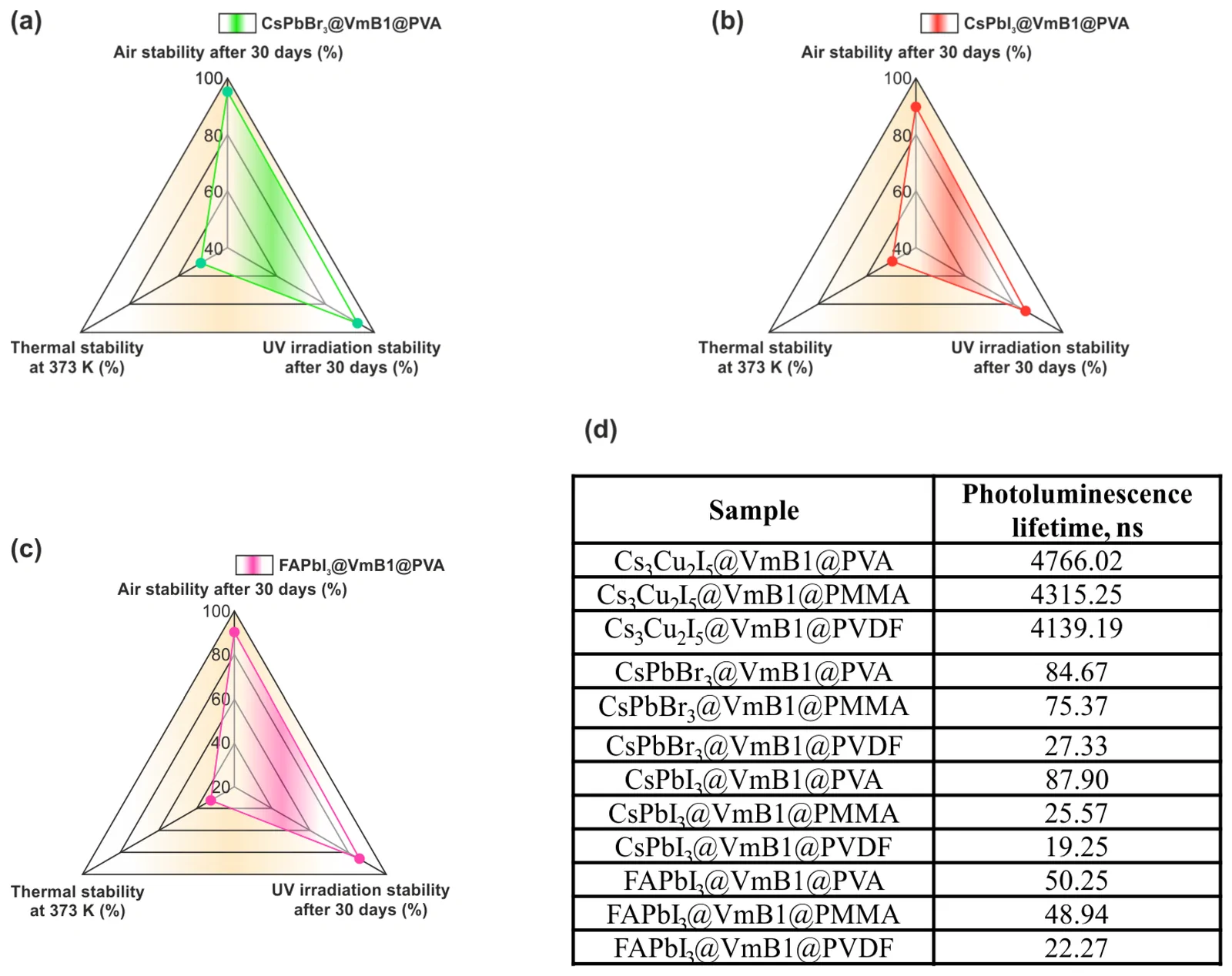
Comparison of the stability parameters of PNCs/vitamin B1 (VmB1) composites in different host matrices. The stability of (**a**) CsPbBr_3_@VmB1@PVA, (**b**) CsPbI_3_@VmB1@PVA, (**c**) FAPbI_3_@VmB1@PVA films prepared by in situ growth and (**d**) the average photoluminescence lifetime of PNC@VmB1@polymer films. Adapted from Ref. [99].

Among the three matrices tested, PVA performed best overall: PNCs of all four compositions grown in PVA showed the highest PL intensity and quantum yield (PLQY), the longest luminescence lifetimes, and the greatest stability compared to those grown in PMMA or PVDF (Figure 2). The polymer matrix also had a distinct effect on thermal, environmental, and UV-irradiation stability; notably, the Cs_3_Cu_2_I_5_@VmB1@PVA scintillator retained more than 95% of its initial luminescence intensity after 30 days of combined air exposure and UV irradiation. The superior PLQY and longer fluorescence lifetime observed for PVA-hosted PNCs, compared to the weaker performance in PVDF, were attributed to differences in interaction energy between the polymer host and the PNC surface: the stronger interaction between PVA and the PNCs suppresses both nanocrystal aggregation and luminescence quenching.

The choice of polymer matrix was also found to influence the crystal phase adopted by the PNCs. CsPbI_3_ crystallized in the cubic α-phase within both PMMA and PVA, but adopted the orthorhombic γ-phase in PVDF—a result of the lower interaction energy and lower activation energy for growth in this matrix. For FAPbI_3_, the trend reversed: PVDF suppressed formation of the intermediate δ-phase, which was not observed in the other matrices. Taken together, these optimizations enabled the Cs_3_Cu_2_I_5_@VmB1@PVA scintillator to achieve a light yield of 55,521 photons/MeV (versus 30,786 photons/MeV without VmB1) and a high imaging resolution of up to 14.0 lp/mm.

In a follow-up study [100], the same authors investigated in more detail how VmB1 addition during fabrication affects the properties of a scintillator based on CsPbBr_3_ PNCs in a PMMA matrix. They prepared a precursor mixture (0.6 mmol CsBr, 0.4 mmol PbBr_2_, and x mmol VmB1, where x = 0, 0.02, 0.04, 0.06, and 0.08) in DMF, and added 900 μL of this mixture to 5 mL of a PMMA solution in DMF. The resulting mixture was then spin-coated onto quartz glass and heated to form PNCs within the polymer.

As the VmB1 doping concentration increased, the luminescence color of the CsPbBr_3_@xVmB1@PMMA film showed slight changes but continued to exhibit green emission overall. The resulting scintillator retained 96.5% of its luminescence intensity after 480 h of immersion in an aqueous NaCl solution (30 mg/mL), and at 90 °C remains 34% of initial intensity at room temperature. Addition of VmB1 to the PNC precursor mixture improved nanocrystal structure by accelerating crystallization, reducing grain boundaries, and eliminating surface defects. The photoluminescence lifetime increased from 7.61 ns for PNC samples without VmB1 to 15.66 ns as the VmB1 concentration increased up to 10%, then decreased again to 6.56 ns for samples containing 20% VmB1.

The authors propose that VmB1 increases the fluorescence lifetime by suppressing non-radiative recombination through passivation of surface defects. VmB1 also slows the crystallization rate and directs nanocrystal connection into larger grains with fewer boundaries, thereby hindering the formation of nanocrystal clusters in which excitons would otherwise be quenched through energy transfer between closely spaced particles. With the addition of 10% VmB1, the luminescence intensity doubled, the light yield reached 9001 photons/MeV with a high spatial resolution of up to 9.0 lp/mm, and the radioluminescence decay time for the 10%-VmB1 samples was 12.99 ns.

#### 3.1.2. In Situ PNC Surface Passivation Strategies

A second group of studies addresses passivation of PNC surface defects directly—either by eliminating the need for surface ligands altogether, or by applying a dedicated passivation treatment after the matrix has formed.

Surface ligands are typically responsible for passivating surface defects and improving the morphology and optical properties of nanocrystals. However, some in situ synthesis routes achieve PNC formation without any additional surface ligands. Zhang et al. developed such a ligand-free approach for the in situ synthesis of CsPbBr_3_ PNCs within polymer membranes [101]. In their method, CsBr and PbBr_2_ were dissolved in DMSO, and the resulting precursor solution was filtered through a nylon membrane, then heated at 120 °C for 10 min under an N_2_ atmosphere; this caused the solvent to evaporate and CsPbBr_3_ to crystallize. The process yielded PNCs 10–20 nm in size (averaging 14.3 nm) grown directly on the nylon fibers, to which they remained firmly bound—a bond robust enough to survive 12 h of ultrasonic treatment of the membrane. The space between the fibers was then filled by infiltrating polydimethylsiloxane (PDMS) under vacuum, followed by heat treatment at 80 °C for 2 h, producing a CsPbBr_3_–nylon–PDMS composite.

The mesoporous nylon membrane served a dual purpose: it provided physical space for PNC crystallization, and its functional groups passivated PNC surface defects. This resulted in PNCs with a PLQY of up to 79.2%, a fluorescence lifetime of 11.8 ns, and overall improved stability. Notably, the ligand-free PNCs embedded in the nylon membrane showed no loss of fluorescence intensity after 288 h at 30–50% relative humidity, whereas control samples synthesized with conventional surface ligands lost up to 40% of their fluorescence intensity over the same period. In effect, the nylon matrix takes on the role normally played by organic ligands, passivating surface defects and shielding the PNCs from external influences. As a scintillator, the CsPbBr_3_ PNC–nylon membrane achieves a high spatial resolution of 10.4 lp/mm and a detection limit of approximately 1.2 µGy_air_/s—performance comparable to that of “classic” PNCs synthesized using conventional surface ligands.

Post-synthetic passivation provides an alternative strategy for suppressing PNC surface defects, one that does not depend on a ligand-free matrix. Li et al. developed a simple post-passivation method for a CsPbBr_3_/polypropylene (PP) composite film based on treatment with an octylamine solution, which markedly increased both photoluminescence and radioluminescence intensity [102]. Their fabrication relied on a solvent-free polymer melt encapsulation method, which avoids the micropore formation normally caused by solvent evaporation. A mixture of PP, lead stearate, cesium acetate, and acetyltrimethylammonium bromide was melt-blended in a rheometer at 215 °C and 60 rpm for 10 min, then compression-molded at 180 °C and 20 MPa for 10 min and cooled to room temperature, yielding a 0.1-mm-thick, 10 × 10 cm CsPbBr_3_/PP composite film. For passivation, this film was immersed in an octylamine/toluene solution for 12 h at room temperature and subsequently air-dried for 12 h.

The proposed passivation mechanism works as follows. Toluene first causes the PP molecular chains to swell, creating room for octylamine to diffuse in. Once inside, the hydrophilic head group of octylamine coordinates with the PNC surface while its hydrophobic tail points outward, forming a passivation layer that remains compatible with the surrounding polymer chains. As the solvent then evaporates, the PNCs become uniformly distributed throughout the polymer, which helps prevent concentration quenching from crystal agglomeration, while the polymer matrix itself contracts to form a cohesive protective barrier around the nanocrystals. Two control experiments support this picture: immersing the composite in pure toluene alone leaves its emission properties unchanged, whereas immersing it in pure octylamine instead causes CsPbBr_3_ to decompose. The authors attribute this decomposition to protonated octylamine (–NH_3_^+^) reacting with surface Br^−^ ions, stripping bromide from the crystal surface and triggering dissociation of the perovskite lattice.

Surface passivation raised the PLQY from 54% (untreated composite) to 78% (after treatment), increased the PL intensity under 365 nm UV excitation by a factor of 27, and extended the average PL lifetime from 36 to 43 ns. X-ray photoelectron spectroscopy (XPS) showed a clear shift of the Pb 4*f* and Br 3*d* peaks toward higher binding energies after passivation, consistent with stronger binding at surface Pb^2+^ sites; this confirms that the treatment passivates uncoordinated Pb^2+^ ions, which otherwise act as the primary trapping centers responsible for non-radiative recombination. FTIR spectroscopy corroborated this picture, showing that octylamine had diffused into the PP chains themselves rather than merely adsorbing onto the film surface.

#### 3.1.3. PNCs Grown in Glass and Other Inorganic Hosts

In addition to polymer matrices, PNCs can also be grown in situ within glass and other inorganic hosts, offering an alternative route to improving the chemical and thermal stability of the composite.

While polymer matrices allow for flexible scintillator fabrication and offer reasonably reliable protection of PNCs from external factors, glass matrices represent another viable host option. Xu et al. reported an in situ synthesis route for CsPbBr_3_ PNCs in glass, aimed at producing highly stable scintillators [103]. The starting powder mixture—40% SiO_2_, 35% B_2_O_3_, 3% Na_2_CO_3_, 9% ZnO, 6% Cs_2_CO_3_, 5% PbBr_2_, and 2% NaBr (in mol %)—was homogenized for 10 h and then melted at 1150 °C for 10 min under ambient atmosphere. The resulting melt was quenched by casting onto a copper plate for rapid solidification. To relieve residual thermal stress, the as-cast glass was annealed at 350 °C for 2 h in a muffle furnace, cooled to room temperature, and cut into flat pieces measuring 10 × 10 × 2 mm^3^.

The formation of CsPbBr_3_ nanocrystals within the glass host was then induced by a separate heat treatment at 470 °C. The authors found that annealing below 450 °C produced no detectable luminescence, pointing to negligible nucleation of optically active nanocrystals at these lower temperatures, whereas annealing above 490 °C led to a marked loss of optical transparency, likely due to increased scattering from excessive nanocrystal growth or macroscopic phase separation. After crystallization, all samples were polished on both parallel faces. The authors note that this in situ glass-based approach can similarly be extended to fabricate fiber-shaped scintillators, a form factor unattainable with conventional single-crystal inorganic scintillators.

Annealing at the optimal temperature of 470 °C struck a favorable balance between PNC concentration and size, yielding a PLQY of 28.9% and a fluorescence decay time of 15.2 ns. Embedding the PNCs in glass also protected them against water-induced degradation: fluorescence intensity dropped by 17% during the first 10 h of water immersion but then held steady for the following two weeks. The glass matrix likewise preserved both emission intensity and peak width across repeated heating (to 250 °C) and cooling cycles, in stark contrast to PNCs simply deposited on the glass surface, which lost their fluorescence entirely under the same conditions. In terms of scintillation performance, the PNC@glass composite achieved a light yield of approximately 4500 photons/MeV—roughly 1.8 times lower than that of BGO (8200 photons/MeV)—alongside a radioluminescence (RL) lifetime of about 15.2 ns, some twenty times faster than that of BGO.

Scintillators containing in situ-synthesized PNCs generally show greater stability than their pre-synthesized counterparts, but further passivation of the PNC surface can enhance this stability even more. Wang et al. developed a method for synthesizing CsPbBr_3_ PNCs passivated with 3-(decyldimethylammonio)-propane-sulfonate inner salt (DPSI) and confined within mesoporous silica molecular sieves (MS) [104]. Their approach relied on solid-state synthesis: CsBr and PbBr_2_ precursors were combined with DPSI and MS, and the mixture was heated to 560 °C in a quartz cuvette under an atmospheric air pressure of 0.05 MPa before being slowly cooled to yield PNCs confined within the molecular sieves (CsPbBr_3_-DPSI/MS). Scintillator films were then fabricated by spin-coating a CsPbBr_3_-DPSI/MS solution (≈0.1 g/mL in cyclohexane) onto a glass substrate and drying at 60 °C, with the coating step repeated as needed to reach the desired film thickness.

Structural characterization supported the passivation mechanism: Brunauer–Emmett–Teller analysis revealed that the surface area of the molecular sieves dropped sharply, from 998 to 15 m^2^/g, after calcination, consistent with sealing of the pores hosting the PNCs, while X-ray diffraction (XRD) indicated that the SO_3_^−^ groups of DPSI interact directly with Pb^2+^ ions at the PNC surface. This surface passivation raised the composite’s PLQY substantially, from 27.4% to 93.2%.

Together, PNC confinement within the molecular-sieve pores and DPSI-based surface passivation produced markedly improved scintillator stability across multiple stress tests. Under heating to 333 K and 373 K, CsPbBr_3_-DPSI/MS retained 84% and 41% of its photoluminescence, respectively—substantially outperforming unpassivated CsPbBr_3_/MS, which retained only 65% and 25% under the same conditions. After 48 h of blue-light irradiation (3500 W/m^2^), CsPbBr_3_-DPSI/MS showed no loss of fluorescence intensity at all, while CsPbBr_3_/MS lost more than half of its signal. The passivated samples also proved fully resistant to water: PL intensity remained unchanged after 250 h of immersion. Taken together, the authors report that these scintillators rank among the most stable known, retaining up to 90% of their fluorescence even under the combined stress of high humidity (90%), elevated temperature (60 °C), and intense irradiation (3500 W/m^2^) sustained over 1000 h.

A related in situ strategy, although used to enhance the stability of PNCs in a light conversion LED, relies not on a dedicated passivating agent, but on a single bidentate ligand that interfaces directly with both the PNCs and the host. Ji et al. incorporated 8-amino-1-naphthalenesulfonic acid (ANA) together with SBA-15 mesoporous silica into the lead precursor solution before triggering nanocrystal nucleation via cesium-oleate injection, growing ANA-CsPb(Br/I)_3_ PNCs in situ within the SBA-15 channels [105]. The sulfonic acid and amine groups of ANA anchor, respectively, to the PNC surface and to silanol groups on the silica wall, as evidenced by a shift of the Si 2*p* XPS peak from 103.7 to 101.8 eV and the disappearance of the silica O–H vibration band. This dual-anchoring interface raised PLQY from 76.2% (unpassivated control) to 91.2%, reduced the trap density by a factor of 1.5, and improved stability retention after both 48 h in water (~83% vs. ~11% for the control) and 24 h at 100 °C (>90% vs. ~45%).

Peng et al. reported the fabrication of a scintillator by in situ synthesis of CsPbBr_3_ PNCs within a melt-quenched glassy coordination polymer [106]. To this end, they fused ZnBr_2_(bIm)_2_ crystals (bIm = benzimidazole) with a precursor solution of CsBr and PbBr_2_ in DMSO at 280 °C for 8 min, then poured the resulting mixture into a silicone mold and cooled it to room temperature. The PL QY of the resulting PNCs reached 59.86%, substantially higher than in the previously discussed work.

The authors further showed that DMSO becomes incorporated into the glassy matrix, partially replacing benzimidazole and forming a new glassy polymer structure that weakens hydrogen bonding within the network. This matrix promoted optimal migration of precursor ions during synthesis, resulting in a uniform distribution of PNCs throughout the matrix. This uniformity, in turn, ensured high scintillator transparency and a spatial resolution of 25 lp/mm—also a notably high value compared with many other PNC-based scintillators. The CsPbBr_3_@ZnBr_2_ = (bIm + DMSO)_2_ glass additionally exhibited a low softening temperature (40 °C), allowing the material to be easily bent and cut. The resulting scintillator achieved a light yield of 1202 photons/MeV and a detection limit of 514 nGy/s.

#### 3.1.4. Templated PNCs Growth for Enhanced Spatial Resolution

Finally, templating PNC growth within a structured membrane offers a way to directly control nanocrystal geometry, which in turn strongly influences the spatial resolution of the resulting scintillator.

**Figure 3:**
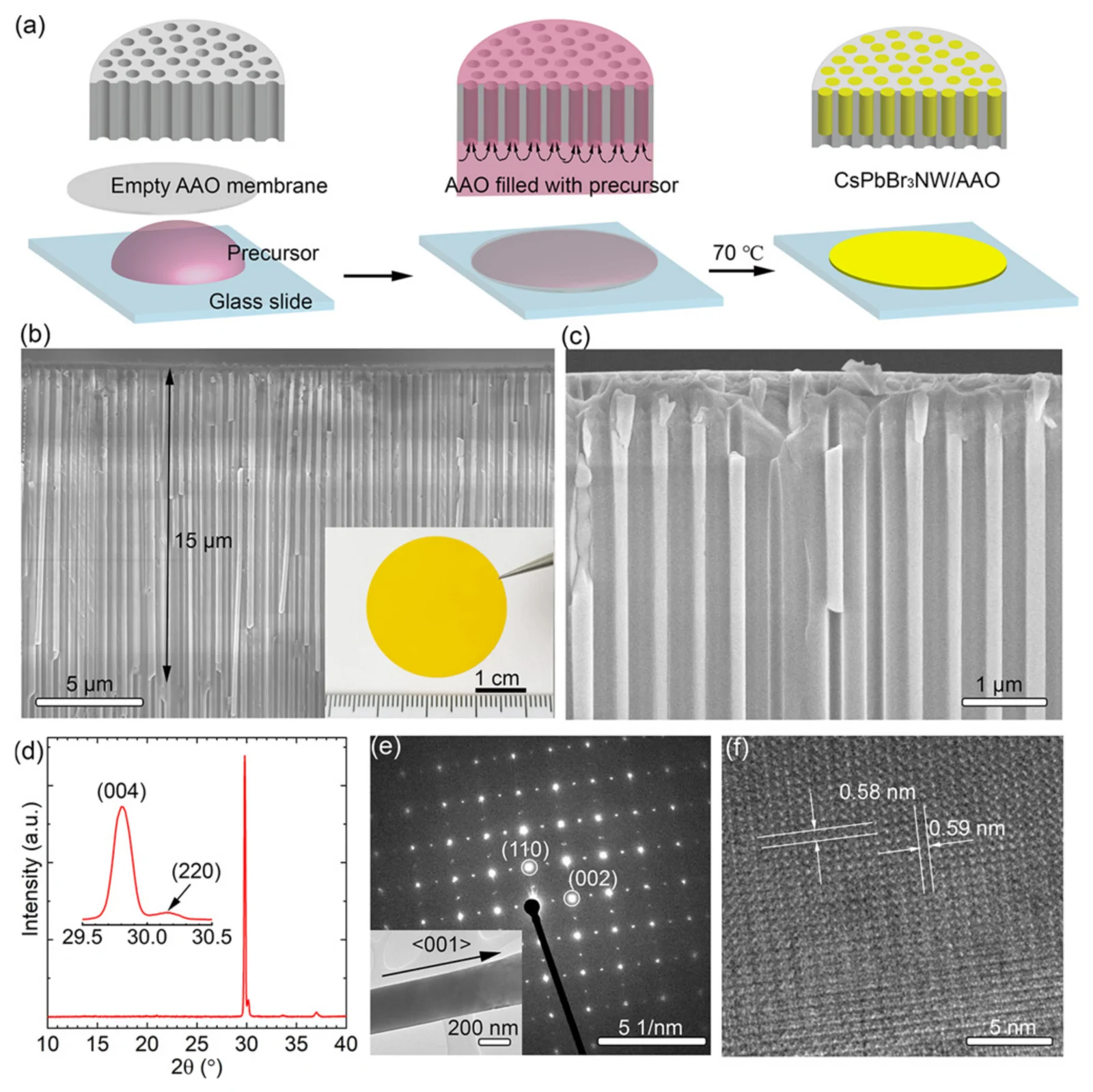
CsPbBr_3_ nanowires grown in AAO membranes. (**a**) Schematic diagram of the growth process of CsPbBr_3_ NWs in AAO membranes. (**b**) Cross-sectional scanning electron microscopy (SEM) image showing a uniform nanowire length of ∼15 μm. The inset shows a photo of the as-grown scintillator sample. (**c**) Higher-magnification SEM showing the nanowires inside the AAO. (**d**) X-ray diffraction (XRD) pattern of the as-grown sample. The inset shows the 2θ range from 29.5° to 30.5°. (**e**,**f**) Transmission electron microscopy (TEM) of a single CsPbBr_3_ nanowire extracted from the cross section of the sample. (**e**) Selected area electron diffraction (SAED) pattern. The inset shows low-magnification TEM. (**f**) High-resolution TEM. Reprinted from Ref. [107].

A scintillator’s spatial resolution is governed by the transparency and thickness of the scintillating film, together with how uniformly the PNCs are distributed within it. Zhang et al. [107] reported a method for growing perovskite nanowires in situ inside anodized aluminum oxide (AAO) membranes, an approach that substantially improved the spatial resolution of the resulting composite scintillators. The nanowires were grown using a one-step, low-temperature solution-growth process carried out within commercial AAO membranes (Figure 3): a precursor mixture of CsBr and PbBr_2_ in DMSO was first deposited onto a glass substrate, an AAO membrane was then placed on top, and the precursor solution infiltrated the membrane’s pores within 1 min via capillary action. The membrane was subsequently transferred to a hot plate held at 70 °C, allowing the nanowires to grow within the pores over 30 min. Varying the amount of precursor solution produced nanowire (NW) arrays ranging from 4 to 12 μm in length. When the precursor mixture was instead deposited on top of an AAO membrane rather than infiltrated into it, only a thin film or polycrystalline perovskite structures formed on the surface.

Transmission electron microscopy and XRD analysis revealed that each nanowire is composed of an array of parallel-aligned CsPbBr_3_ single crystals. Because this parallel arrangement is imposed by the porous AAO template itself, it suppressed light scattering within the scintillator and enabled a spatial resolution fine enough to distinguish parallel lines spaced just 2 μm apart (approximately 160 lp/mm). Using AAO membranes with varying pore sizes, the authors were able to produce nanowire arrays spanning diameters from 30 to 360 nm. Light yield rose as nanowire diameter decreased, peaking at 5300 photons/MeV for the thinnest, 30 nm-diameter nanowires.

However, the AAO matrix itself proved to be a significant absorber of X-ray radiation: equivalent nanowires grown without the AAO template achieved a much higher light yield of approximately 19,000 photons/MeV—on par with pristine CsPbBr_3_ PNC films (21,000 photons/MeV) [108], and two orders of magnitude above that of microcrystalline CsPbBr_3_ films (200 photons/MeV). The nanowire/AAO composites also proved considerably more stable under prolonged irradiation: after 24 h of continuous X-ray exposure in air, the plain CsPbBr_3_ film lost 60% of its scintillation signal, whereas the 30 nm and 170 nm nanowire/AAO samples lost only 20% and 30%, respectively. The 30 nm sample behaved somewhat differently still, first gaining about 10% in intensity over the initial 0.5–1 h before settling into a stable signal.

A related strategy for fabricating AAO-based scintillators was demonstrated by Li et al. [109]. In contrast to the work described above, this study used pre-synthesized CsPbBr_3_ PNCs (approximately 12 nm in size) rather than PNC precursors, loading them directly into the pores of the AAO membrane. By optimizing the membrane’s pore length (20 µm) and diameter (300 nm), the authors achieved a spatial resolution of 250 lp/mm—roughly 1.5 times finer than that reported for the in situ-grown nanowire scintillators discussed previously. The resulting CsPbBr_3_-AAO arrays reached an average light yield of 11,100 photons/MeV, twice that obtained for perovskites grown in situ within AAO, with a scintillation decay time of 9.6 ns for the PNCs in solution. Stability was likewise markedly improved: the scintillator lost only 40% of its photoluminescence after 45 days of storage in open air (relative humidity ≈ 45%), whereas an unprotected PNC film without any protective matrix suffered the same 40% loss in just 7 days.

### 3.2. Doped PNCs in a Glass Matrix

Doping PNCs with rare-earth metals (REMs) offers another route to improving the properties of PNC-based scintillators. REMs act as nucleating agents that promote controlled crystallization [110], improving both crystal growth and the uniformity of nanocrystal distribution within the host matrix. They can also passivate surface defects and suppress non-radiative recombination—thereby raising PLQY [111]—while further enhancing scintillator transparency and increasing scintillator density for more efficient X-ray absorption. In some cases, REMs additionally function as photosensitizers, efficiently transferring absorbed energy to other activator ions [112].

Ma et al., for instance, reported an in situ growth process for CsPbBr_3_ PNCs doped with Eu^3+^ ions [85]. They selected zinc borosilicate glass as the host matrix, combining its components (35% B_2_O_3_, 35% SiO_2_, 12% ZnO) with PNC precursors (9% Cs_2_CO_3_, 6% PbBr_2_, 3% NaBr, and up to 6% Eu_2_O_3_) via mortar homogenization. This mixture was heated to 1200 °C for 20 min in air, then held at 420 °C for 3 h and at 500 °C for a further 17 h to yield the final Eu-doped PNC scintillator. Even a small quantity of Eu^3+^ dopant was enough to optimize PNC crystallization and improve the uniformity of nanocrystal distribution, which in turn reduced light scattering and boosted spatial resolution: samples with 1.5% Eu reached a resolution of 15 lp/mm, versus just 4.1 lp/mm for an undoped equivalent. These scintillators achieved a light yield of 10,100 photons/MeV and a photoluminescence decay time of 6.78 ns, and their radioluminescence remained fully stable—100% retained—after 100 h of X-ray irradiation (189 µGy_air_/s) or heating to 85 °C.

Tong et al. pursued a comparable Eu^3+^-doping strategy [113], synthesizing a series of borosilicate CsPbBr_3_-xEu composites via melt-quenching, with molar proportions of 75% (B_2_O_3_-SiO_2_-ZnO-Al_2_O_3_-MgO)–25% (Cs_2_CO_3_-PbBr_2_-NaBr)–xEu_2_O_3_ (x = 0, 0.6, 1.2, 1.8). Their 0.6%-Eu^3+^ samples proved considerably more resistant to both humidity and radiation than undoped controls: Pb^2+^ leaching stayed largely stable after five days of water immersion (versus an increase from 12.32 to 13.46 µg/L for undoped PNCs), and RL retention after 25 h of X-ray exposure (105 µGy_air_/s) reached 100%, compared to only 84% for undoped samples. The doped composites were also more optically transparent, owing to reduced light scattering. On the other hand, Eu^3+^ doping came with trade-offs: PL decay time lengthened (from 43.95 ns to 49.17 ns), even as RL intensity doubled.

A separate study by Su et al. explored Ce^3+^ doping of CsPbBr_3_ PNCs [114], again using borosilicate glass scintillators prepared by mixing precursors—40SiO_2_−22.5CaO−20B_2_O_3_−17.5Li_2_CO_3_−5PbBr_2_−5CsBr-xCeO_2_-2xSb_2_O_3_ (x = 0, 0.1, 0.2, 0.3, 0.4 mol%)—followed by melt-quenching. Ce^3+^ incorporation raised PLQY from 17% to 28% and boosted RL by 190%, suggesting it can substantially mitigate the poor X-ray energy absorption typical of glass matrices. These scintillators also proved far more durable than pristine PNCs: after 100 days at 90% humidity, their luminescence remained unchanged, whereas a colloidal solution of pristine PNCs lost its luminescent properties entirely under the same conditions. Ce^3+^ doping produced only a modest lengthening of PL decay time, from 19.2 to 21.6 ns.

Niu et al. investigated Gd^3+^ doping of CsPbBr_3_ PNCs and its effect on scintillator efficiency [115], preparing both PNC-containing samples—from a precursor mixture of (35 − X)SiO_2_-28B_2_O_3_-22ZnO-2K_2_O-XGd_2_O_3_-10CsBr-3PbBr_2_ (X = 0, 2, 4, or 6 mol%)—and a PNC-free control (31SiO_2_-28B_2_O_3_-22ZnO-2K_2_O-4Gd_2_O_3_-10CsBr), both via melt-quenching and subsequent thermal treatment. After treatment, the undoped (0% Gd) sample remained as transparent as the starting glass was, while the 2% and 4% Gd samples took on the yellowish-green coloration characteristic of PNC formation; this color intensified with higher temperature or Gd content, and the 6% Gd sample turned opaque due to self-crystallization of the matrix, even though PL intensity remained comparable across the 2%, 4%, and 6% Gd samples. The 4% Gd composition gave the best results overall, with the highest PLQY (37.7%) and a fluorescence decay time of 44 ns—nearly tenfold shorter than that of BGO (~300 ns). These samples also fully recovered their RL properties across 6 cycles of heating to 480 °C, a degree of thermal resilience unattainable in conventional CsPbBr_3_ PNCs, which typically suffer thermal instability at high temperatures due to ligand desorption at the nanocrystal surface. Spatial resolution likewise improved markedly, reaching 12.7 lp/mm for 4% Gd samples versus 4.1 lp/mm for undoped controls.

Beyond these examples, other work has examined how ions such as Mn^2+^ [116] and Zn^2+^/S^2−^ [117] affect the microstructure, optical properties, and formation kinetics of CsPbBr_3_ PNCs in glass matrices—findings that may similarly inform future scintillator design.

### 3.3. In Situ-Grown PNCs and Heterofunctional Nanostructures

Localized surface plasmon resonance, arising when luminescent nanomaterials such as PNCs are located in vicinity to noble metal nanoparticles, offers another route for enhancing their optical output [118]. Building on this principle, Du et al. incorporated silver nanoclusters into CsPbBr_3_ PNC-based scintillators to boost their luminescence [119]. They prepared a borophosphate glass host containing PNCs by melt-quenching a mixture of 15P_2_O_5_-5Na_2_O-5K_2_O-10ZnO-10Al_2_O_3_-40B_2_O_3_-7Cs_2_O-3PbBr_2_-5NaBr (mol%), then polished and cut the resulting glass into scintillator pieces measuring 10 × 10 × 2 mm^3^.

Silver was introduced into the glass via ion exchange, a technique the authors favored for being inexpensive, scalable, and capable of uniformly distributing large quantities of Ag^+^ ions. The glass pieces were embedded in a solid AgNO_3_:NaNO_3_ salt mixture (AgNO_3_ concentrations of 0.25, 0.5, 1.0, 1.5, or 3.0 mol%) and held at 360 °C for 4 h; afterward, they were rinsed with absolute ethanol and deionized water to remove residual nitrate from the surface, then annealed at 430 °C for 3 h to promote formation of both PNCs and silver nanoparticles. XRD and X-ray photoelectron spectroscopy confirmed that both species had formed, while TEM revealed their spatial arrangement relative to one another. Elemental microanalysis further showed that the PNCs were evenly distributed throughout the glass, with Ag^+^ ions penetrating tens of micrometers into the scintillator’s volume.

Because the silver nanoparticles absorbed strongly between 400 and 500 nm, PNC absorption in the neighboring 500–550 nm range increased correspondingly relative to silver-free controls—a signature consistent with either plasmon-driven energy transfer between the two species or partial replacement of Cs^+^ by Ag^+^ within the PNC lattice. Samples containing 1% Ag exhibited the strongest PL response, roughly triple that of silver-free samples, while higher silver loadings caused luminescence to decline, possibly due to suppressed PNC formation or to competing absorption and non-radiative losses at the silver nanoparticles. Consistent with a plasmon-mediated mechanism, PL lifetime also fell steadily with silver content, from 24.9 ns at 0% Ag to 8.99 ns at 3% Ag, pointing to an accelerated radiative recombination rate.

RL tracked the same concentration dependence as PL, again peaking at 1% Ag, where PL rose 3-fold and RL 6.25-fold relative to silver-free samples. Altogether, these findings demonstrate that silver nanoparticle incorporation can simultaneously boost both photo- and radioluminescence intensity while shortening scintillation decay time—a combination of particular value for developing fast, high-efficiency scintillators.

Scintillators in which PNCs are grown within metal–organic frameworks (MOFs) represent a particularly interesting design direction. MOFs are a class of nano/mesoporous materials built from metal ions or clusters (nodes) linked by organic bridging ligands into a three-dimensional crystalline network. Considering scintillators, MOF matrices serve several roles at once: they slow PNC crystal growth through controlled ion diffusion in the pores, preventing agglomeration; they act as a fixed reservoir of metal ions that drives directed PNC formation; they impose quantum confinement via their restricted pore size; and they shield the PNCs from external factors such as moisture, oxygen, heat, and radiation.

**Figure 4:**
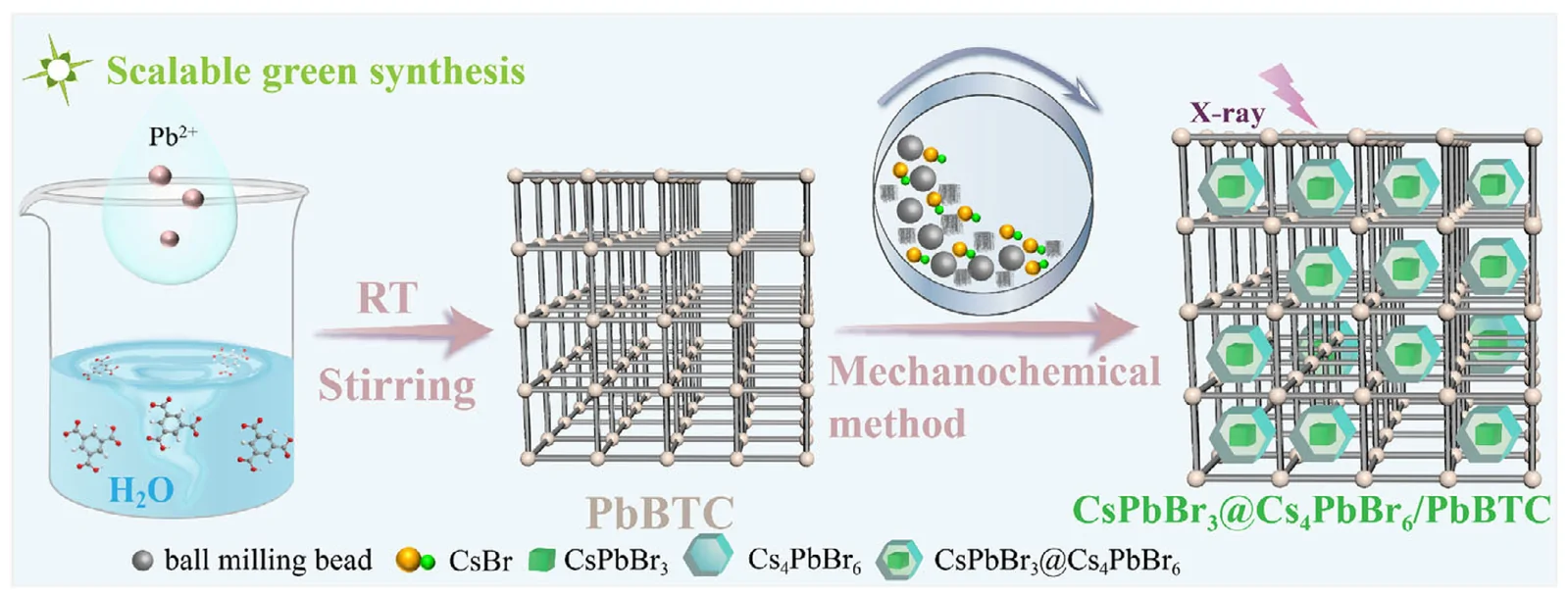
Schematic diagram of scalable green synthesis of CsPbBr_3_@Cs_4_PbBr_6_/PbBTC. RT, room temperature. Reprinted with permission from Ref. [120]. Copyright © 2025 Wiley-VCH GmbH.

Cao et al. put this strategy into practice using PbBTC, a MOF built from Pb^2+^ and 1,3,5-benzenetricarboxylic acid (BTC), in which the organic ligand anchors Pb^2+^ ions within the framework, positioning them as reactive centers for subsequent perovskite formation [120]. Their synthesis unfolds in two room-temperature stages (Figure 4). First, the PbBTC MOF matrix itself is formed by combining 1,3,5-benzenetricarboxylic acid (H3BTC) with lead acetate Pb(Ac)_2_ in water under vigorous stirring for 12 h; the resulting white PbBTC precipitate is then filtered, washed, and air-dried. Second, the PbBTC powder is reacted with CsBr via mechanochemical synthesis (ball milling). Testing several PbBTC:CsBr mass ratios, the authors identified 1:2—denoted (PbBTC)_0.33_(CsBr)_0.67_—as optimal, yielding the composition CsPbBr_3_@Cs_4_PbBr_6_/PbBTC. PbBTC thus plays a dual role here, simultaneously supplying Pb^2+^ ions for PNC formation and acting as a structural template that directs their in situ crystallization.

To fabricate scintillator films, the CsPbBr_3_@Cs_4_PbBr_6_/PbBTC particles were dispersed into a PMMA solution in ethyl acetate, poured onto a glass substrate, and dried at room temperature; a scintillator mass fraction of 50% within the polymer proved optimal. The resulting material forms a triple heterostructure—CsPbBr_3_ nanocrystals (~10 nm, cubic phase) embedded within a Cs_4_PbBr_6_ matrix (0D phase, hexagonal), both housed inside the pores of the PbBTC framework. TEM confirmed that the CsPbBr_3_ nanocrystals are dispersed in the Cs_4_PbBr_6_ matrix while the surrounding PbBTC structure retains its original morphology, consistent with CsPbBr_3_ being doubly encapsulated within Cs_4_PbBr_6_ and PbBTC. Elemental mapping showed uniform distribution of both PbBTC and CsPbBr_3_@Cs_4_PbBr_6_, and confocal imaging of individual particles at varying depths revealed a consistently uniform fluorescence signal—further evidence that CsPbBr_3_@Cs_4_PbBr_6_ forms homogeneously within the PbBTC host.

Because the bandgap of Cs_4_PbBr_6_ (3.63 eV) is much wider than that of CsPbBr_3_, it barely absorbs the photons emitted by the CsPbBr_3_ nanocrystals, effectively acting as an “optically transparent” host that avoids reabsorption losses. PbBTC contributes a further benefit: spatial confinement that raises the exciton binding energy from 39.8 meV (in bulk CsPbBr3) to 146.8 meV—higher than values reported for either colloidal CsPbBr_3_ nanocrystals or CsPbBr_3_@Cs_4_PbBr_6_ lacking the MOF. This elevated binding energy favors efficient exciton recombination and, consequently, efficient conversion of X-ray photons into visible light. Together, these effects give CsPbBr_3_@Cs_4_PbBr_6_/PbBTC a PLQY of ~70.2% and a PL decay time under 20 ns. Its relative light yield reached ~21,760 photons/MeV—256% of the BGO benchmark of 8500 photons/MeV—with a detection limit of 132 nGy_air_/s. Across the 1–103 keV range, its X-ray absorption coefficient surpasses that of both CsI and LuAG, allowing a 200 µm-thick film to attenuate over 95% of 20 keV X-ray photons. Spatial resolution reached 18 lp/mm, well above the 8.9 lp/mm achieved by films based on colloidal CsPbBr_3_ nanocrystals alone.

The MOF architecture also delivered a substantial stability advantage. Under 120 min of UV irradiation (365 nm, 596 mW/cm^2^), CsPbBr_3_@Cs_4_PbBr_6_/PbBTC held onto 90% of its initial PL intensity, versus just 30% for the PbBTC-free CsPbBr_3_@Cs_4_PbBr_6_ composite—while bare CsPbBr_3_ nanocrystals degraded almost immediately. Heating demonstrated a similar trend: the PbBTC-containing composite retained 86% and 65% of its PL at 60 °C and 80 °C, respectively, and bounced back to 85% of its original PL after cooling, compared with only 10% recovery for the PbBTC-free version. Under 24 h of combined 85% humidity and 85 °C heat, the MOF-protected composite kept over 80% of its RL intensity, while the unprotected version fell to 20% or below. Finally, under sustained X-ray irradiation (22 keV, 1 mGy_air_/s) for 2 h—a cumulative dose of roughly 7.2 Gy_air_—the composite retained 88% of its RL.

Gu et al. designed a CsPbBr_3_ nanocrystal/metal–organic framework nanostructure scintillator to resolve two competing requirements: a high PNC loading is needed for sufficient X-ray attenuation, yet concentrated PNC solids tend to aggregate and lose luminescence efficiency [42]. The authors compared two pore architectures built on the same luminescent terbium-based host (Tb-BTC MOF (BTC = benzene-1,3,5-tricarboxylate linker), whose Tb^3+^ centers are themselves scintillating via triplet-state energy transfer from the H_3_BTC linker): a conventional microporous MOF (MP-MOF, pore size ~3 nm) and a hierarchically porous MOF (HP-MOF) obtained by partial hydrolysis of MP-MOF, which introduces linear cracks and macropores of ~100 nm while retaining the original micropores. CsPbBr_3_ PNC were grown in situ within both hosts: Pb^2+^ ions were first adsorbed onto the pore surfaces via Pb–COOH coordination (confirmed by an XPS peak shift), after which a cesium precursor was injected to nucleate and grow the perovskite; the growth cycle was repeated three times to progressively fill the pore volume. Because the macropores of HP-MOF can accommodate far more PNCs than the nanometer-scale micropores of MP-MOF, this procedure achieved a PNC loading ratio of up to 75 wt% for CsPbBr_3_/HP-MOF, versus only 7% for CsPbBr_3_/MP-MOF, as quantified by thermogravimetric analysis and confirmed by scanning electron microscopy and energy-dispersive spectroscopy elemental mapping. The authors show that loading ratio, not the total amount of PNCs applied, is the parameter that determines scintillator performance: for a film with only 5% PNC loading, X-ray attenuation efficiency reaches just 15% even though UV attenuation is already ~100%, whereas a loading of at least 50% is required to exceed 80% X-ray attenuation efficiency. Consistent with this, CsPbBr_3_/MP-MOF (7% loading) shows an almost absent CsPbBr_3_ RL peak under X-ray excitation despite having normal PL under UV excitation, while CsPbBr_3_/HP-MOF shows strong CsPbBr_3_ emission under both, and RL intensity increases linearly with loading ratio across the 13–75% range tested. Beyond simply enabling higher loading, the HP-MOF host also acts as a physical barrier that keeps the densely loaded PNCs well dispersed rather than aggregated. SEM imaging shows CsPbBr_3_ PNCs are not aggregated on HP-MOF surfaces, versus severe aggregation for neat CsPbBr_3_ films, and this is corroborated by higher PLQY (92% for CsPbBr_3_/HP-MOF versus 87% for neat CsPbBr_3_ film), and a longer PL lifetime (55.2 ns versus 43.7 ns) which is consistent with reduced aggregation-induced quenching. Fabricated as a free-standing CsPbBr_3_/HP-MOF-PS composite plate, the CsPbBr_3_/HP-MOF scintillator reached a light yield of 24,800 photons/MeV—2.3 times higher than a neat CsPbBr_3_ PNC scintillator (11,000 photons/MeV) prepared and measured the same way, and also higher than an HP-MOF-only (Tb-MOF, no PNC) reference (14,800 photons/MeV). The authors attribute 66% of this enhancement to the improved luminance efficiency of the less-aggregated, well-dispersed PNCs, and the remaining 34% to additional emission from the Tb-MOF host itself (peaks at 545, 583, and 620 nm); within the composite, CsPbBr_3_ and Tb-MOF contribute 52% and 48% of the total RL, respectively—confirming that the host is not merely a passive scaffold but an active co-emitter. Spatial resolution improved from 14 lp/mm (neat CsPbBr_3_) to 20 lp/mm (CsPbBr_3_/HP-MOF) as a direct result of the reduced light scattering from better-dispersed nanocrystals, and RL intensity remained stable to within 5% under continuous X-ray irradiation, with the organic Tb-MOF component identified as the source of what little degradation did occur. On the other hand, the hydrophilic carboxylate groups of the Tb-BTC linker limit the scintillator’s humidity tolerance. In addition, because the RL decay of CsPbBr_3_/HP-MOF (10 μs) is governed by the comparatively slow Tb^3+^ emission rather than by CsPbBr_3_ itself, the scintillator is better suited to applications with millisecond-scale exposure times than to the fastest imaging modes. The authors demonstrated both static X-ray imaging of integrated-circuit chips (resolving a 70 μm conductor line width in a microSD card) and dynamic imaging of a rotating optical chopper at angular velocities up to 2.7 revolutions per second.

Across the studies discussed in this section, in situ synthesis is shown to trade some control over PNC quality for the practical benefits of a single-step, matrix-integrated fabrication process. Polymer hosts (PMMA, PET, nylon, PVA) dominate the work on pristine PNCs and reliably yield flexible scintillators, whereas glass and other inorganic hosts sacrifice flexibility for markedly higher thermal, chemical, and photostability—the highest reported photoluminescence quantum yields and the most demanding stability tests in this section both come from PNC grown within a silica or glass-type host. Templating PNC growth inside a porous alumina membrane is the only strategy that pushes spatial resolution well beyond what bulk matrix-grown scintillators achieve, pointing to nanocrystal confinement and geometry, rather than matrix chemistry, as the main lever for improving imaging resolution.

The reported performance parameters for these in situ-grown scintillators are summarized in Table 4. Light yield ranges from about 1200 to 55,500 photons/MeV, and the best detection sensitivity reported, 120 nGy/s, was achieved with the simplest system in the table—pristine CsPbBr_3_ in a flexible PMMA matrix—indicating that high sensitivity does not necessarily require a more elaborate host or dopant strategy. Spatial resolution separates into two regimes: about 8–25 lp/mm for scintillators in which PNCs are grown throughout a bulk polymer or glass matrix, versus 160–250 lp/mm for the two AAO-templated studies, the higher of which in fact used pre-synthesized rather than in situ-grown PNCs as a comparison sample.

## 4. Scintillators Made with Pre-Synthesized PNCs

Beyond in situ growth, PNCs can be pre-synthesized—in solution or as a solid, typically via hot-injection, ligand-assisted reprecipitation, or ball milling—and only combined with a supporting matrix afterward, or in some cases form a single-component scintillator. Decoupling PNC synthesis from matrix formation offers a key advantage: it allows the nanocrystals to be produced under fully controlled conditions and characterized both before and after incorporation, making it possible to isolate the matrix’s specific effect on the composite properties. The trade-off is that surface ligands stabilizing the PNCs can desorb during storage or incorporation into the matrix, introducing a degradation pathway that in situ growth largely overcomes. The following subsections survey the main strategies for building PNC-based scintillators: embedding pristine or doped PNCs within a matrix, building hybrid systems, and, finally, dispensing the nanocrystals themselves to form the scintillating film.

### 4.1. Pristine PNCs in a Matrix

The most common approach to fabricating scintillators from pre-synthesized PNCs is their incorporation into a polymer matrix. The matrix serves several purposes simultaneously: it physically isolates the nanocrystals from moisture and oxygen, prevents their aggregation, and imparts mechanical strength and flexibility to the composite. However, incorporation into a polymer inevitably affects the properties of the PNCs themselves—polar groups within the matrix (e.g., the acrylate groups of PMMA) can quench the luminescence of high-quality nanocrystals, while conversely passivating surface defects in lower-quality ones. Key parameters governing this trade-off include PNC loading (higher loading enhances X-ray absorption but reduces transparency and increases self-absorption and scattering), the nature of the surface ligands (which determine matrix compatibility, quantum yield, and thermal stability), and film thickness (a balance between absorption efficiency and spatial resolution). The studies discussed in this subsection trace how sequential optimization of PNC composition, matrix selection, and ligand engineering has allowed the characteristics of composite scintillators to approach—and in some cases surpass—those of commercial analogs.

#### 4.1.1. Effect of Halide Composition of PNCs on Their Radioluminescence

The fabrication of scintillators from pre-synthesized PNCs enables detailed investigation of how PNC composition affects scintillator parameters such as brightness and RL decay time. O’Neill et al. investigated the effect of PNC composition (CsPbCl_x_Br_1−x_) on RL light yield and decay response, alongside X-ray sensitivity [121]. They used commercially available PNCs with compositions CsPbCl_1.5_Br_1.5_ (emission 450 ± 2 nm, size 8 ± 1 nm), CsPbClBr_2_ (emission 475 ± 2 nm, size 9 ± 1 nm), and CsPbBr_3_ (emission 512 ± 2 nm, size 10 ± 1 nm), with chemical composition confirmed by energy-dispersive X-ray spectroscopy (EDX). Scintillators were fabricated by loading PNCs of each composition at 2 wt% into a PMMA solution in toluene, mixing at 56 °C for 2 h, and then drying in casting molds for 2 days. All three scintillator samples showed a peak emission wavelength approximately 6 nm higher than their respective solutions, indicating some degree of nanoparticle agglomeration following encapsulation in PMMA. The RL lifetime was roughly three times longer than the PL lifetime in each case (30.6 vs. 11.4 ns for CsPbCl_1.5_Br_1.5_, 33.9 vs. 12.2 ns for CsPbClBr_2_, and 27.7 vs. 9.3 ns for CsPbBr_3_). Notably, CsPbBr_3_ also showed the highest RL brightness: taking the sensitivity of CsPbCl1.5Br1.5 as a 100% baseline, CsPbClBr_2_ reached 350% and CsPbBr_3_ reached 413%. The authors conclude that chlorine content in the PNCs reduces both X-ray sensitivity and emission intensity, and accordingly recommend CsPbBr_3_ for fabricating nanocomposite scintillators for X-ray detectors.

Chen et al. similarly compared the scintillation properties of inorganic PNCs across a range of compositions [122]. They synthesized CsPbX_3_ (X = Cl, Br, or I) PNCs by controlling the reaction of Cs-oleate with various PbX_2_ precursors via a hot-injection method [123], producing PNCs with compositions CsPbCl_3_, CsPbCl_2_Br, CsPbCl_1.5_Br_1.5_, CsPbClBr_2_, CsPbCl_2.5_Br_0.5_, CsPbBr_3_, CsPbBr_2_I, CsPbBr_1.8_I_1.2_, CsPbBr_1.5_I_1.5_, CsPbBr_1.2_I_1.8_, CsPbBrI_2_, and CsPbI_3_, with an average size of approximately 9.6 nm. These PNCs exhibited distinct luminescence colors under X-ray irradiation depending on composition, enabling both color-tunable scintillators and their potential use as fluorescent markers for multiplexed detection. Flexible scintillators based on these PNCs were fabricated either by combining solution processing with soft lithography, or by directly casting the oleate-capped PNCs onto a flexible substrate. Under irradiation at a source power of 5 µGy/s, a 0.1 mm-thick CsPbBr_3_-based scintillator embedded in a PDMS matrix achieved a light yield comparable to that of a CsI:Tl scintillator roughly 5 mm thick, with a detection limit of only about 13 nGy/s. These scintillators also showed an RL decay time of just 44.6 ns—substantially shorter than the ~1000 ns decay time of CsI:Tl. This work also proposes a plausible mechanism for PNCs radioluminescence. Incident X-ray photons (below a few hundred keV) interact with the perovskite lattice mainly via the photoelectric effect, generating large numbers of hot electrons and holes that migrate between neighboring nanocrystals before thermalizing to the band edges—a process confirmed experimentally through X-ray-induced photocurrent measurements. These carriers subsequently trap and recombine radiatively, with emission color tunable via the bandgap engineering. The resulting high scintillation intensity is attributed to the strong X-ray stopping power and quantum confinement effects characteristic of PNCs. Finally, although the authors do not explicitly discuss the effect of heavy iodine atoms on the RL properties of PNCs, their choice of CsPbBr_3_ as the base material for the composite scintillators may hint at lower RL efficiency in iodine-containing PNCs, or at underlying environmental stability concerns with that composition.

The synthesized PNCs of various colors were further coated with a silica shell to prevent degradation upon contact with biological fluids [124], enabling their use in in vivo multiplexed bioimaging following subcutaneous injection in mice. Notably, the past decade has seen substantial growth in the use of PNCs for bioimaging applications—not only within X-ray detectors, but also as probes for analyte detection [125], since PNCs, like other fluorescent nanocrystals, can be conjugated with detection molecules [126].

#### 4.1.2. Selection of Polymer Matrix and Optimal PNC Loading

One of the simplest ways to incorporate PNCs into a polymer matrix is solution mixing. Wang et al. fabricated flexible scintillators based on CsPbBr_3_ PNCs with areas of up to 100 cm^2^ [127]. PNCs synthesized via the hot-injection method were mixed with a PMMA solution in toluene and poured into a pre-prepared mold, with film thickness controlled both by varying the PMMA concentration and by adjusting the PMMA:PNC ratio in the mixture at a given concentration. After toluene evaporation and PMMA polymerization, the resulting scintillation film (average surface roughness 73 nm) was separated from the substrate. The spatial resolution of these scintillators decreased with increasing thickness (0.01, 0.04, 0.09, and 0.15 mm), yielding values of 17.3, 8.0, 6.2, and 5.9 lp/mm, respectively—a decline attributed to greater X-ray scattering in thicker films. Maximum RL intensity and lowest detection sensitivity occurred at a thickness of 0.04 mm, while the highest detection sensitivity and lowest RL intensity occurred at 0.01 mm. Radiation stability, however, was largely independent of thickness, decreasing by no more than a few percent over 4 days of irradiation at 5.5 µGy_air_/s. These films also retained more than 80% of their PL intensity after over three months of water immersion.

Skrypnyk et al. likewise studied CsPbBr_3_ PNC-based scintillators encapsulated in a PMMA matrix [128]. PNCs, with an average size of 20 nm, were synthesized by ligand-assisted reprecipitation using n-octylamine and oleic acid as surface ligands. Scintillation films were prepared by combining a PNC solution in toluene with a PMMA solution in chloroform and casting the mixture into molds, yielding scintillators 70, 140, and 200 µm thick, each containing 2 wt% PNCs. Embedding PNCs in PMMA left the fluorescence peak position unchanged, pointing to minimal aggregation, while the PL lifetime dropped sharply—from 27 ns in solution to just 2 ns within the matrix—largely because the matrix suppressed the slow, microsecond-scale decay component otherwise present in solution. The resulting scintillators showed an RL lifetime of only 1.4 ns and, for the 200 µm-thick sample, a light yield of 860 photons/MeV.

Unusually for this line of research, the authors examined how these PNC scintillators respond to alpha radiation. They found that thicker films absorb more energy from incoming α-particles and therefore generate more scintillation photons, translating into higher count rates, greater sensitivity, and a lower minimum detectable activity. Even so, at 2 wt% PNC loading, a thickness of 200 µm still fell short of producing a high light yield, suggesting that further gains in performance will require increasing both PNC concentration and film thickness beyond the ranges tested here.

Another approach to fabricating composite PNC-based scintillators is radical polymerization, in which pre-synthesized PNCs are dissolved directly in the monomer before polymerization is initiated thermally or photochemically. Heo et al. compared a PNC-based scintillation film with a commercial terbium-doped gadolinium oxysulfide (GOS) scintillator [129]. Cubic CsPbBr_3_ PNCs with an average size of 9 nm were synthesized via hot-injection and then encapsulated in PMMA through photoinitiated radical polymerization, using 1 mL of PNC solution (0.1 g/mL) per 5 mL of methyl methacrylate, and deposited onto a glass substrate. Unlike the GOS scintillator, which shows multiple fluorescence peaks at different wavelengths, the PNC-based scintillator exhibited a single peak near 550 nm, with a PL lifetime of approximately 2.87 ns—5.6 times shorter than that of GOS (15.99 ns). The PNC-based scintillator also showed higher X-ray absorption and conversion efficiency than GOS across dose rates up to 500 µGy/s. Using a CMOS sensor with a 20 µm pixel size, the authors measured a spatial resolution of 9.8 lp/mm for the PNC-based scintillator, compared with only 6.2 lp/mm for GOS. The CsPbBr_3_ PNC scintillator also lost only 4% of its initial signal after a cumulative dose of 40 Gy_air_, a stability comparable to that of GOS.

Cova et al. examined how incorporating CsPbBr_3_ PNCs into either PMMA or polystyrene (PS) matrices affects their scintillation and optical properties [44]. Using a modified ligand-assisted reprecipitation method [130], they synthesized both high-quality PNCs and PNCs with deliberately introduced surface defects—the latter obtained by excluding oleic acid from the reaction, lowering the reaction temperature, and modifying the precursor-mixing procedure. Following synthesis and purification, the PNCs were incorporated into either the PMMA or PS matrix. The absorption edge and PL peak were identical for PNCs in toluene and in PMMA, indicating that the electronic structure and nanocrystal size are preserved upon incorporation. However, PLQY dropped from 34 ± 5% (in toluene) to 17 ± 5% (in PMMA), a result of static quenching of a fraction of the PNCs by PMMA’s polar acrylate groups. For the defective PNCs, the trend reversed: PLQY rose from 2 ± 1% to 20 ± 5%, indicating that additional surface-defect passivation by the matrix outweighed host-induced fluorescence quenching in this case. Incorporation of PNCs into PMMA also slowed the fast PL decay component of the former and reduced the input of the delayed PL component from 50% to 25%; by contrast, incorporation of PNCs into PS (which lacks acrylate groups) left the PL kinetics essentially identical to those in solution, confirming that this effect originates specifically from PMMA’s acrylate groups. These results suggest that the balance between matrix-induced degradation and defect passivation depends on the initial density of surface defects: PNCs with high defect densities benefit from incorporation into a polar (acrylate) matrix, whereas high-quality PNCs are better served by nonpolar matrices such as PS. Radiation-stability studies further showed that both pristine PNCs in toluene and PNCs embedded in PMMA maintained stable RL up to accumulated doses of 1 MGy, whereas conventional plastic scintillators typically lose 40–80% of their light yield at comparable doses. Time-resolved RL measurements of CsPbBr_3_ PNCs in PMMA revealed ultrafast kinetics (RL lifetime = 1.81 ns), comprising a prompt decay component (~75 ps, limited by instrument response), a ~600 ps component attributed to multi- and charged-exciton recombination, and a ~7 ns tail arising from neutral band-edge excitons.

Li et al. fabricated scintillators based on PNCs encapsulated in an ethylene vinyl acetate copolymer (EVA) [131]. The CsPbBr_3_ PNCs were synthesized via a hot-injection method using n-octanoic acid and n-octylamine, and the resulting scintillators were fabricated by incorporating 1.25–5% PNCs into an EVA solution in toluene heated to 90 °C. TEM analysis showed an average nanocrystal size of approximately 15 nm, PNCs had a PLQY reaching 90% and an average PL lifetime of approximately 14 ns. EVA was selected as the encapsulating matrix for its water resistance, transparency, flexibility, ease of preparation, and low cost. It serves a dual role as both surface-capping ligand and matrix, protecting the CsPbBr_3_ nanocrystals from the water and oxygen exposure that typically degrade PNC fluorescence. As a result, EVA fully preserved the RL properties of the PNCs during 20 min of X-ray irradiation and 10 h of immersion in water. Increasing PNC loading from 1.25% to 5% improved X-ray image contrast, with the 5% film clearly revealing the internal circuitry of a test chip; however, higher loading also reduced light transmittance.

Erroi et al. fabricated a scintillator based on CsPbBr_3_ PNCs in a methyl methacrylate/lauryl methacrylate (MMA/LMA, 80:20 wt%) matrix [69]. The PNCs were synthesized by mixing solutions of PbBr_2_ and Cs_2_CO_3_ in isopropanol with tetrabutylammonium bromide (TBAB), oleylamine, and propionic acid under turbo-emulsifier homogenization, yielding rectangular nanocrystals with an average size of 13 ± 3 nm and thickness about 5 ± 1 nm. After synthesis and purification, these PNCs were incorporated into the MMA/LMA matrix at weight concentrations of 0.05–0.8% by radical polymerization using photoinitiation. LMA was included in the matrix because its long alkyl side chains create a near-native, nonpolar polymeric microenvironment for the PNCs, improving their compatibility with the host matrix and preserving their optical properties.

During acrylate polymerization, the absorption spectrum evolved toward the profile typical of CsPbBr_3_ PNCs, developing a first excitonic peak at 2.4 eV, consistent with the average particle thickness of 9 ± 2 nm measured by TEM. An isosbestic point at 2.45 eV confirmed the conversion of the initially heterogeneous native particles into a homogeneous PNC ensemble. This structural evolution markedly improved both spectral purity and emission efficiency: PLQY increased from 43 ± 5% to 90 ± 5%, and the resulting nanocomposites exhibited narrow photoluminescence (FWHM = 17 nm, 78 meV) with a uniform lifetime of about 11 ns across the entire spectrum—roughly twice as long as any individual emission component of the native PNCs.

The maximum light yield, 4800 photons/MeV, was achieved at the highest PNC loading tested (0.8%). These scintillators also exhibited exceptional radiation stability, retaining stable fluorescence up to an accumulated dose of 1 MGy. The authors suggest this stability may stem from the self-healing capacity of lead halide perovskites following structural damage, such as vacancy formation or surface segregation of metallic lead [132]. To characterize time resolution of the composite scintillator, the authors used time-correlated single photon counting. The effective RL decay time increased with PNC content, measuring 1.13, 1.76, 1.54, 3.3, and 4.1 ns for scintillators with PNC concentrations of 0.05%, 0.1%, 0.2%, 0.4%, and 0.8%, respectively. The lowest-PNC-content scintillator achieved a particularly fast response, with more than 30% of scintillation occurring within the first 80 ps.

One of the main challenges in incorporating PNCs into solid matrices for scintillator fabrication is the typically low PNC content achievable in the composite, along with non-uniform distribution of the nanocrystals within it. To address this problem, Bellotti et al. employed a chain transfer agent that could interact with the PNC surface during CsPbBr_3_-initiated PET-RAFT (photoinduced electron transfer–reversible addition-fragmentation chain transfer) polymerization of methyl methacrylate [133]. The PNCs were synthesized by mixing two precursor solutions—one containing Cs_2_CO_3_ and propionic acid in a heptane/isopropanol mixture, the other containing PbBr_2_ and tetrabutylammonium bromide in a mixture of oleylamine, propionic acid, and isopropanol—using a turbo-emulsifier. Addition of an antisolvent (isopropanol) then triggered precipitation of the PNCs, which were collected by centrifugation. The resulting PNCs had an average size of approximately 13 nm and a photoluminescence quantum yield of 64%.

To form the matrix and incorporate the PNCs into it, the polymerization agent (2-cyanoprop-2-yl dithiobenzoate), MMA, and the synthesized PNCs were combined in toluene and deoxygenated by nitrogen bubbling. Polymerization was then carried out by irradiating the mixture with UV light for 24 h, achieving up to 89% MMA monomer conversion and incorporation of up to 7% PNCs into the matrix. TEM analysis of different sections of the resulting scintillator, imaged at various depths, confirmed improved PNC dispersibility, supporting the proposed mechanism whereby the chain transfer agent interacts with the PNC surface as polymerization proceeds.

The light yield of a 7 mm-thick scintillator containing 7% PNCs reached 6000 photons/MeV—close to the 8600 photons/MeV achieved by the commercial EJ276D scintillator. For scintillators with 7% PNC content, the effective fluorescence lifetime was approximately 490 ps when excited by a radiation source with an energy of 511 keV.

An interesting example of fabricating 3D scintillators using a UV-curable acrylic-based resin known as “GENESIS-Development Resin Base” (GDRB) is described by Giuri et al. [134]. A distinctive feature of this polymer is that it can be mixed with solid powders to create novel, printable composites, enabling fabrication of composites with high filler loading. For this purpose, the authors used 0D Cs_4_PbBr_6_ perovskites synthesized by antisolvent precipitation, using CsBr and PbBr_2_ as precursors and a mixture of 3% water in tetrahydrofuran as the antisolvent.

Flat, thin-layer scintillators were fabricated by blending 0D Cs_4_PbBr_6_ perovskites into GDRB at mass concentrations from 0.25% to 30%, applying the mixture to an aluminum substrate via a doctor-blade method to achieve thicknesses of 150–300 µm, then photopolymerizing the composite under UV light in a nitrogen atmosphere for 2 h. Stereolithography was also used to 3D-print models, building up the composite containing 0.25% Cs_4_PbBr_6_ in 0.05 mm layers. SEM imaging showed that the Cs_4_PbBr_6_ particles—with sizes on the order of hundreds of nanometers—remained uniformly distributed within the resin even at the highest loading tested (30%). XRD analysis further showed that the GDRB matrix reliably protected the perovskites from atmospheric moisture for at least 13 days.

As a result, incorporating Cs_4_PbBr_6_ into the GDRB matrix preserved the material’s ultrafast scintillation response, with an effective decay time of approximately 1.0 ns, and achieved a light yield of approximately 10% that of the commercial EJ232 scintillator.

#### 4.1.3. Surface and Ligand Engineering

Among the many strategies for enhancing PNC stability, encapsulation in solid matrices—inorganic oxides (SiO_2_, Al_2_O_3_) and organic polymers (PS, PMMA)—occupies a special place, since it provides physical isolation of the nanocrystals from the environment. Chen et al. proposed a hybrid organosilicon-based ligand approach combining the advantages of both oxide and polymer encapsulation [43]. Traditional long-chain organic ligands, such as oleic acid (OA) and oleylamine (OAm), are commonly used to stabilize colloidal PNCs during synthesis, but are incompatible with the cross-linking chemistry of organosilicon matrices; using them as capping ligands in this context leads to low transparency and high surface roughness in the resulting composite.

To address this issue, the authors developed a dual organosilicon ligand system, replacing the classical OA/OAm ligand pair with (3-aminopropyl)triethoxysilane (APTES) ligands bearing an amino headgroup and (3-aminopropyl)triethoxysilane with pentanedioic anhydride (APTES-PA) ligands bearing a carboxyl headgroup. APTES alone provides sufficient surface passivation for the PNCs but cannot maintain colloidal stability of the dispersion; the longer-chain APTES-PA ligand supplies this stability in low-polarity solvents. Because APTES-PA is shorter than oleic acid and carries alkoxysilane functional groups, it can also participate in the polycondensation reactions of silicone resins, forming a densely cross-linked matrix while simultaneously stabilizing the colloidal PNCs during cross-linking.

In a typical synthesis of APTES-PA-capped CsPbBr_3_ PNCs, PbBr_2_ and CsBr were dissolved in DMF with stirring, followed by addition of the APTES and APTES-PA ligands. This precursor solution was then injected into a 1:1 methylene chloride/toluene mixture under vigorous stirring, producing a bright green colloidal PNC dispersion. The dispersion was drop-cast onto glass slides and dried in a vacuum oven at 65 °C, forming a solid film with a PNC concentration of approximately 36 wt%. As a comparison sample, APTES/OA-capped PNCs were prepared by substituting an equivalent amount of OA for APTES-PA. The resulting PNCs had a cubic structure with a particle size of ~11 nm and were homogeneously distributed within the organosilicon matrix without agglomeration. For further comparison, scintillation films based on PS and oleic acid-capped PNCs (APTES/OA) were prepared by dissolving PS in toluene, adding the PNCs, and drying the resulting solution on a glass substrate.

The optical properties of films made with the new APTES/APTES-PA ligand system significantly outperformed those of the conventional APTES/OA controls: PLQY reached 80.6% for APTES/APTES-PA, compared with only 71.7% for APTES/OA. PL lifetime followed a similar trend—22.7 ns for APTES/APTES-PA, 20.0 ns for APTES/OA, and 17.7 ns for the original OAm/OA pair—consistent, together with the higher PLQY, with more effective passivation of surface defects in the new ligand system.

Stability testing of PNC/APTES/APTES-PA films under various harsh conditions, benchmarked against PNC/PS and PNC/APTES/OA controls, further supported this conclusion. Upon heating to 180 °C, the APTES/APTES-PA film retained 62.3% of its PL intensity, compared with only 36.8% for PNC/PS and 55.2% for PNC/APTES/OA; above 110 °C, the PNC/PS film also developed morphological defects (air bubbles) due to polystyrene’s low glass transition temperature. Under UV irradiation (365 nm, 1.5 mW/cm^2^) for 6 h, the APTES/APTES-PA film retained 78.9% of its PL, versus only 34.1% for PNC/PS. Upon 6 h of water immersion, PL intensity in the APTES/APTES-PA film remained stable, whereas PNC/PS dropped to 71.7%, likely due to incomplete polymer encapsulation at high PNC loading. Across all organic solvents tested (24 h immersion), the APTES/APTES-PA films showed no PL decrease at all, confirming reliable protection of the nanocrystals by the organosilicon matrix. Finally, the 25 µm-thick PNC/APTES/APTES-PA scintillation film achieved a spatial resolution of 20 lp/mm, and its RL remained stable under 180 min of continuous X-ray irradiation at a dose rate of 6.58 mGy/s.

Polystyrene is one of the most widely used scintillator matrices owing to its high radiation resistance; however, since thermally initiated bulk polymerization of styrene requires temperatures above 60 °C, this process can irreversibly damage PNCs. Král et al. presented a surface passivation strategy for CsPbBr_3_ PNCs based on functionalization with ammonium hexafluorosilicate (AHFS) combined with treatment with allyl-terminated alcoxysilane [135]. When used to fabricate scintillating nanocomposites, this strategy improves thermal stability of the material up to 110 °C while simultaneously enhancing nanocrystal compatibility with the matrix.

CsPbBr_3_ PNCs were synthesized by the hot-injection method: cesium oleate was injected into a degassed mixture of PbBr_2_, octadecene, oleic acid, and oleylamine at 170 °C, reacted for 10 s, then quenched in an ice bath. The product was isolated by centrifugation and redispersed in toluene. Ligand exchange was carried out using an AHFS solution in water (2 M) at a ratio of 0.6:1 mg AHFS:PNC, the latter dissolved in toluene. After centrifugation, the isolated PNCs were found to be saturated with surface SiF_6_^2−^ and NH_4_^+^ ions, as confirmed by FTIR spectroscopy. Scanning transmission electron microscopy (STEM) showed no formation of a thick SiO_2_ shell or large silica aggregates, indicating that the surface layer is monomolecular. Following ligand exchange, PLQY increased from 36% (for the original OA/OAm-capped PNCs) to 95%, since the AHFS molecule can fill surface halide vacancies and thereby suppress carrier trapping.

The PNCs were then further reacted with allyltrimethoxysilane (ATMOS), which served two purposes: introducing a thin protective silica layer and functionalizing the PNC surface with vinyl groups capable of copolymerizing with styrene. This step reduced PLQY to 60%, possibly due to the influence of methanol released during ATMOS hydrolysis. For fabrication of PS-based nanocomposites, the AHFS/allyl-functionalized PNCs were dispersed in styrene, an initiator was added, and thermal polymerization was carried out under one of three conditions: 80 °C (3 h), 110 °C (45 min), or 25 °C (14 days).

Allyl functionalization significantly improved the transparency of the resulting scintillation films. At 1 wt% PNC loading and a film thickness of 250 µm, transparency at 525 nm doubled, from 5.7% to 11.4%; at 500 µm thickness, it likewise increased from 1.9% to 6.4%. This improvement is attributed to better PNC distribution within the matrix, achieved through copolymerization of the allyl groups with styrene. Notably, the position and shape of the composites’ emission spectra remained unchanged as the polymerization temperature was increased from 25 °C to 110 °C, indicating that the AHFS/allyl-passivated ligand shell remains stable even under high-temperature polymerization.

To increase the efficiency of ionizing radiation absorption, it is necessary to increase the concentration of PNCs within scintillators. However, this typically reduces transparency and increases self-absorption of the emitted visible light, partly as a result of PNC aggregation. One approach to mitigating this aggregation is passivation with different surface ligands. Decka et al. compared the effect of two different surface ligands—oleic acid/oleylamine (OA + OAm) and didodecyldimethylammonium bromide (DDAB)—on the optical quality and radioluminescence of scintillators fabricated at three different PNC loadings, up to 10%, in a polystyrene matrix [136].

CsPbBr_3_ PNCs were synthesized via the hot-injection method from 0.276 g PbBr_2_, 20 mL 1-octadecene (ODE), 2 mL OAm, 1.78 mL OA, and 0.5 mL of 0.4 M cesium oleate solution. DDAB-capped PNCs were obtained through a subsequent ligand-exchange procedure: the crude reaction mixture in ODE was combined with a 55 mM DDAB solution in toluene (volume ratio 3:2), stirred vigorously, and the resulting nanocrystals were precipitated by addition of ethyl acetate and isolated by centrifugation. Scintillators were then fabricated by mixing a given amount of PS and PNCs in toluene and drying the resulting viscous composite in air.

At 1% PNC loading, scintillators based on OA/OAm-capped PNCs showed 50% transmittance at 800 nm, compared with 56% for DDAB-capped PNCs, a gap that widened further at higher loading levels. The authors attribute this improved transparency in the DDAB system to more effective surface passivation of the nanocrystals. Increasing PNC loading from 5% to 10% raised RL intensity 2-fold for the OA + OAm system, but 2.5-fold for the DDAB system—a difference the authors attribute to DDAB’s ability to more effectively suppress PNC aggregation during composite fabrication. This interpretation is supported by RL decay behavior: no gradual slowing of RL decay was observed with increasing PNC loading for the DDAB system, in contrast to the OA + OAm system, where such slowing did occur. This slowing in the OA + OAm system was itself linked to aggregation, as evidenced by a red-shift in the RL spectra with increasing PNC concentration.

Consistent with this picture, the RL lifetime for DDAB-capped PNC samples showed relatively little dependence on PNC concentration (2.9, 2.4, and 2.6 ns at 1%, 5%, and 10% loading, respectively), whereas the OA + OAm system showed a much stronger concentration dependence (1.9, 2.0, and 2.9 ns at the same loadings). These results support the conclusion that the DDAB ligand enables higher effective PNC loading within PS—and therefore greater stopping power and, on average, greater energy deposition (and thus more light output) within the nanocrystals—a key factor for efficient scintillator performance.

Gulevich et al. investigated post-synthetic ligand exchange as a route to improving the PLQY of colloidal CsPbBr_3_ PNCs synthesized by hot-injection [137]. Unlike previous works, ligand exchange here was performed in the presence of perovskite precursors, intended to assist surface reconstruction during the procedure. Replacing part of the original oleic acid/oleylamine ligand shell with formamidinium bromide (FAB) alone, or with FAB plus added oleic acid, reduced PLQY (from 67% to 59% and 53%, respectively)—an effect the authors attribute to insufficient steric separation between nanocrystals. By contrast, treatment with DDAB increased PLQY in every formulation tested, with the strongest improvement—from 67% to 95%—achieved when DDAB was combined with additional PbBr_2_. FTIR spectroscopy confirmed that ligand exchange had occurred, evidenced by the disappearance of the oleic acid carbonyl and C–H stretching bands. The authors attribute DDAB’s effectiveness to three factors acting in combination: its ammonium cation and bromide anion provide dual passivation of both A-site and halide-vacancy defects; its shorter alkyl chain improves charge transport relative to oleic acid/oleylamine; and its two-chain structure packs into a denser, more stable ligand monolayer.

Samokhvalov et al. addressed a different limitation of ligand engineering: even when a more strongly binding ligand is used, incomplete reaction during synthesis can leave residual precursor contaminating the product [138]. Using tetradecylphosphonic acid as the surface ligand for hot-injection synthesis of CsPbBr_3_ PNCs—chosen for its higher surface-binding energy relative to oleic acid/oleylamine—the authors found that unreacted lead and cesium phosphonates remained in the product after standard precipitation-based purification, appearing as excess light scattering in the absorption spectrum. Passing the crude PNC solution through a gel permeation chromatography column removed these residues, suppressing the scattering and raising PLQY from 64% to 83% immediately after purification. The main benefit, however, was long-term stability: after 6 months of storage, unpurified PNCs retained only 27% of their PLQY and developed a visible precipitate, whereas purified PNCs retained 61% (comparable to their value immediately after synthesis) with no precipitate formation. This result indicates that, for phosphonic-acid-capped PNCs, removing unreacted precursor is at least as important for long-term stability as the choice of ligand itself.

Protection of PNCs from aggregation is important not only in solution-based synthesis but also in solid-state approaches such as mechanochemical milling. Ghosh et al. investigated how varying the amount of a single surfactant, oleylamine (OAm), in a two-step ball-milling method affects the size and emission properties of CsPbBr_3_ PNCs [139]. In their synthesis, 0.5 mM CsBr and 0.5 mM PbBr_2_ were loaded with zirconium oxide balls and milled for 1 h; varying amounts of OAm (0, 0.05, 0.1, 0.2, 0.4, or 0.8 mL) were then added, and milling continued for a further 45 min. The resulting PNCs were dispersed in 15 mL toluene, and after 24 h the precipitate was removed to yield a colloidal dispersion of CsPbBr_3_ PNCs. Scintillators were fabricated by incorporating these PNCs into a PMMA matrix: a PNC/PMMA mixture in toluene was held at 60 °C for 24 h, then poured into molds and left to fully solidify.

TEM analysis showed that adding 0.8 mL OAm (OAm0.8) produced spherical PNCs approximately 3.5 nm in size, while all other OAm amounts yielded cubic PNCs with sizes ranging from 36 nm (without OAm) down to 9 nm (with 0.4 mL OAm). This behavior reflects OAm’s role in dynamically stabilizing the CsPbBr_3_ surface through [Br···H–N^+^] hydrogen bonding between ammonium groups and edge halide atoms, which governs nanocrystal shape and size [140]. XRD measurements confirmed phase-pure formation of CsPbBr_3_ nanocrystals, with no peaks corresponding to residual CsBr or PbBr_2_. PLQY peaked at 88% (in solution) for the OAm0.2 sample, while PNCs synthesized without OAm showed the lowest PLQY, at just 7%. Beyond 0.2 mL OAm, PLQY declined again—possibly due to excess ligand causing light scattering and reduced PL quantum yield, or to formation of an amide byproduct from excess OAm that destabilizes the colloidal solution of PNCs. Despite this, OAm-capped PNCs showed excellent luminescence stability in open-air storage; the OAm0.4 sample, for instance, showed no change over 6 months.

Upon embedding into the PMMA matrix, PNCs tend to self-assemble and agglomerate, quenching their optical emission. An OAm concentration of 0.4 mL proved optimal for uniformly capping the nanocrystal surface, preventing agglomeration and preserving strong PL and RL; by comparison, 0.2 mL OAm led to larger agglomerates, while the excess at 0.8 mL reduced PLQY. Using the OAm0.4 PNCs, the authors then fabricated a series of scintillators with PNC loadings ranging from 1% to 10% in the matrix. The 2% CsPbBr_3_/PMMA scintillator showed the highest PL emission, attributed to optimal scattering and self-absorption at this loading, while the 10% sample showed the strongest RL emission, owing to greater X-ray attenuation at higher PNC concentration. Increasing PNC loading also produced a slight red-shift in the PL peak, likely reflecting some degree of PNC agglomeration and an increase in median nanocrystal size during mixing and casting. Incorporation into PMMA further enhanced PNC stability, preserving PL intensity unchanged during 60 days of storage in water.

Average PL lifetimes increased with OAm content, measuring 15.7, 25.0, and 38.5 ns for the OAm 0.1, 0.2, and 0.4 samples, respectively—consistent with progressive suppression of nonradiative decay pathways. RL decay times across the nanocomposite scintillators ranged from 38 to 55 ns, with the 10% PNC-loaded composite showing the shortest decay, likely due to enhanced radiative recombination at this loading. RL intensity increased with both higher OAm content (reflecting reduced nanocrystal agglomeration) and higher PNC loading (from 2% to 10%). Interestingly, however, the 2% sample showed higher PL than the 10% sample: since PL probes primarily near-surface emission, increased scattering at higher PNC loading reduces the detected PL signal. X-rays, by contrast, penetrate more deeply into the material, exciting a larger number of PNCs throughout the composite’s volume and thus producing stronger RL at higher loading. Finally, the scintillator fabricated with 10% PNC loading and a thickness of 2 mm achieved a spatial resolution of 8 lp/mm.

### 4.2. Doped PNCs in a Matrix

Doping refers to the introduction of small quantities of foreign ions into the crystal lattice of PNCs. Existing PNC doping strategies can be broadly classified into two general approaches. The first aims to dope PNCs with ions whose ionic radius closely matches that of the pristine PNC components—such as Ce^3+^, Cu^2+^, Zn^2+^, or Cd^2+^. Here, the dopants passivate surface traps and halide-vacancy defects, suppressing non-radiative recombination and thereby increasing both the luminescence quantum yield and the light yield of the scintillator. In the second approach, dopants with their own intrinsic emissive transitions (e.g., Mn^2+^, via its *d–d* transition) create new emission centers with a large Stokes shift, which virtually eliminates self-absorption and enables the fabrication of thicker scintillation films or scintillators with higher PNC loading. Doping can also enhance the stability of the PNC crystal lattice against external influences.

#### 4.2.1. Passivating Dopants

The studies presented in this section illustrate how dopants that act primarily as passivating agents for surface traps and halide vacancies can increase both the quantum yield and stability of PNCs without introducing a new emission pathway.

Lin et al. developed a scalable method for producing doped PNCs for scintillators using microfluidic synthesis [141]. The reaction was conducted at 90 °C by mixing, within a microfluidic cell, a precursor solution of Cs_2_CO_3_ and oleic acid in 1-octadecene with a second solution of PbBr_2_, oleic acid, and oleylamine in 1-octadecene. In addition to pristine CsPbBr_3_ PNCs, the authors fabricated Cu-doped PNCs with compositions CsPb_0.99_Cu_0.01_Br_3_, CsPb_0.94_Cu_0.06_Br_3_, CsPb_0.89_Cu_0.11_Br_3_, and CsPb_0.88_Cu_0.12_Br_3_ using the same microfluidic procedure and CuBr_2_ as the source of copper ions. The size, shape, and size dispersity of the PNCs varied with synthesis temperature and precursor injection rate: at 80 °C, PNCs were quasi-spherical with a diameter of approximately 6 nm; at 120 °C, ellipsoidal, approximately 8 nm in size, with a broader size distribution; and at 150 °C, cubic, with a narrow size distribution centered around 10 nm.

Among the doped samples, the highest PLQY (91%) was achieved by CsPb_0.89_Cu_0.11_Br_3_ PNCs, synthesized at 150 °C with a precursor injection rate of 20 µL/min. EDX imaging confirmed that Br, Cs, Pb, and Cu were evenly distributed throughout the nanocrystal ensemble, indicating that Cu^2+^ integrates smoothly into the PNCs structure. The authors studied the temperature dependence of PNC fluorescence. Despite the fact that raising the temperature from 50 to 300K reduced PL intensity for both doped and undoped PNCs, the Cu^2+^-doped sample consistently retained higher intensity throughout this heating range, confirming that Cu^2+^ incorporation passivates surface traps and enhances radiative efficiency.

Further experiments using PNC films—prepared by spin-coating PNCs with poly(ε-caprolactone) onto TiO_2_-coated glass—showed that Cu^2+^-doped PNCs possess markedly better stability than their undoped counterparts across multiple stress conditions. Under UV exposure, undoped PNC films lost 80% of their PL intensity after 50 days, compared with only 15% for the Cu^2+^-doped films, an improvement attributed to lattice distortion induced by doping. Under 50% humidity, the Cu^2+^-doped PNCs retained 80% of their initial PL after one day, versus only 60% for undoped PNCs. Under thermal stress at 400 K, the Cu^2+^-doped PNCs retained 70% of their PL intensity, compared with a drop to 25% for undoped PNCs.

Based on integrated RL intensity, using LuAG:Ce as the reference standard, the light yields of CsPb_0.89_Cu_0.11_Br_3_ and undoped CsPbBr_3_ PNCs were calculated as 21,340 and 11,710 photons/MeV, respectively, with a detection limit of approximately 82.5 nGy/s.

Wu et al. doped PNCs with Ce^3+^ ions, which partially replace Pb^2+^ within the nanocrystal structure, to increase the quantum yield of scintillators based on CsPbBr_3_ PNCs [142]. Doping was achieved by adding varying amounts of Ce oleate to the reaction mixture during nanocrystal synthesis; an 8% Ce^3+^ doping level enhanced the light yield of CsPbBr_3_ PNCs by more than a factor of two relative to undoped PNCs. TEM analysis showed that the as-synthesized Ce^3+^-doped CsPbBr3 PNCs retained a cubic shape similar to that of the pure CsPbBr_3_ PNCs.

**Figure 5:**
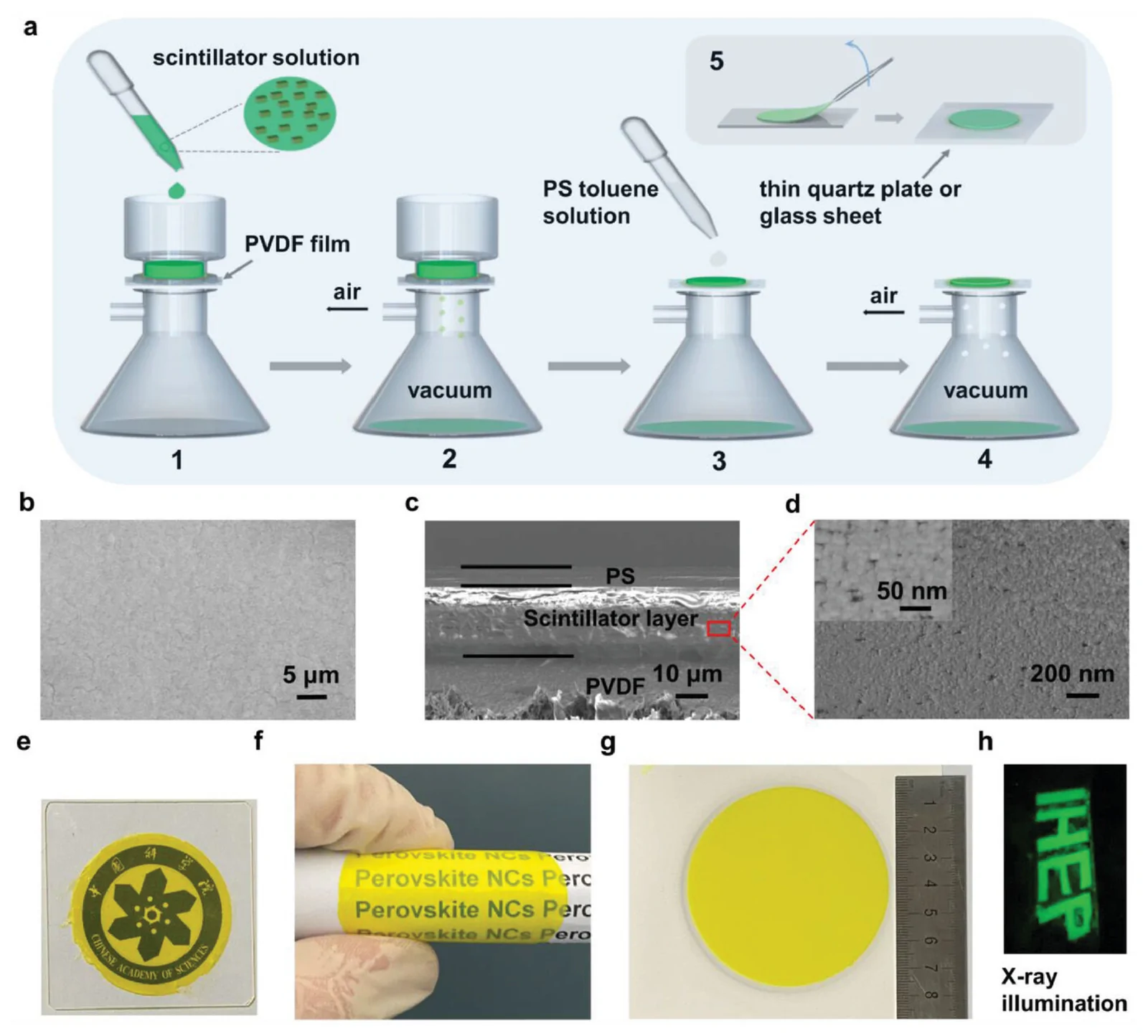
The procedure of preparation and characterization of Ce^3+^-doped CsPbBr_3_ scintillator film. (**a**) Scheme of the scintillator film synthesis procedure by the suction filtration method. (**b**) Top view of the scintillator film without PS protective layer. (**c**) SEM image of the side scintillator film with PVDF substrate and (**d**) its enlarged view. (**e**) Photograph of the scintillator film on the top of the logo. (**f**) Photographs of the flexible scintillator film and (**g**) a large-area scintillator film (50 cm^2^). (**h**) Photograph of the “IHEP” logo under X-ray illumination. Reprinted from Ref. [142].

To further improve the density and homogeneity of PNC distribution within the scintillator, the authors developed a method in which PNCs (at a concentration of 25 mg/mL) were deposited onto a PVDF membrane by filtration, followed by encapsulation in polystyrene (Figure 5). This approach enabled fabrication of scintillators with high PNC content, achieving an RL yield of 33,000 photons/MeV—10-fold higher than scintillators of similar thickness fabricated by spin-coating. The filtration method also produced a more uniform PNC distribution throughout the scintillator volume, enabling a spatial resolution down to 862 nm in an optical microscope under X-ray excitation, and improved scintillator transparency. At the same time, the high PNC density allowed for a reduction in overall scintillator thickness, helping to balance X-ray absorption, self-absorption, and transparency.

Notably, Ce^3+^ doping also enhanced the radiation stability of the scintillator: after 1200 s of continuous X-ray irradiation, the RL intensity of the 8% Ce^3+^-doped CsPbBr_3_ PNCs showed no measurable decrease, whereas undoped CsPbBr_3_ PNCs retained only 55% of their initial RL intensity. This improvement is attributed to the tendency of Pb^2+^ ions in perovskites to reduce to metallic Pb^0^ under heating or illumination, a process that otherwise diminishes the light yield of PNC-based scintillators [143].

Lead-free PNCs are gaining popularity, as they not only exclude toxic lead but also exhibit resistance to moisture, air, and prolonged UV irradiation. Qu et al. [144] described the fabrication of scintillators based on Cs_3_Cu_2_I_5_ PNCs and Zn^2+^-doped Cs_3_Cu_2_I_5_ PNCs. Colloidal Cs_3_Cu_2_I_5_ PNCs were synthesized via a hot-injection method, while Zn^2+^-doped analogues were obtained by adding varying amounts of ZnI_2_ directly to the precursor mixture (CuI, oleic acid, and octylamine in octadecene; Cs_2_CO_3_ with oleic acid in octadecene).

The authors found that higher injection temperatures and larger cesium-source volumes favored higher-quality PNCs. PNCs synthesized at low temperature (70 °C) contained more crystalline defects, whereas the reaction held at 180 °C yielded uniform, cubic-shaped PNCs with a minimum average size of ~16.5 nm; temperatures above 180 °C caused CuI to precipitate before injection of the Cs precursor, greatly reducing nanocrystal yield. At the optimal Zn/Cu ratio of 0.75, monodisperse cubic PNCs with an average size of 17.8 nm were obtained, and these retained their morphology for at least 10 days. For the Zn^2+^-doped Cs_3_Cu_2_I_5_ PNCs, higher Cs/Cu feed ratios substantially increased PLQY, reaching a peak of 92.8% at a Cs/Cu ratio of 4—compared with a maximum of only 72.2% for undoped samples—indicating that a Cs-rich environment combined with Zn^2+^ doping is ideal for self-trapped exciton emission. The PL lifetime of the Zn^2+^-doped PNCs was 3.7 µs, longer than that of the undoped PNCs (2.8 µs), consistent with suppressed nonradiative recombination.

To prepare the composite scintillator film, PNCs in cyclohexane were mixed with an EVA solution in cyclohexane, poured into a Petri dish, and dried at room temperature. Zn^2+^ doping markedly improved the surface uniformity of the resulting composite film: the Zn^2+^-doped Cs_3_Cu_2_I_5_@EVA film had a surface roughness of 12.6 nm—comparable to that of pure EVA (9.3 nm) and substantially smoother than the undoped PNC-based film (35.6 nm). This film also exhibited a water contact angle of ~100°, which indicates effective protection of PNCs from moisture. Embedding the doped PNCs into EVA reduced PLQY only moderately, to 67.2% for a 210 µm-thick scintillator, and the composite retained approximately 95% of its initial PL intensity after 10 days of air exposure.

The scintillating films achieved a detection limit of 0.31 µGy_air_/s (at a signal-to-noise ratio of 3) and a spatial resolution of 15.7 lp/mm in X-ray imaging. RL intensity showed a good linear response to X-ray dose rate and remained stable under 60 min of continuous X-ray irradiation at 0.2 mGy_air_/s.

Erroi et al. fabricated a scintillator based on CsPbCl_3_ PNCs doped with Cd^2+^ [81]. CsPbCl_3_ PNCs were synthesized via a modified ligand-assisted reprecipitation method using turbo-emulsification of two precursor mixtures. The first mixture contained a solution of Cs_2_CO_3_ in propionic acid, diluted with 180 mL of heptane/isopropanol (2:1 *v*/*v*); the second contained a solution of PbCl_2_ in a mixture of oleylamine, propionic acid, and isopropanol. After 30 s of homogenization in a turbo-emulsifier (15,000 rpm), the PNCs were precipitated by adding isopropanol and purified by centrifugation, yielding cubic PNCs with an edge length of 9 ± 1 nm.

To enhance the optical properties and stability of the PNCs prior to incorporation into a polymer matrix, the authors applied a post-synthetic treatment with CdCl_2_. A given amount of PNCs was dispersed in a mixture of lauryl methacrylate (LMA) and ethylene glycol dimethacrylate (EGDM) (80:20 vol%) together with a large excess of CdCl_2_, and the resulting solution was stirred vigorously for 3 h. The Cd-doped PNCs in the monomer mixture were then separated from unreacted excess CdCl_2_ by decanting. Poly(lauryl methacrylate) (PLMA), cross-linked with EGDM at an 80:20 volume ratio, was chosen as the scintillator host matrix for its nonpolar character and long alkyl side chains, which create a favorable chemical environment for the PNCs. To polymerize the matrix and form the scintillation film, a 2,2-dimethoxy-2-phenylacetophenone photoinitiator was added to the solution, which was then transferred into a casting mold and cured under UV light for 15 min.

The original (undoped) PNCs exhibited a low PLQY of ~3%, reflecting pronounced non-radiative losses, together with accelerated multi-exponential PL decay kinetics and an effective lifetime of ~250 ps. After CdCl_2_ treatment, PLQY increased 30-fold, to 93 ± 6%, and the decay kinetics became mono-exponential with a lifetime of 2.4 ns. RL from the doped PNCs likewise increased more than 20-fold, and RL intensity showed no measurable decrease even after a cumulative irradiation dose of 1 MGy—in stark contrast to conventional plastic scintillators (PTP and POPOP dyes in poly(vinyltoluene), PVT), which lost over 80% of their RL under comparable conditions.

Cd-doped PNCs fully retained their optical properties (PL spectrum, efficiency, and decay kinetics) upon incorporation into the polymer matrix, whereas the undoped PNCs underwent substantial PL quenching, with an effective PL lifetime dropping to just 110 ps in the composite. Light yield, measured by direct comparison under identical conditions against a plastic EJ276D scintillator of matched thickness, increased linearly with PNC concentration in the composite, reaching approximately 2100 photons/MeV at the highest loading tested (10%).

Overall, these results indicate that replacing surface Pb atoms with Cd eliminates deep hole traps caused by uncoordinated Cl^−^ ions. This substitution increases PLQY, yields purely excitonic scintillation free of slow, surface-defect-related decay components, confers high radiation hardness, and produces an ultra-short effective decay time of approximately 210 ps.

#### 4.2.2. Dopants with Intrinsic Emission

Unlike the dopants discussed above, which act mainly as surface-trap passivating agents, Mn^2+^ introduces its own emission center, characterized by a large Stokes shift. Xu et al. fabricated scintillators based on lead-free CsCdCl_3_ PNCs doped with Mn^2+^ [145]. CsCdCl_3_:xMn^2+^ PNCs were synthesized via the hot-injection method by degassing the precursors cesium acetate (CsOAc), cadmium acetate dihydrate (Cd(OAc)_2_ × 2H_2_O), and manganese acetate tetrahydrate (Mn(OAc)_2_ × 4H_2_O) in a mixture of oleic acid, oleylamine, and 1-octadecene at 110 °C, followed by rapid injection of chlorotrimethylsilane at 190 °C and immediate cooling. The product was purified by triple centrifugation using toluene and n-hexane, yielding cubic-shaped PNCs approximately 14.7 ± 3.1 nm in size; energy-dispersive elemental mapping confirmed a uniform spatial distribution of Cs, Cd, Mn, and Cl within the nanocrystals.

Maximum photoluminescence was achieved for PNCs with composition CsCdCl_3_:0.25Mn^2+^, whereas further increases in Mn^2+^ content disrupted the crystal lattice and quenched luminescence. The best surface passivation and suppression of non-radiative recombination were achieved at an OA:OAm ligand volume ratio of 8:1. The PLQY of CsCdCl_3_:0.25Mn^2+^ PNCs reached 82.7%, comparable to that reported for Mn^2+^-doped CsPbI_3_ PNCs [146]. Upon 280 nm excitation, the fluorescence lifetime was 18.75 ms—characteristic of Mn^2+^ emission [147]—with the millisecond timescale and single-exponential decay indicating a single radiative process associated with the *d–d* transition of Mn^2+^ ions [148].

Mn^2+^ doping also substantially enhanced PNC stability. The doped PNCs fully preserved their structure after 6 months of storage in open air or 10 h of heating at 70 °C, and retained up to 97% of their PL intensity after 8 h of UV irradiation and 93% after 120 h of storage at 65% relative humidity. RL intensity showed no decrease after 1.5 h of X-ray irradiation at 5.7 mGy/s. Owing to the high atomic number of its constituent elements, CsCdCl_3_:0.25Mn^2+^ exhibited X-ray absorption efficiency comparable to commercial BGO and LYSO scintillators, along with an outstanding light yield of 54,500 photons/MeV, a linear RL dose response, and a detection limit of 28.97 nGy/s. Finally, a scintillation film fabricated by incorporating these PNCs into PMMA achieved a spatial resolution of 17.5 lp/mm.

### 4.3. Energy Transfer in PNC-Based Hybrid Scintillators

A further increase in scintillator efficiency can be achieved by combining PNCs with functional components of a different nature. The principal physical mechanisms underlying this approach are Förster resonance energy transfer (FRET) and radiative energy transfer (reabsorption) between the components. Classical inorganic scintillators possess high stopping power but a slow response time. Depositing a PNC layer onto their surface, or forming heterostructures with them, makes it possible to reshape the emission spectrum to better match the peak sensitivity of the photodetector and to increase the overall light yield. An alternative strategy involves adding organic dyes to the PNCs: these dyes act as energy acceptors, absorbing energy from the PNCs and re-emitting light at wavelengths the PNCs themselves do not absorb, thereby suppressing self-absorption and enabling the fabrication of bulk scintillators with high light yield.

#### 4.3.1. Energy Transfer to PNCs as Acceptors

In the studies described below, an inorganic or organic plastic scintillator acts as the energy donor, transferring energy to PNCs as the acceptor, in order to shift the emission spectrum and increase the overall light yield of the resulting energy-transfer system.

Knysh et al. demonstrated an energy-transfer strategy based on a classical multi-component plastic-scintillator design [66]. CsPbBr_3_ PNCs (~0.2 wt%) were embedded in a radically polymerized para-methylstyrene/divinylbenzene matrix to form a two-component composite (PNC-pMeSt). Under X-ray excitation, this composite produced effective radioluminescence at 514 nm, matching the PNC’s own photoluminescence peak—consistent with the idea that radioluminescence occurs via FRET from polymer matrix excitons to the PNCs. However, under harder excitation from gamma rays emitted by a ^137^Cs source (661.7 keV), analyzed by pulse-height gamma spectroscopy, the PNC-pMeSt sample showed no resolvable Compton edge, indicating a very low effective light yield under these conditions. The authors attribute this to self-absorption of emitted light within PNC aggregates, combined with weak spectral overlap between the matrix’s own emission and the PNC absorption band.

Adding a second fluorophore, POPOP (0.15 wt%), as an intermediate re-emitter in a three-component polymer/POPOP/PNC cascade—following the same multi-stage energy-transfer design used in classical plastic scintillators—restored a clearly resolvable Compton edge, with a light output of approximately 13% of an anthracene reference, corresponding to roughly 2000 photons/MeV.

Jegal et al. investigated the effect of depositing a PNC layer onto classical scintillators as a means of increasing their light yield [149]. To test this concept, GAGG (gadolinium aluminum gallium garnet scintillation crystal), LYSO (lutetium yttrium orthosilicate scintillation crystal), and CdWO_4_ scintillators were selected as the base materials for this system. Each of these scintillators was coated with a mixed solution of CsPbBr_3_ PNCs (Perovskite ABX3 Quantum Dots, 510 nm; Quantum Solutions, Southampton, UK) and PMMA in toluene via drop-casting, and the resulting samples were dried for 30 min to remove the solvent. This process produced a PNC-in-PMMA film 9–13 µm thick on the surface of each inorganic scintillator.

The calculated FRET efficiency between the PNCs (acceptor) and the GAGG, LYSO, and CdWO_4_ scintillators (donors) was 1.0, 0.579, and 0.429, respectively, corresponding to average light yield increase of 51%, 36%, and 31%. The addition of the PNC layer also slightly improved the energy resolution of the scintillators under gamma-ray excitation across various energies, while maintaining radiation stability at the 99% level over two days of ^137^Cs irradiation.

Braddock et al. investigated the effect of adding hybrid organic–inorganic formamidinium-based FAPbX_3_ PNCs (X = Cl, Br) to a commercial plastic scintillator to form a nanocomposite, and demonstrated its application for low-cost X-ray imaging [150]. Formamidinium was chosen as the A-cation for its ability to fine-tune the emission wavelength across the entire visible range by varying the halide composition. The PNCs were synthesized by ligand-assisted reprecipitation at room temperature in a mixture of octylamine, oleic acid, and toluene, using the corresponding precursors: lead bromide and formamidinium bromide for FAPbBr_3_; lead chloride and formamidinium chloride for FAPbCl_3_; or a mixture of both for FAPbBr_3x_Cl_3(1−x)_ compositions. Emission wavelength varied linearly with halide ratio, from 403 nm (FAPbCl_3_) to 531 nm (FAPbBr_3_), allowing emission to be tuned to match the peak quantum efficiency of a given photodetector. Since the PL intensity of FAPbBr_3_ was approximately 10,000 times higher than that of FAPbCl_3_, FAPbBr_3_ was selected for fabricating the nanocomposite scintillators.

To prevent PNC aggregation, the authors employed a bifunctional ligand, bis [2-(methacryloyloxy)ethyl] phosphate (BMEP): its P–OH head group binds strongly to the PNC surface, while its vinyl group co-polymerizes with the polyvinyltoluene (PVT) matrix of the plastic scintillator, preventing PNC aggregation during polymerization. Partial ligand exchange with BMEP reduced the PL intensity of the PNCs in solution by 23%, but yielded a gain in scintillator transparency expected to level this loss in fluorescence. Scintillation discs 1 mm thick were fabricated by solution processing, using either inert PMMA or EJ-290 plastic scintillator (supplied as a three-part kit) as the matrix, with FAPbBr_3_ PNC loadings of 0.4, 0.9, and 1.8 wt%.

BMEP modification was shown to reduce nanocrystal aggregation: the transparency at 600 nm of 1.2 mm-thick scintillators based on BMEP-modified PNCs was 18% higher than that of 0.9 mm-thick scintillators without BMEP modification at an equivalent loading, despite the greater thickness of the modified samples. Nonetheless, overall transmission of the FAPbBr_3_@BMEP/PMMA scintillator remained low, owing to the small Stokes shift of FAPbBr_3_ (50 meV), which causes self-absorption and reduces the scintillator’s quantum yield. The RL spectra of the nanocomposites showed suppression of the intrinsic EJ-290 emission peaks (at 423 and 445 nm) alongside the emergence of a single peak from FAPbBr_3_, whose intensity increased with PNC loading. This suppression of the high-energy emission characteristic of the base plastic scintillator indicates energy transfer from the EJ-290 components to the PNCs—occurring either through absorption of light emitted by the plastic scintillator’s organic dyes, or via a nonradiative FRET-type mechanism.

Pure EJ-290 has a very fast fluorescence decay time of 1.2 ns, typical of organic dyes. Adding FAPbBr_3_ PNCs to the EJ-290 scintillator increased overall PL lifetime, though this lifetime declined gradually with increasing PNC loading: measured values were 25.0 ns (0.4%), 21.8 ns (0.9%), and 20.3 ns (1.8%). This faster overall decay at higher loadings reflects a decrease in the two decay-time components associated with the nanocrystals, likely linked to increased energy transfer from the plastic scintillator matrix to the perovskite nanocrystals as acceptor concentration rises. Notably, the FAPbBr_3_/PMMA composite at 1.8% loading showed a substantially longer fluorescence decay time of 62.0 ns—more than three times slower than FAPbBr_3_/EJ-290 at the same loading—providing further evidence of efficient energy transfer between the EJ-290 scintillator components and the FAPbBr_3_ PNCs.

In light yield measurements at a dose rate of 0.68 Gy/s, the number of registered counts for FAPbBr_3_/EJ-290 was 1.9 times that of pure EJ-290, while FAPbBr_3_/PMMA at an equivalent loading reached 3.8 times that of EJ-290. These results indicate that the EJ-290 matrix provides a faster response due to energy transfer, whereas the PMMA matrix yields substantially brighter emission.

**Figure 6:**
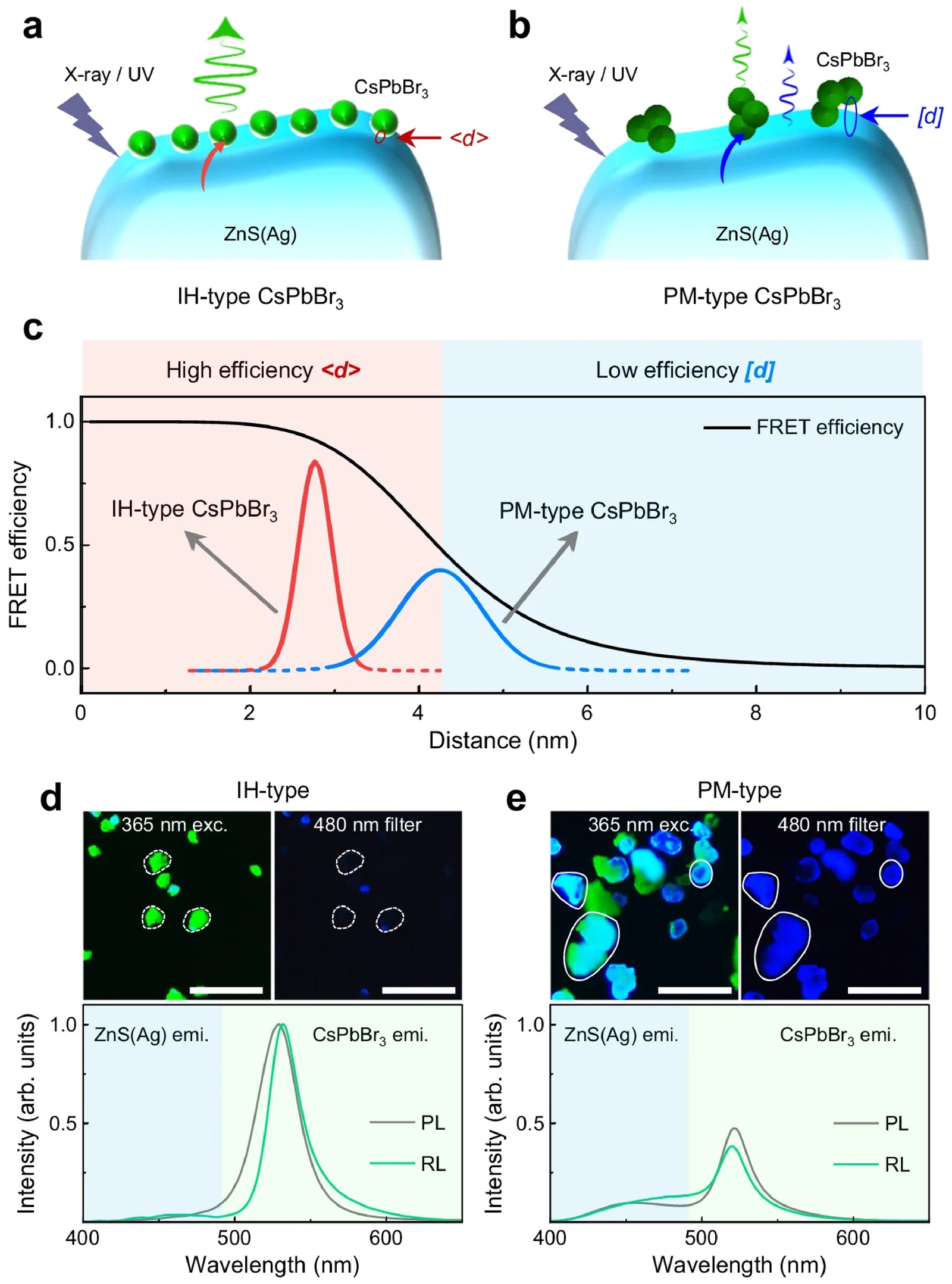
Schematic of two kinds of structural configurations of the CsPbBr_3_/ZnS(Ag) hybrid scintillator: (**a**) IH-type CsPbBr_3_ and (**b**) PM-type CsPbBr_3_. <d> and [d] are the short and long average distances between CsPbBr_3_ PNC and ZnS(Ag) microcrystals, respectively. (**c**) Calculated FRET efficiency of two kinds of structural configurations with various donor–acceptor distances. The narrow distribution of <d> represents that most of the IH-type CsPbBr_3_ PNC are located in the high-efficiency region. The wide distribution of [d] represents that a few PM-type CsPbBr_3_ PNC are located in the high-efficiency region and others are located in the low-efficiency region. Fluorescence microscopy images and PL and RL spectra of (**d**) IH-type CsPbBr_3_ and (**e**) PM-type CsPbBr_3_. Scale bars: 50 μm. The samples were excited by 365 nm light, and a 480 nm narrow-band optical filter was added into the microscopic optical path to identify the position of ZnS(Ag) by eliminating the green emission of CsPbBr_3_. The invisible ZnS(Ag) particles in the IH-type CsPbBr_3_ are marked with dashed white circles. The strong blue-emitting ZnS(Ag) particles in the PM-type CsPbBr_3_ are marked with solid white circles. Reprinted from Ref. [151].

Yang et al. succeeded in increasing the light yield of hybrid scintillators through FRET-based energy transfer from ZnS(Ag) microcrystals to CsPbBr_3_ PNCs [151]. As the energy donor, the authors used silver-doped ZnS microcrystals (ZnS:Ag), several micrometers in size, which are traditionally employed as inorganic scintillators [152]. To clearly establish the effect of FRET on the hybrid’s light yield, they fabricated two distinct types of heterostructures (Figure 6). The first, designated IH-ZnS(Ag)-CsPbBr_3_, was produced by solid-phase ultrafast grinding at speeds above 15,000 rpm, during which ZnS(Ag) and the Cs, Pb, and Br precursors are rapidly and uniformly mixed through collision, friction, and shear forces; with increasing grinding time, CsPbBr_3_ PNCs grow directly on the surface of the ZnS(Ag) microcrystals. This fabrication method enables the close proximity between the ZnS microcrystals and the PNCs required for efficient FRET [153]. The second system, designated PM-ZnS(Ag)-CsPbBr3, was instead obtained by direct physical mixing of commercial ZnS(Ag) powder with separately solid-phase-synthesized CsPbBr_3_ PNC powder.

TEM analysis showed that the ZnS(Ag) microcrystals in the IH-ZnS(Ag)-CsPbBr_3_ structures were densely covered with non-aggregated PNCs. XRD analysis of the heterostructures showed that most nanocrystals retained the normal CsPbBr_3_ perovskite structure, with only a small fraction present as CsPb_2_Br_5_. X-ray photoelectron spectroscopy revealed the presence of Pb–S bonds, confirming that the PNCs had grown directly on the microcrystal surface. Fluorescence spectroscopy of the two heterostructure types showed that the IH heterostructures exhibited intense green emission, characteristic of CsPbBr_3_ PNCs, with no discernible blue emission from ZnS(Ag). By contrast, the PM heterostructures showed only weak green emission against a background of strong blue emission—indicating that high FRET efficiency was achieved only in the IH heterostructures.

Scintillators based on both heterostructure types were fabricated by mixing them with powdered polyethylene and baking for 10 min at 120 °C under 10 MPa pressure. After polishing, this yielded flexible thin scintillation films approximately 0.2 mm thick, with homogeneous emission properties confirmed by optical and scanning electron microscopy. Scintillation testing showed that, relative to a BGO reference scintillator, the IH heterostructures achieved a light yield of 40,000 photons/MeV—1.3 times higher than the 30,000 photons/MeV measured for the PM heterostructures—indicating more efficient energy transfer in the IH system. Correspondingly, the IH heterostructures achieved a detection limit of 70 nGy_air_/s, substantially better than the 300 nGy_air_/s measured for the PM heterostructures. Decay time was identical across all heterostructures and pure PNCs, at 36 ns. The IH heterostructures also achieved higher spatial resolution than the PM heterostructures (30 lp/mm versus 14 lp/mm), a result attributed both to their improved light yield—which provides higher brightness with fewer noise points—and to reduced light scattering, owing to the more uniform distribution of less-agglomerated emitters within the IH sample.

#### 4.3.2. PNCs as Donors for Energy Transfer

**Figure 7:**
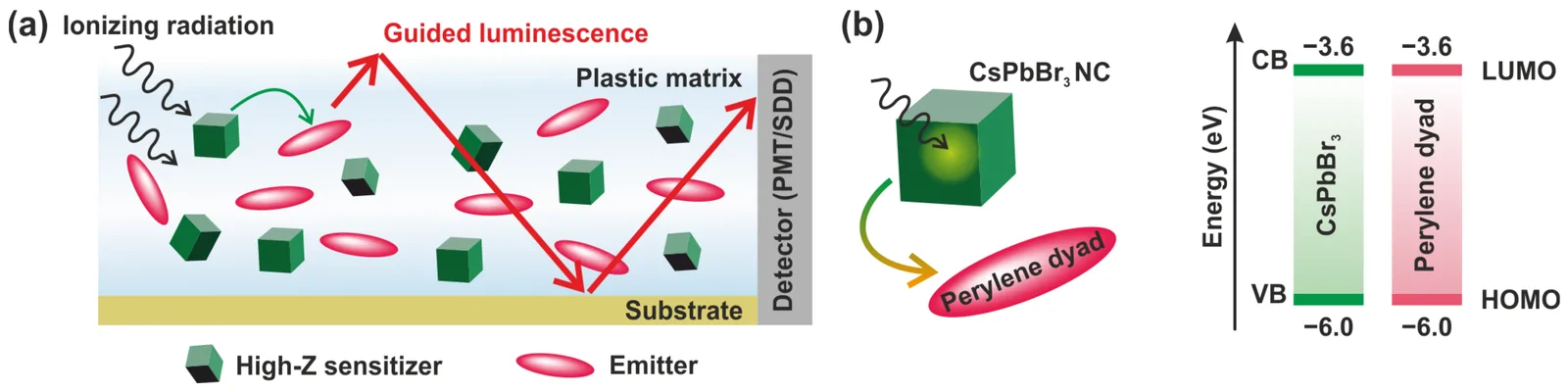
Schematic depiction of a high-Z sensitized plastic scintillator based on CsPbBr_3_ and perylene dyad dye. (**a**) Sketch of a CsPbBr_3_ NC sensitizing the perylene dyad 1. The energy levels of the two systems are also shown (**b**), to highlight the resonance between the respective frontier levels, which is important to prevent charge separation between the two species leading to slow indirect exciton decay or efficiency losses by exciton splitting. CB, conduction band; VB, valence band. Adapted from Ref. [154].

As PNC loading in scintillators increases, a decrease in light yield associated with self-absorption of emitted photons begins to emerge, reducing scintillator efficiency and precluding the fabrication of large-volume or high-density scintillators. Gandini et al. proposed a solution to this problem [154], combining CsPbBr_3_ PNCs with the organic fluorescent perylene dyad dye 9,9′-bis[perylene-3,4-dicarboxylic-3,4-(N-(2,5-di-tert-butylphenyl)] (PD) within a polymer matrix. The highest occupied molecular orbital (HOMO) and lowest unoccupied molecular orbital (LUMO) levels of PD are nearly isoenergetic with the valence and conduction bands of CsPbBr_3_ PNCs, producing near-perfect resonance between the PNC emission spectrum and the optical absorption of PD. This resonance in turn enables reabsorption-free waveguiding of the sensitized scintillation light (Figure 7).

Studies of PNC/PD mixtures in toluene showed that PNC luminescence decreased with increasing PD concentration, accompanied by a shortening of the PNC PL lifetime—both indicating efficient energy transfer from the PNCs to the PD dye. Scintillators based on PNCs alone, PD alone, and PNC/PD mixtures were then fabricated using PMMA as host matrix. Embedding the PNCs in PMMA had almost no effect on PL lifetime (τ_Fast_ ≈ 23 ns and τ_Slow_ ≈ 180 ns in toluene, versus τ_Fast_ ≈ 30 ns and τ_Slow_ ≈ 215 ns in PMMA), though it slightly reduced PLQY, from 75 ± 8% to 65 ± 5%. In scintillator samples containing 0.15 wt% PD dye, the PNC PL lifetime decreased markedly, indicating that nonradiative energy transfer to PD had become the dominant decay channel for the PNCs.

The best overall scintillator performance—both in RL intensity (approximately 9000 photons/MeV) and in stability and PL lifetime (approximately 3.4 ns)—was achieved using 2 wt% PNCs combined with 0.15 wt% PD. Furthermore, incorporating the PD dye into a large bulk scintillator substantially reduced RL signal losses due to self-absorption, limiting the loss to only 25% for a scintillator having a 12 cm length.

#### 4.3.3. Plasmonic (Purcell) Enhancement of PNC Scintillation

Boosting semiconductor fluorescence through engineering of light–matter interactions—specifically by exploiting Purcell enhancement—has been demonstrated in numerous hybrid systems, including photonic crystals [155] and plasmonic materials [156]. These techniques were later applied to enhance the scintillation efficiency of quantum-dot-based scintillators [157] and inorganic thin-film scintillators [158,159].

Makowski et al. demonstrated the first practical application of the Purcell effect to bulk-scale (millimeter-thick) PNC scintillators, extending beyond the ultrathin films to which plasmonic enhancement had previously been confined [160]. CsPbBr_3_ PNCs were synthesized via hot-injection and combined, at 20 wt% PNC loading, with commercial silver nanoparticles at a 1:1 PNC-to-nanoparticle weight ratio. Two nanoparticle morphologies were used—100 nm spheroids (SNPs) and 100 nm cuboids (CNPs)—each coated with a 5 nm poly(vinylpyrrolidone) (PVP) capping layer. The mixtures were dispersed in PDMS, then cast and cured into 1.5 cm-diameter, 5 mm-thick discs—roughly two orders of magnitude thicker than earlier plasmonic scintillator demonstrations. TEM imaging and elemental mapping confirmed that the CsPbBr_3_ PNCs were well attached to, and uniformly distributed across the Ag nanoparticle surfaces, with a nanocrystal-to-nanoparticle coupling percentage of (70 ± 8)%.

The authors then carried out an extensive characterization of both steady-state and time-resolved PL and RL properties of the composites, finding significant enhancement in both intensity and decay rate. This enhancement arises from the Purcell effect: the Ag nanoparticles modify the local density of optical states around nearby PNCs, increasing their radiative decay rate without direct charge transfer, since the insulating PVP layer physically separates the metal surface from the nanocrystals. This mechanism is supported by the observation that all measured enhancements manifested as decay-rate shortenings, rather than the decay-time prolongations that would be expected from charge transfer.

Experimentally, at room temperature, the SNP- and CNP-doped samples showed integrated PL intensity enhancements of 3.20-fold and 4.10-fold, respectively, relative to control CsPbBr_3_/PDMS composite. The fastest time-resolved PL decay component shortened from 0.99 ns (undoped) to 0.71 ns (SNP) and 0.61 ns (CNP), corresponding to PL decay-rate enhancements of 2.48-fold and 4.20-fold. Under X-ray excitation, the same trend held but at reduced magnitude: RL intensity enhancements of 1.71-fold (SNP) and 1.92-fold (CNP), with RL decay-rate enhancements of 1.84-fold and 2.08-fold, and average RL decay time dropping from 7.55 ns (undoped) to 4.18 ns (SNP) and 3.70 ns (CNP). Under excitation from a ^241^Am source, the CNP-doped sample’s absolute light yield—calibrated spectroscopically—increased from 4100 photons/MeV (undoped) to 8500 photons/MeV, a 2.07-fold enhancement. The authors note that this undoped-sample baseline is already lower than the ~21,000 photons/MeV reported elsewhere for CsPbBr_3_ PNCs in other host media, attributing the reduction specifically to the PDMS host; accordingly, they emphasize the demonstrated enhancement factor, rather than the absolute light yield, as the more broadly transferable result.

In X-ray imaging, maximum image intensity rose from 1000 counts (undoped) to 1950 counts (SNP) and 2250 counts (CNP)—enhancements of 1.95-fold and 2.25-fold—with a best spatial resolution of 5 lp/mm. Cooling to 80 K further increased the PL enhancement, to 5.74-fold (SNP) and 6.52-fold (CNP), consistent with reduced non-radiative losses at low temperature. The authors conclude that these findings establish a viable pathway for using nanoplasmonics to enhance bulk scintillator devices, advancing PNC-based radiation detection technology.

## 5. Matrix-Free PNCs

Abandoning the polymer matrix as a component of a PNC-based scintillator eliminates the principal “diluting” factor: in a scintillator consisting solely of PNCs, the volume fraction of active material is maximized, which increases both stopping power and light yield per unit thickness. As shown previously, the ordering of PNCs within a scintillator affects spatial resolution; however, PNC organization also affects light yield and stopping power. Fabricating scintillators that form a densely packed PNC solid is therefore an important goal.

Zhang et al. proposed forming thin scintillation films from CsPbBr_3_ nanosheets [161]. The nanosheets were synthesized from two precursor solutions. First, a Cs precursor solution was prepared by dissolving 32 mg of cesium acetate in 1 mL of 1-propanol (1-PrOH) in a 20 mL vial with stirring in air at room temperature, followed by the addition of 6 mL hexane and 2 mL 1-PrOH. Second, a Pb(II) precursor solution was prepared by dissolving 245 mg of PbBr_2_ in a mixture of 0.45 mL 1-PrOH, 0.45 mL octanoic acid, and 0.45 mL octylamine at 90 °C in air, under vigorous stirring. The hot PbBr_2_ precursor was then rapidly injected into the Cs precursor solution at room temperature under vigorous stirring, with the reaction completing within 2 min. Thin-film scintillators were obtained by drying a drop of the resulting PNC solution in toluene (at concentrations up to 150 mg/mL) on a glass surface under ambient conditions.

TEM analysis of these films showed that the 2.8–4.2 nm-thick CsPbBr_3_ nanosheets stack into piles of 5–7 layers, with an interlayer spacing of approximately 2.1 nm—corresponding to two layers of surface ligands (octylamine or octanoic acid) covering the PNC surface. PL profile measurements and PL decay kinetics established that interparticle energy transfer occurs between thinner and thicker nanosheets, with a calculated FRET efficiency of 74%; decay times ranging from 2 to 13 ns across different wavelengths further confirmed the presence of FRET within the ensemble. The resulting thin-film scintillator, formed by drop-drying, had a thickness of approximately 4 µm and a surface roughness of about 13 nm.

PNCs typically show a decrease in fluorescence quantum yield when transitioning from colloidal solution to the solid state—associated with partial loss of the PNC ligand shell—with light yield reductions of up to threefold reported elsewhere [162]. In the fabricated thin-film scintillator, however, the stacked nanosheets retained a high PL quantum yield (up to 63%), largely because the ligands bound to the nanosheets remained well shielded between the stacked layers. The RL intensity of the colloidal CsPbBr_3_ nanosheet solution was 21,000 photons/MeV—20% higher than that of its solid-state counterpart. For the solid films, the authors report that RL intensity increased with scintillator thickness, reaching a plateau at 25 µm—substantially thinner than the composite scintillators described by Yang et al. [151] (approximately 0.5–1 mm)—indicating a high PNC density in the fabricated scintillators.

Similar results were demonstrated by Wang et al. [163]. CsPbBr_3_ nanosheets were synthesized by introducing a PbBr_2_ solution (heated to 100 °C, in isopropanol, octylamine, and octanoic acid) into a cesium acetate solution (in isopropanol and n-hexane). High-resolution TEM confirmed the formation of both thin and thick CsPbBr_3_ nanosheets, with widths ranging from 3.5 to 9.3 nm. Scintillation films obtained by drop-casting—via solvent (toluene) evaporation over 15 min—had a thickness of approximately 15 µm, with linear dimensions reaching up to 5 cm. The authors showed that scintillation film thickness could be tuned by adjusting the PNC concentration in solution: the best spatial resolution was achieved for scintillators 15 µm thick, whereas films 8 µm and 38 µm thick produced blurred, low-contrast images. Decay-time analysis revealed two components in the fluorescence decay curve, indicating resonant energy transfer between thin and thick CsPbBr_3_ nanosheets. This interpretation was further supported by XRD measurements showing an interlayer spacing of approximately 2.4 nm between nanosheets—close to the calculated FRET distance of 2.9 nm for nanosheet stacks.

Guo et al. fabricated thin scintillation films based on lead-free Cs_3_Cu_2_I_5_ perovskite microcrystalline phase without a polymer or glass matrix protection [164]. The Cs_3_Cu_2_I_5_ film was synthesized by conventional chemical vapor deposition (CVD) from CuI and CsI (3:2 molar ratio) under a nitrogen atmosphere: the mixture was heated to 540 °C at 10 °C/min, held at this temperature for 1 h, and then slowly cooled to room temperature, yielding a white, crack-free perovskite thin film on a glass substrate. A homogeneous distribution of Cs, Cu, and I, together with the formation of Cs_3_Cu_2_I_5_, was confirmed by scanning electron microscopy with energy-dispersive spectroscopy and by XPS measurements. The film exhibited a strong ultraviolet absorption peak at 240–300 nm and broad blue photoluminescence peaking at ~445 nm, with a PLQY of 85.14% under 300 nm excitation. Fitting the PL decay curve at the 445 nm emission maximum to a single-exponential function gave a PL lifetime of 1.12 µs.

Air stability was assessed by exposing the as-prepared film to ambient air for 60 days and comparing its structural purity and PL properties with those of a fresh sample. XRD patterns showed no change after 60 days, with all peaks indexed to the Cs_3_Cu_2_I_5_ phase, confirming excellent air stability; PL emission profiles also remained unchanged, PLQY stayed above 50% (at 58.32%), and the PL lifetime measured 1.05 µs. The film’s effective atomic number was calculated to be 51.1, with a density of 4.5 g/cm^3^—both favorable for efficient absorption of high-energy photons. Consequently, the RL light yield of Cs_3_Cu_2_I_5_ (39,251 photons/MeV) was almost twice that of commercial LuAG:Ce (22,000 photons/MeV), higher than that of lead halide CsPbBr_3_ nanocrystal scintillators (~21,000 photons/MeV [161]), and comparable to other lead-free halide scintillators such as K_2_CuBr_3_ (23,806 photons/MeV) [165]. The lowest detectable dose rate was 0.815 µGy/s, with excellent linearity across a broad range from 25 to 188 µGy/s and stable radioluminescence after 1.5 h of irradiation at 0.2 mGy_air_/s. Finally, the film achieved an X-ray imaging resolution of up to 100 lp/mm—substantially higher than that reported for PNCs dispersed in various matrices. Overall, although this particular work studied a non-nanocrystalline, CVD-grown Cs_3_Cu_2_I_5_ film, the material’s intrinsic nontoxicity, high radioluminescence intensity, and good stability suggest it holds promise as a future competitive material for X-ray imaging.

Detectors with enhanced sensitivity for high-energy particles are desirable for elementary particle physics, astronomy, and proton therapy. Modern ones typically rely on bulk crystals, since thin-film organic scintillators suffer from low light yield and limited radiation hardness. Mi et al. presented transmissive thin-film scintillators made of CsPbBr_3_ nanocrystals for real-time single-proton counting [166]. The PNCs were synthesized via hot-injection: PbBr_2_ was dissolved in oleic acid, oleylamine, and octadecene at 180 °C, while Cs-oleate was prepared separately from Cs_2_CO_3_ in oleic acid and octadecene; 10 s after injection, the reaction was cooled to 50 °C. The resulting cubic PNCs (~7.8 nm) were collected by centrifugation and stored in toluene. To passivate surface defects, the PNCs were treated twice with an aqueous ammonium hexafluorosilicate solution, for 1 h and then 2 h; this treatment increased PLQY from ~63% to ~81% in solution (~71% in film). Thin films were then fabricated by blade-coating onto a 500 nm pioloform membrane at room temperature.

Upon excitation at 365 nm, the 35.1 µm-thick scintillator emitted at 526 nm (FWHM 29 nm, Eg = 2.36 eV). Under 2 MeV proton irradiation, the emission red-shifted to ~537 nm (FWHM ~21 nm), as it also did under X-ray and near-infrared (~980 nm) excitation. The red shift observed under UV, NIR, and X-ray excitation is attributed to reabsorption—a process absent in solution—whereas the shift under proton irradiation is instead attributed to biexciton formation. Consistent with this assumption, the PL lifetime under UV/NIR excitation was ~10 ns, but dropped to ~336 ps under proton irradiation, indicating an efficient Auger recombination process characteristic of multiexciton formation.

Using a customized optical setup, the authors demonstrated single-proton counting with the CsPbBr_3_ nanocrystal films: the detected photon pulse height scaled linearly with proton count across a measurement range of ~10^2^ to 10^5^ protons. The conversion efficiency of the ~5 µm-thick PNC scintillator was approximately twice that of the commercial BC-400 plastic scintillator (~15 µm thick) and roughly 10 times higher than that of bulk LYSO:Ce, BGO, and YAG:Ce; the estimated light yield was 2.9 × 10^5^ photons/MeV. The minimum detectable flux was 7 protons/s, and ionoluminescence remained stable over 2 h under a proton fluence of 10^11^/cm^2^. Finally, proton imaging using a 120 µm-thick film, a Ni grid, and a 1024 × 1024 pixel array achieved a lateral resolution of 23.1 nm horizontally and 39.7 nm vertically. The authors conclude that these characteristics raise the prospect of achieving dose control with single-proton precision in proton-irradiation applications. While a number of obstacles remain to be overcome prior to clinical deployment of these materials as proton dose meters, CsPbBr_3_ nanocrystal scintillators nonetheless represent a highly promising platform for advancing the emerging detection technology in proton therapy and radiography.

The studies reviewed in the last two sections show that separating PNC synthesis from matrix formation allows for much finer control over nanocrystal size, composition, and surface chemistry than the in situ route permits—at the cost of an additional processing step and, in some cases, PNC aggregation during loading into the matrix. This finer control is reflected directly in scintillator performance: solution-synthesized PNCs achieve substantially higher peak light yields and spatial resolutions than any in situ system discussed in Section 1. Ligand and matrix engineering occupy a central role in this context, precisely because ligand chemistry—rather than matrix chemistry—now governs PNC quality; doping and coupling PNCs with an energy donor or acceptor further allows specific performance parameters, such as spatial resolution or self-absorption, to be tuned independently. Finally, the matrix-free approaches discussed above demonstrate that, for several applications, a supporting matrix is not strictly required at all.

Table 5 summarizes the performance parameters reported for these pre-synthesized PNC-based scintillators. The highest light yield in the table, 40,000 photons/MeV, was achieved with a FRET-based energy transfer system from ZnS(Ag) microcrystals to CsPbBr_3_ PNCs—about twice the average value reported for the best in situ-grown scintillator in Table 4—and the best detection sensitivity, 13 nGy/s, is likewise almost an order of magnitude lower than that reported for the best in situ-produced system. Spatial resolution shows a wide spread, ranging from 2 lp/mm up to 580 lp/mm for a Ce^3+^-doped PNC film, the highest resolution reported among all studies reviewed. Several works also report excellent composite stability under much higher cumulative doses (into the kGy–MGy range) than are typically tested for in situ-produced scintillators; this likely reflects differences in the specific applications targeted by each study (e.g., high-energy-physics dosimetry versus medical imaging) rather than an intrinsic difference in radiation hardness between the two synthesis routes.

Matrix-free scintillators match or exceed several of these metrics without any supporting polymer or glass host: a CVD-grown Cs_3_Cu_2_I_5_ perovskite film reaches 100 lp/mm with a PLQY of 85%, and PNCs developed for proton-beam imaging report light yields of up to 290,000 photons/MeV under proton excitation—though this figure is not directly comparable to the electron- or X-ray-excited values discussed above, owing to the very different energy-loss mechanisms involved in proton-excited scintillation. This suggests that, for several applications, embedding PNCs in a matrix is a matter of convenience for handling and integration, rather than a strict performance requirement.

## 6. Conclusions and Outlook

Across the two synthesis routes reviewed here—in situ growth of PNCs within a preformed matrix, or dispersion of pre-synthesized PNCs into a matrix—a consistent picture emerges. The in situ route trades some control over nanocrystal quality for the practical benefit of single-step, matrix-integrated fabrication, making it particularly well suited to applications where mechanical robustness or moisture protection is paramount. The pre-synthesized route, by contrast, allows finer control over PNC size, composition, and surface chemistry, and correspondingly achieves the highest peak light yields, spatial resolution, and detection sensitivity reported—though at the cost of an additional processing step. Both routes converge on the same underlying design challenge: chemical optimization of the PNCs must be carried out in a way that remains consistent with the radiation-absorption and light-extraction pathways that make them useful as scintillators.

Prior to discussing PNC scintillator optimization strategies, we would like to highlight the importance of environmental and health safety considerations when introducing PNC-based products into real-world applications. The safety of large-scale PNC use must be carefully addressed, since most PNC compositions contain heavy lead atoms; moreover, some studies have explored cadmium doping as a strategy for improving PNC fluorescence. At the same time, lead-based PNCs are often considered too unstable in aqueous media to be widely used in biosensing or other medicine-related applications, and as a result, systematic nanotoxicity studies remain scarce. Among these works is the study by Nath et al. [167] who investigated the in vivo toxicity of dual-shell-encapsulated CsPbBr_3_ PNCs sequentially coated with a SiO_2_ shell followed by polystyrene in 18 male BALB/cJ mice aged 5–6 weeks. In a 14-day intravenous toxicity study (at doses of 1 and 50 mg/kg), CsPbBr_3_@SiO_2_@PS showed no detectable toxicity: body weight, hematological parameters, and liver enzyme levels remained unchanged relative to controls, and histopathological examination of the brain, kidney, lung, liver, and spleen revealed no abnormalities. This favorable safety profile is attributed to the dual silica/polystyrene shell effectively suppressing lead leaching (only ~0.7% of total lead released after two weeks), rather than to any intrinsic non-toxicity of the lead-halide core itself. The same research group also demonstrated the use of CsPbBr_3_@SiO_2_ for staining BT-20 cells, as well as for ex vivo tissue staining and in vivo imaging in mice under two-photon excitation [168]. They further found that CsPbBr_3_@SiO_2_ showed no significant toxicity toward BT-20 cells at concentrations up to ~50 µg/mL, even after 48 h of incubation, as assessed by the CCK-8 assay. Mohapatra et al. likewise reported an in vitro cytotoxicity study of silica-coated CsPbBr_3_ PNCs in 3T3L-1 cells [169]. According to MTT assay results, cell viability and morphology remained comparable to the control group after prolonged incubation (24, 48, and 72 h), and the morphology of mitochondria and lysosomes in treated cells did not differ from that in untreated cells—indicating a substantial reduction in PNC toxicity following encapsulation within such protective shells. These data are short-term and do not cover mechanical damage, long-term ageing, or end-of-life handling; luminescence retention after water immersion alone does not establish that lead remains contained. Practical use therefore requires standardized leaching tests after ageing and abrasion, containment in sealed detector housings, and defined recycling routes.

The remainder of this section looks beyond the individual studies discussed above, toward the four performance parameters that most often limit the practical deployment of PNC-based scintillators: optical transparency and spatial resolution, radioluminescence intensity (light yield), scintillation decay time, and stability under humidity, ionizing radiation, and elevated temperature. For each, we summarize recent developments and outline directions that, to our knowledge, remain relatively unexplored for PNC-based scintillators.

*Improving transparency and spatial resolution*

Spatial resolution is governed less by PNC composition than by how effectively light scattering is suppressed between the point of photon emission and the photodetector. Three complementary strategies recur throughout this review: (1) matching the refractive index of the PNC and the host—exemplified by PNCs grown in glass and mesoporous silica hosts—which reduces scattering at the nanocrystal–matrix interface and is part of why these hosts also achieve some of the highest PLQY values reported; (2) geometrically confining light within the emitting volume, exemplified by AAO-templated PNC nanowires and pre-synthesized-PNC-loaded AAO membranes, where spatial resolution reaches 160–250 lp/mm precisely because the alumina walls act as light guides that prevent lateral spread; and (3) reducing PNC aggregation itself through ligand and matrix engineering, since aggregated nanocrystals scatter more per unit mass than well-dispersed ones at the same nanocrystal loading.

A further approach, based on refractive-index contrast rather than index matching, was described in a recent article by Song et al. [170]. The authors embedded a pixelated array of CsPbBr_3_ scintillator elements within a polyurethane acrylate matrix whose refractive index was deliberately mismatched, so that light generated within each pixel is confined by internal reflection rather than allowed to spread into neighboring pixels. This design achieved a resolution of 20.1 lp/cm in computed tomography imaging, with light-collection efficiency roughly twice that of an equivalent unstructured film. This strategy is essentially the inverse of the index-matching approach discussed above for glass-hosted PNCs, and the two have not yet, to our knowledge, been combined: an index-matched PNC/glass composite patterned into a pixel array could in principle suppress scattering at both the nanoscale (PNC–matrix interface) and the mesoscale (pixel-to-pixel level) simultaneously.

*Increasing radioluminescence intensity (light yield)*

The most common levers for improving light yield discussed in this review are surface and dopant engineering to raise PLQY; FRET between PNCs and a classical scintillator or dye (in either direction); increased PNC loading within MOF and silica hosts that reduce concentration quenching; and, most recently, nanoplasmonic Purcell enhancement of PNC fluorescence, in which nearby metal nanoparticles increase the radiative decay rate of the nanocrystals. All of these strategies work by creating more emitting centers, reducing non-radiative loss channels, or shortening the radiative lifetime. None of them, however, directly addresses the self-absorption that occurs whenever a PNC’s own emission spectrum overlaps its own absorption edge—the mechanism that ultimately limits how thick, and therefore how X-ray-absorptive, a PNC scintillator can be made before internal reabsorption cancels out any gain in stopping power.

Two recent strategies target this reabsorption problem directly, rather than working around it with an external dye or heterostructure. Jin et al. showed that the intrinsic lattice strain present in 2D (Ruddlesden-Popper-type) perovskite crystals red-shifts the emission relative to the absorption edge—a “self-wavelength-shifting” effect that widens the effective Stokes shift without requiring any dopant or added chromophore, and without slowing the radiative decay [171]. Alternatively, Hu et al. developed a low-temperature, polar-solvent synthesis that controls the exciton relaxation pathway during nanocrystal formation itself, increasing the Stokes shift and, as a direct consequence, both the light yield (28,800 photons/MeV) and the maximum film thickness achievable before self-absorption losses dominate [172].

Because both approaches modify the PNC’s intrinsic optical properties rather than introducing a second material, they are, in principle, compatible with the matrix, dopant, and FRET strategies already discussed in this review—raising the possibility of combining “internal” Stokes-shift engineering with “external” energy-transfer or Purcell-enhancement strategies. This represents a promising, largely unexplored direction for further gains in light yield.

*Improving decay time*

Decay time is reported in most of the studies discussed here, but in contrast to light yield, resolution, and stability, few studies in this review set out specifically to engineer it; RL and PL lifetime values are typically reported as an outcome of a strategy aimed at some other parameter, rather than as the primary target of the study itself. This represents a genuine gap, since decay time directly limits both the maximum frame rate achievable in dynamic X-ray imaging and the coincidence timing resolution achievable in PET-type applications.

The clearest general principle emerging from this review is a trade-off between light yield and decay time, one that tracks whether emission proceeds via a self-trapped or a free exciton. Self-trapped exciton emission—associated with strong exciton–phonon coupling in Mn^2+^-doped and low-dimensional (0D/2D) perovskites—typically yields high PLQY and a large, self-absorption-suppressing Stokes shift, but the same strong lattice distortion responsible for trapping the exciton also slows its radiative decay, often into the microsecond range, which runs counter to what fast-imaging applications require. Free-exciton emission, by contrast, is fast (sub-nanosecond to a few nanoseconds) but is more susceptible to non-radiative loss and self-absorption.

Liu et al. recently addressed this trade-off directly by engineering the organic A-site cation of a 2D perovskite bulk crystals to enhance in-plane octahedral distortion—which localizes and brightens the exciton—while simultaneously suppressing the out-of-plane and intra-octahedral distortions responsible for self-trapping. The resulting (1,4-CMA)PbBr4 crystal (1,4-CMA = cyclohexane-1,4-diyldimethanamine) combines a 0.62 ns response time with a light yield of 19,700 photons/MeV—a combination the authors report as unmatched by leading commercial and research scintillators to date [173]. This kind of targeted, distortion-selective compositional design—rather than simply comparing decay times across compositions after the fact—represents a promising direction not only for bulk scintillating crystals, but also for nanocrystalline perovskite-based scintillators, by extending this level of control over exciton localization pathways to future material systems.

*Improving stability under humidity, radiation, and elevated temperature*

Among the parameters characterizing PNC-based composites, stability depends most strongly on the choice of host—inorganic glass, silica, polymer, or MOF—used to physically isolate the PNCs from moisture and oxygen, as well as on surface-ligand and post-synthetic passivation strategies and compositional choices such as Cd^2+^ doping for enhanced radiation hardness. Reported stability windows in this review span from tens of hours of humidity or heat exposure for some polymer-hosted PNCs, up to hundreds of hours for glass- and MOF-hosted nanocrystals. In terms of radiation hardness, PNC-based composites have demonstrated tolerance to cumulative doses ranging from the mGy level—typical of medical-imaging doses—up to the MGy range reported for a few solution-synthesized, pre-loaded compositions.

Two further strategies, already well established for PNC applications in optoelectronics (LEDs, photovoltaics), are worth flagging as prospects for scintillator technology. First, post-synthetic surface fluorination has been shown to suppress the surface electron traps that otherwise limit radiation hardness, enabling CsPbBr_3_ nanocrystals to withstand γ-ray doses of up to 1 MGy without significant loss of scintillation yield [174]. This suggests that surface chemistry, independent of the host matrix, remains an underused lever for improving radiation hardness specifically—one that could complement existing matrix-based protection strategies. Second, atomic layer deposition of a thin, conformal Al_2_O_3_ shell has been shown to protect PNCs against combined moisture, oxidation, and thermal stress (60 °C/90% relative humidity) in optoelectronic devices [175]; its atomic-scale thickness control and pinhole-free conformality could in principle offer a more precisely tunable barrier than bulk glass or polymer encapsulation—particularly valuable for thin, high-resolution scintillator films, where a thick glass or MOF shell would compromise flexibility or resolution.

As outlined at the end of Section 2, light yield, decay time, spatial resolution, and stability are rarely optimized independently, and the strategies discussed throughout this review illustrate these trade-offs in practice. For a broader synopsis of these trade-offs, and for further engineering strategies beyond those discussed above, we refer the reader to a recent dedicated review of the field by Liu et al. [176].

The most promising recent results—including several highlighted above—are precisely those that decouple these trade-offs at the level of exciton physics or surface/interface engineering, rather than working around them through an additional bulk component. A further, largely unaddressed obstacle to comparing and cross-analyzing these results is the lack of standardized testing protocols: as noted throughout this review, studies report stability under widely different dose rates, humidity levels, and exposure times, and resolution at different scintillator thicknesses, among other varying conditions—making it difficult to establish which strategies genuinely outperform others under comparable circumstances. Progress on this front, alongside continued exploration of the combined strategies outlined above, will likely determine how quickly PNC-based scintillators move from laboratory-scale demonstrations toward the standardized, mass-manufacturable detectors required for medical, industrial, and scientific X-ray and γ-ray imaging.

## Figures and Tables

**Table 1:** Comparison of organic and inorganic scintillators.

Scintillator Material	*Z*_eff_	Density, g/cm^3^	Light Yield, Photons/MeV	Fluorescence Decay Time, ns
**Inorganic scintillators**				
BGO	73	7.13 ^(a)^	8000 ^(b)^	300 ^(c)^
LYSO:Ce	65	7.2 ^(a)^	27,000 ^(b)^	40 ^(d)^
CsI:Tl	51	4.51 ^(a)^	61,000 ^(b)^	1000 ^(d)^
GAGG:Ce	54.4	6.63 ^(f)^	63,400 ^(b)^	50 ^(e)^
**Organic scintillators**				
Anthracene	5.78	1.28	20,100 ^(h)^	27.6 ^(h)^
Stilbene	5.68	1.16	16,000 ^(h)^	3.4 ^(h)^
EJ-301	5.64	0.874 ^(g)^	12,000 ^(g)^	3.2 ^(g)^
EJ-200	5.67	1.023 ^(g)^	10,000 ^(g)^	2.1 ^(g)^
p-terphenyl	5.76	1.23	19,400 ^(h)^	1.9 ^(h)^

Abbreviations: BGO, bismuth germanate; LYSO, Lutetium–yttrium oxyorthosilicate; GAGG, gadolinium aluminium gallium garnet; EJ-301, Eljen Technology EJ-301 (commercial liquid scintillator); EJ-200, Eljen Technology EJ-200 (commercial plastic scintillator). Ref: (a) [15]; (b) [16]; (c) [17]; (d) [18]; (e) [19]; (f) [20]; (g) [21]; (h) [22].

**Table 2:** Key equations for characterizing radiation attenuation, effective atomic number, and scintillation light yield.

Parameter	Mathematical Expression	Legend	Designation	Ref
Photon beam attenuation	$I=I_{0}e^{-\mu_{m}x},\;\mu_{m}=\sum_{i}\omega_{i}\cdot {\left(\frac{\mu }{\rho }\right)}_{i}$	*I*_0_ and *I* are the initial and transmitted photon intensities, respectively; *x* is the material thickness; *μ_m_* is the mass attenuation coefficient; *ω_i_* is the weight fraction of the *i*-th element; *μ* is the linear attenuation coefficient of *i*-th element; ρ representing the *i*-th material density.	Equation (1)	[49]
Photoelectric scattering cross section for low γ-ray energy (E_γ_ ≪ m_e_c^2^)	σ_incoh_ ∝ *Z*	*Z* is atomic number of the absorber material.	Equation (2)	[50]
Photoelectric scattering cross section for high γ-ray energy (E_γ_ ≫ m_e_c^2^)	$\sigma_{incoh}\propto \frac{Z}{E_{\gamma }}ln\left(\frac{E_{\gamma }}{m_{e}c^{2}}\right)$	*Z* is atomic number of the absorber material; *E*_γ_ is γ-ray energy.	Equation (3)	[50]
Photoelectric absorption cross section for low γ-ray energy (E_γ_ ≪ m_e_c^2^)	$\sigma_{pe}\sim \frac{Z^{N}}{E^{3.5}}$	*Z* is atomic number of the absorber material; *E* is the photon or x-rays energy; *N* is a variable exponent between 3 and 5.	Equation (4)	[51]
Photoelectric coherent (Rayleigh) scattering cross section for low γ-ray energy (E_γ_ ≪ m_e_c^2^)	σ_coh_ ∝ *Z*^2^/*E*_γ_^2^	*Z* is atomic number of the absorber material; *E*_γ_ is γ-ray energy.	Equation (5)	[50]
Photoelectric absorption cross section for high γ-ray energy (E_γ_ ≫ m_e_c^2^)	$\sigma_{pair}\propto Z^{2}\left[\frac{28}{9}ln\left(\frac{2E_{\gamma }}{m_{e}c^{2}}\right)-\frac{218}{27}\right]$	*Z* is atomic number of the absorber material; *E*_γ_ is γ-ray energy.	Equation (6)	[52]
Triplet cross section. Cross section, similarly to the Compton effect, is not related to Z but to the number of electrons per atom, which do not need to be always equal to each other.	$\sigma_{trip}\propto q\alpha r_{e}^{2}\cdot f\left(k\right)$	q is number of electrons per atom; α is fine-structure constant (1/137); $r_{e}^{2}\approx 0.07941\;b$ (square of the classical electron radius); *k* is energy range (photon energy in eV normalized to the electron rest-mass energy).	Equation (7)	[53]
Effective atomic number	$Z_{eff}={\left(\sum \omega_{i}Z_{i}^{4}\right)}^{1/4}$	$\omega_{i}$ and *Z_i_* are the fraction of the total mass associated with the *i*-th element and the atomic number of the *i*-th element, respectively.	Equation (8)	[54]
Effective atomic number for low-Z materials (*Z*_eff_ < 20)	$Z_{eff}={\left(\sum \omega_{i}Z_{i}^{2.94}\right)}^{1/2.94}$	$\omega_{i}$ and *Z_i_* are the fraction of the total mass associated with the *i*-th element and the atomic number of the *i*-th element, respectively.	Equation (9)	[55]
Effective atomic number for mixture materials	$Z_{eff}=\frac{\sum f_{i}A_{i}{\left(\frac{\mu }{\rho }\right)}_{i}}{\sum \frac{f_{i}A_{i}}{Z_{i}}{\left(\frac{\mu }{\rho }\right)}_{i}}$	*f_i_* is the *i*-th element fractional composition by weight in the mixture material; *A_i_* is the atomic mass number of the *i*-th element; $\frac{\mu }{\rho }$ is the mass attenuation coefficient of the *i*-th element.	Equation (10)	[56]
Mass attenuation coefficient	$\frac{\mu }{\rho }\sim \frac{Z^{4}}{AE^{3}}$	μ is X-ray absorption coefficient; *ρ* is material density; *Z* is material atomic number; *A* is atomic mass; *E* is X-ray or γ-ray energy.	Equation (11)	[57]
Radiation length	$X_{0}=\frac{A}{\rho }\frac{716.4\;gcm^{-2}}{Z\left(Z+1\right)ln\left(287/Z\right)}$	*ρ* is material density; *A* is atomic mass; *Z* is atomic number.	Equation (12)	[58]
Absolute light yield	$\eta =\frac{E_{p}}{E}$	*E*_p_ is total energy of scintillation photons; *E* is energy deposited by ionizing radiation in the scintillator.	Equation (13)	[59]
Technical light yield	$T=\frac{L_{p}}{E}$	*L*_p_ is ratio of total energy of scintillation photons, passing through the window of the detector. The collection area being limited by the diameter of the photomultiplier tube; *E* is energy deposited by ionizing radiation in the scintillator.	Equation (14)	
Number of visible photons emitted by the scintillator	$N_{ph}=N_{eh}\cdot S\cdot Q=\frac{E_{\gamma }}{E_{eh}}\cdot S\cdot Q$	*N*_eh_ is number of electron-hole pairs generated upon energy deposition; *S* is the energy-transfer efficiency from the electron-hole pairs to the luminescence centers; *Q* is quantum efficiency of the final radiative process responsible for scintillation emission; *E_γ_* is energy of γ—or X-ray photon; *E*_eh_ is mean energy required to create one thermalized electron-hole pair.	Equation (15)	[60]
	$N_{ph}=\frac{E_{\gamma }}{{\beta E}_{g}}\cdot S\cdot Q$	*E_γ_* is γ- or X-ray energy; *E*_g_ is forbidden gap of the material scintillator; *β* is phenomenological parameter ranges between 2 and 3; *S* is energy-transfer efficiency from the electron-hole pairs to the luminescence centers; *Q* is quantum efficiency of the final radiative process responsible for scintillation emission.	Equation (16)	[61,62]
Light yield (photons per MeV)	$LY=10^{6}\frac{\;\gamma \;QY}{{\beta E}_{g}}\;\;$	*E*_g_ is band-gap energy of the scintillator; *β* is dimensionless parameter ranges between 2 and 3; *γ* is a factor that account for the fraction of X-ray photon energy converted into electronic energy; *QY* is quantum yield of the radioluminescence state.	Equation (17)	[63]

**Table 3:** Summary of the methods and mathematical apparatus used for measurement of scintillation light yield.

Method in Brief	Mathematical Expression	Legend	Designation	Ref
Light yield is determined relative to a reference scintillator with a known *LY* using the spectrally integrated RL intensity. The expression additionally corrects for differences in the fraction of deposited X-ray energy and the irradiated sample area, thereby enabling a quantitative comparison of scintillation efficiencies under identical excitation conditions.	$\frac{LY_{sample}}{LY_{ref}}=\frac{\eta_{ref}\int I_{sample}\left(\lambda \right)d\lambda \cdot S_{ref}}{\eta_{sample}\int I_{ref}\left(\lambda \right)d\lambda \cdot S_{sample}}$	*η* is X-ray deposited energy percentage of the scintillator; *I* represent the radioluminescence intensity at wavelength *λ*; *S* is irradiation area and X-ray intensity is assumed to be uniform across the whole scintillator film.	Equation (18)	[71]
This approach evaluates the relative *LY* between samples of the same scintillator with different thicknesses. A key feature of the method is the correction of the measured RL response for the wavelength-dependent detection efficiency, *S*(*λ*), which minimizes the influence of the detector spectral response on the resulting *LY* ratio.	$\frac{LY_{sample}}{LY_{ref}}=\frac{R_{sample}}{R_{ref}}\cdot \frac{\int I_{ref}\left(\lambda \right)S\left(\lambda \right)d\lambda \;/\int I_{ref}\left(\lambda \right)d\lambda }{\int I_{sample}\left(\lambda \right)S\left(\lambda \right)d\lambda \;/\;\int I_{sample}\left(\lambda \right)d\lambda }$	*R* is the X-ray response voltage (corrected response amplitude of the samples with different thicknesses); *I*(*λ*) is the radioluminescence spectrum; *S*(*λ*) is the wavelength dependent detection efficiency of Silicon Photomultiplier (SiPM).	Equation (19)	[68,72]
The distinctive feature of this comparative method is the correction of the measured emission for differences in X-ray attenuation, sample thickness, and refractive index between the investigated and reference scintillators.	$\frac{LY_{sample}}{LY_{ref}}=\left(\frac{1-e^{-t_{ref}/\alpha_{ref}}}{1-e^{-t_{sample}/\alpha_{sample}}}\right)\cdot \left(\frac{D_{sample}}{D_{ref}}\right)\cdot \left(\frac{n_{sample\;}}{n_{ref}}\right)$	ref denotes the reference scintillator; *α* is the attenuation length necessary for X-ray intensity to be reduced to 1/*e*; *t* is the thickness of scintillator; *D* is the integrated area under the emission curve; *n* is the index of refraction.	Equation (20)	[63]
This reference-based method determines the *LY* from radioluminescence spectra while correcting for differences in X-ray energy deposition, detector spectral response, and irradiated area between the sample and the reference scintillator.	$\frac{{LY}_{ref}}{{LY}_{sample}}=\frac{\eta_{ref}}{\eta_{sample}}\times \frac{\int I_{sample}\left(\lambda \right)S\left(\lambda \right)d\lambda /\int I_{sample}\left(\lambda \right)d\lambda }{\int I_{ref}(\lambda )S\left(\lambda \right)d\lambda /\int I_{ref}(\lambda )d\lambda }\times \frac{S_{ref}}{S_{sample}}$	*η* is the X-ray deposited energy percentage of scintillators; *I* is the radioluminescence intensity at different wavelength (*λ*); $S\left(\lambda \right)$ is detection efficiency of the detector; *S* is the area exposed to X-ray of scintillators and the X-ray intensity is assumed to be uniform across the whole scintillator film.	Equation (21)	[73]
The distinctive feature of this approach is the normalization of the measured photon counts to the X-ray attenuation efficiency before comparison with a reference scintillator of known *LY*.	$\frac{LY_{sample}}{LY_{ref}}=\frac{PC_{normalized}\left(sample\right)}{PC_{normalized}\left(ref\right)},\;PC_{normalized}=\frac{PC_{measured}}{AE\left(d\right)}$	*AE*(*d*) is the X-ray attenuation efficiency (%) of the scintillator at a thickness of 1 mm; *PC*_measured_ is the measured photon count (that is, radioluminescence spectra were then integrated to obtain the corresponding photon counting). The number of emission photons in the scintillator shall be normalized to the same X-ray attenuation (100%) as *PC*_normalized_	Equation (22)	[67]
This reference-based method determines the *LY* from the measured Compton-edge position while correcting for the spectral matching between the scintillator emission and the photomultiplier response.	$\frac{LY_{sample}}{LY_{ref}}=\frac{{CE}_{sample}}{{CE}_{ref}}\cdot \frac{\Phi_{sample}}{\Phi_{ref}}$	*CE* is channel number of the Compton edge; Φ is photomultiplier tube sensitivity correction factor that describes the spectral match between the scintillator emission and photocathode response.	Equation (23)	[74,75]
Direct *LY* determination. The distinctive feature of this approach is the direct determination of *LY* from the measured light output with explicit corrections for photon detection efficiency (*PDE*), light-transfer efficiency (*LTE*), and detector-related effects.	$LY=\frac{1}{PDE\cdot NP\cdot LTE}\cdot \frac{1}{\langle E_{n}\rangle \left[keV\right]}\cdot \frac{LO\left[nWb\right]}{SPe\left[nWb\right]}\cdot \frac{1}{CT}$	*PDE* is the photodetection efficiency; *NP* is the non-proportionality, which would allow extrapolating the intrinsic *LY*; *LTE* is the light transfer efficiency; *SPe* is the calibration for single photoelectron pulse; <*E*_n_> is the mean deposited energy [keV]; *LO* (light output), where *LO*/*SPe* is the factor converts the measured integrated charge *LO* into the equivalent number of detected photoelectrons/triggered single-photon avalanche diodes; *CT* is the correction factor accounting for SiPM crosstalk (corresponding to the excess charge factor).	Equation (24)	[76]

**Table 4:** The performance of scintillators based on in situ-grown PNCs.

PNC	Dopants/Additional Elements	Matrix	Light Yield, Photons/MeV	Flexible	RLLT	PLLT	PLQY	Stability	Spatial Resolution	Sensitivity	Ref.
**Scintillators with pristine PNC**											
CsPbBr_3_	-	PMMA	15,800	yes	-	-	-	8 mGy/s, 3 h, 50 cycles—stable	up to 12.5 lp/mm	120 nGy/s	[95]
MAPbBr_3_	-	PET	23,000	yes	186.5 ns	15.25 ns	-	3 days, 85 °C in water—94% PL retention; Recovery after heating and cooling: 150 °C cooling—100% PL, 200 °C cooling—48% PL, 167 °C -27 °C three heating/cooling cycles—100% RL	-	-	[97]
Cs_3_Cu_2_I_5_	-	PVA with vitamin B1	55,521	yes	-	4.766 μs	60.73%	30 days in air + UV—95% PL; 2.29 mGy/s, 20 on/off cycles of 5 min each—stable	14.0 lp/mm	4.3 μGy_air_/s	[99]
CsPbBr_3_	-	PMMA with vitamin B1	9001	yes	12.99 ns	-	-	480 h in aqueous solution—96.5% PL retain; Heating to 90 °C—34% PL retain	9.0 lp/mm	-	[100]
CsPbBr_3_	-	nylon filter membrane	-	yes	-	11.8 ns	79.2%	288 h at 30–50% humidity—100% PL	10.4 lp/mm	1.2 μGy/s	[101]
CsPbBr_3_	-	ZBSG	4500	no	15.2 ns	short/long components 2.6/13.0 ns	28.9%	In water PL decrease by 17% after 10 h, then unchanged for 2 weeks	-	-	[103]
CsPbBr_3_	-	mesoporous silica	-	no	-	fast/long components 8.32/43.59 ns	93.2%	Heating to 60 °C—84% PL retain, to 100 °C—41% PL retain; 250 h in water—100% PL retain; 48 h light (3500 W/m^2^)—100% PL retain; 60 °C + 90% humidity + light (3500 W/m^2^), 1000 h—90% PL retain	16 lp/mm	339 nGy_air_/s	[104]
CsPbBr_3_	-	glassy coordination polymer	1202	yes	-	-	59.86%	-	25 lp/mm	514 nGy/s	[106]
CsPbBr_3_	-	PP	14,189	yes	-	43 ns	78%	90 min under 40 kV tube voltage and 800 μA tube current—stable	-	-	[102]
CsPbBr_3_ NW	-	AAO	5300	no	-	3.7–8.8 ns (depends on NW thickness)	-	Stable PL after ambient storage conditions for 2 months; 6.77 Gy_air_/h, 24 h—about 70–80% RL	160 lp/mm	-	[107]
CsPbBr_3_	-	AAO	11,100	no	-	9 ns (for PNC solution)	83%	Ambient conditions (relative humidity is about 45%) 45 days—60% PL	250 lp/mm	-	[109]
**Scintillators with doped PNC**											
CsPbBr_3_	Eu^3+^ (1.5%)	ZBSG	10,100	no	-	6.78 ns	65%	189 μGy_air_/s, 100 h 100% RL retain; 100 h at 85 °C 100% RL retain	15 lp/mm	-	[85]
CsPbBr_3_	Eu^3+^ (0.6%)	ZBSG	-	no	-	49.17 ns	58.6%	105 μGy_air_/s, 25 h 100% RL; 5 days in water—stable	-	-	[113]
CsPbBr_3_	Ce^3+^ (0.3%)	BSG	-	no	-	21.6 ns	28%	humid environment for 100 days (RH = 90%)—stable	-	-	[114]
CsPbBr_3_	Gd^3+^ (4%)	BSG	9% of BGO	no	-	44 ns	37.7	6 cycles heating to 480 °C—full RL recovery	12.7 lp/mm	-	[115]
**Scintillators with PNC and additional functional elements**											
CsPbBr_3_	Ag nanocluster (1%)	BPG	-	no	-	13.62 ns	43%	-	-	-	[119]
CsPbBr_3_	Cs_4_PbBr_6_, MOF	PMMA	21,760	yes	-	<20 ns	70.2%	2 h, total dose 7.2 Gy -88% RL; 24 h, 85% humidity and 85 °C >80% RL	18 lp/mm	132 nGy_air_/s	[120]
CsPbBr_3_	Tb-BTC MOF	PS	24,800	no	10 μs (dominated by Tb^3+^ emission)	55.2 ns	92%	humidity tolerance—low; 30 min, 7 cycles 10 mGy/s—>95% RL	20 lp/mm	-	[42]

Abbreviations: AAO, anodic aluminum oxide; BGO, bismuth germanate; BPG, borophosphate glass; BSG, borosilicate glass; BTC, benzene-1,3,5-tricarboxylate; lp/mm, line pairs per millimeter; MA, methylammonium; MOF, metal–organic framework; NW, nanowire; PET, polyethylene terephthalate; PL, photoluminescence; PLLT, photoluminescence lifetime; PLQY, photoluminescence quantum yield; PMMA, poly(methyl methacrylate); PNC, perovskite nanocrystal(s); PP, polypropylene; PVA, polyvinyl alcohol; RH, relative humidity; RL, radioluminescence; RLLT, radioluminescence lifetime; ZBSG, zinc borosilicate glass.

**Table 5:** The performance of scintillators based on pre-synthesized PNCs.

PNC	Dopant/Additional Element	Matrix	PNC Formation Method	Light Yield, Photons/MeV	Flexible	RLLT	PLLT	PLQY	Stability	Spatial Resolution	Sensitivity	Ref
**Scintillator with pristine PNC**												
CsPbCl_x_Br_(1−x)_	-	PMMA	commercially available from Quantum Solutions	CsPbCl_1.5_Br_1.5_—100%; CsPbClBr_2_—350%; CsPbBr_3_—413%	yes	CsPbCl_1.5_Br_1.5_—30.6 ns; CsPbClBr_2_—33.9 ns; CsPbBr_3_—27.7 ns	CsPbCl_1.5_Br_1.5_—11.4 ns; CsPbClBr_2_—12.2 ns; CsPbBr_3_—9.3 ns	-	-	-	-	[121]
CsPbBr_3_	-	PDMS	hot-injection	-	yes	44.6 ns	-	58.6%	8000 s, X-ray excitation at 30 kV—stable	2 lp/mm	13 nGy/s	[122]
CsPbBr_3_	-	methacrylate	hot-injection	- *	yes	-	2.87 ns	86%	Cumulative dose rate > 40 Gy_air_—96% RL	9.8 lp/mm	-	[129]
CsPbBr_3_	-	PMMA	hot-injection	-	yes	-	8.25 ns	-	4 days at 5.5 μGy_air_/s—>97% 105 days in water—>80% PL	up to 17.3 lp/mm (for 10 µm film)	40.1 nGy_air_/s	[127]
CsPbBr_3_	-	PMMA	ligand-assisted reprecipitation	-	yes	1.81 ns	-	-	1.5 kGy > 95% RL	-	-	[44]
CsPbBr_3_	-	PMMA	ligand-assisted reprecipitation	860	yes	1.4 ns	2 ns	-	-	-	-	[128]
CsPbBr_3_	-	EVA	hot-injection	-	yes	-	14.19 ns	80%	Ten cycles–18 °C/+50 °C—>95% RL; 1500 bent cycles—stable PL; 10 h in water—stable	-	-	[131]
CsPbBr_3_	-	MMA/LMA	turbo-emulsification	up to 4800	yes	up to 1.13 ns	-	90%	1000 kGy—>80% PLQY	-	-	[69]
CsPbBr_3_	-	PMMA	antisolvent	6000	no	490 ps	-	84%	-	-	-	[133]
CsPbBr_3_	-	APTES/APTES-PA	ligand-assisted coprecipitation	-	yes	-	22.7 ns	80.6%	180 °C—62.3% PL retain; UV (1.5 mW/cm^2^) 6 h—78.9% PL; water, 6 h—stable 180 min 6.58 mGy/s—stable	20 lp/mm	-	[43]
CsPbBr_3_	-	PS + AHFS/ATMOS	hot-injection	-	yes	-	-	60%	ambient (dark) conditions, 50 days– stable; 110 °C—stable	-	-	[135]
CsPbBr_3_	-	PS	hot-injection	-	yes	1.5 ns	-	-	-	-	-	[136]
CsPbBr_3_	-	PMMA	Mechanochemical Synthesis/ball milling method	about 30% of LYSO:Ce scintillator	no	29.4 ns			60 days in water—stable	8 lp/mm		[139]
**Scintillators with doped PNC**												
CsPb_0.89_Cu_0.11_Br_3_	Cu^2+^	PCL	microfluidic	21,340	yes	-	-	-	1000 bending cycles—90% PL; 250 μGy/s, 30 min—stable RL	-	82.5 nGy/s	[141]
CsPbBr_3_	Ce^3+^ (8%)	PVDF/PS	hot-injection	33,000	yes	-	-	-	1 month under ambient conditions > 95%; 4 μGy/s, 20 min—92% RL	580 lp/mm	-	[142]
Cs_3_Cu_2_I_5_	Zn^2+^	EVA	hot-injection	-	yes	-	3.7 μs for solution of PNC	67.2%	10 days on air—95% PL; 0.2 mGy_air_/s, 60 min—stable	15.7 lp/mm	0.31 μGy_air_/s	[144]
CsCdCl_3_	Mn^2+^ (0.25)	PMMA	hot-injection	54,500	yes	-	18.75 ms in solution	82.7% for PNC in solution	5.7 mGy/s, 1.5 h—RL stable	17.5 lp/mm	28.97 nGy/s	[145]
CsPbCl_3_	Cd^2+^ excess	PLMA/EGDM	ligand-assisted reprecipitation assisted by turbo-emulsification	2100	-	-	210–300 ps	93% for PNC in solution	1 MGy—RL stable	-	-	[81]
**Scintillators with PNC and additional functional elements**												
CsPbBr_3_	GAGG, CdWO_4_ or LYSO	PMMA	commercially available	+51% for GAGG +36% for LYSO; +31% for CdWO_4_	no	-	-	-	-	-	-	[149]
FAPbBr_3_	EJ-290	PVT	ligand-assisted reprecipitation	1.9 times higher than for EJ-290 scintillator	no	-	decrease with PNC concentration increase	-	-	-	-	[150]
CsPbBr_3_	IH type ZnS(Ag)	PE	solid-phase method	40,000	yes	36 ns	-	-	7 mGy_air_/s, 175 min—stable	30 lp/mm	70 nGy_air_/s	[151]
CsPbBr_3_	Organic dye perylene dyad	PMMA	ligand-assisted reprecipitation	9000	no	-	3.4 ns	-	800 Gy > 85% RL	-	-	[154]
CsPbBr_3_	Ag SNP/CNP	PDMS	hot-injection	8500 (for CNP)	no	4.18 ns (for SNP); 3.70 ns (for CNP)	1.61 ns (for SNP); 0.94 ns (for CNP)	55% in solution	-	5 lp/mm	-	[160]
**Scintillators with PNC without matrix**												
CsPbBr_3_ nanosheets	-	no	single-step injection at room temperature	21,000 in solution	no	-	2–13 ns with varied wavelengths from 460 to 550 nm	-	1 month in ambient conditions; 18 μGy/s, 2 h—RL stable	0.21 mm	-	[161]
CsPbBr_3_ nanosheets	-	no	ligand-assisted reprecipitation	-	no	-	-	88.9%	19 μGy/s, 1 h—RL stable; 2 months ambient conditions—60% of initial PLQY	26 µm	27 nGy/s	[163]
Cs_3_Cu_2_I_5_	-	no	chemical vapor deposition (CVD)	39,251	no	-	1.12 μs	85.14%	0.2 mGy_air_/s, 1.5 h—RL stable; 60 days air—58.32% PLQY	100 lp/mm	0.815 μGy/s	[164] †
CsPbBr_3_	-	AHFS	hot-injection	up to 290,000 photons per MeV under proton beams	possible	336 ps under proton-beam	-	71%	proton fluence 10^11^ per cm^2^, 120 min—stable	lateral resolution of 23.1 nm horizontally and 39.7 nm vertically for proton imaging		[166]
0D Cs_4_PbBr_6_	-	GDRB	antisolvent	10% of EJ232	no	about 1 ns	-	-	13 days in air—stable	-	-	[134]

Abbreviations: AHFS, ammonium hexafluorosilicate; APTES, (3-Aminopropyl)triethoxysilane; APTES-PA, (3-Aminopropyl)triethoxysilane with pentanedioic anhydride; ATMOS, allyltrimethoxysilane; CNP, cuboid nanoparticle; CVD, chemical vapor deposition; EJ-290, Eljen Technology EJ-290 (commercial plastic scintillator); EJ232, Eljen Technology EJ-232 (commercial scintillator); EGDM, ethylene glycol dimethacrylate; EVA, ethylene vinyl acetate; FA, formamidinium; GAGG, gadolinium aluminum gallium garnet; GDRB, GENESIS-Development Resin Base; LMA, lauryl methacrylate; lp/mm, line pairs per millimeter; LYSO, lutetium yttrium orthosilicate; MMA, methyl methacrylate; PCL, poly(ε-caprolactone); PDMS, polydimethylsiloxane; PE, polyethylene; PL, photoluminescence; PLLT, photoluminescence lifetime; PLMA, poly(lauryl methacrylate); PLQY, photoluminescence quantum yield; PMMA, poly(methyl methacrylate); PS, polystyrene; PVDF, polyvinylidene fluoride; PVT, polyvinyltoluene; RL, radioluminescence; RLLT, radioluminescence lifetime; SNP, spheroid nanoparticle. *—Ref. [129] reports LY value of 177,000 photons/MeV for CsPbBr_3_ (and 66,000 for GOS), calculated from the band gap using LY = 10^6^/(2.5 × E_g_); †—This is a polycrystalline perovskite-related film rather than an ensemble of nanocrystals; we include it as a reference point for matrix-free architectures.

## Data Availability

No new data were created or analyzed in this study. Data sharing is not applicable to this article.
